# Ribosome-bound Get4/5 facilitates the capture of tail-anchored proteins by Sgt2 in yeast

**DOI:** 10.1038/s41467-021-20981-3

**Published:** 2021-02-04

**Authors:** Ying Zhang, Evelina De Laurentiis, Katherine E. Bohnsack, Mascha Wahlig, Namit Ranjan, Simon Gruseck, Philipp Hackert, Tina Wölfle, Marina V. Rodnina, Blanche Schwappach, Sabine Rospert

**Affiliations:** 1 Institute of Biochemistry and Molecular Biology, University of Freiburg, Freiburg, Germany; 2 BIOSS Centre for Biological Signaling Studies, University of Freiburg, Freiburg, Germany; 3 Department of Molecular Biology, University Medical Center Göttingen, Göttingen, Germany; 4 Cluster of Excellence “Multiscale Bioimaging: from Molecular Machines to Networks of Excitable Cells” (MBExC), Göttingen, Germany; 5 Max-Planck Institute for Biophysical Chemistry, Göttingen, Germany

**Keywords:** Chaperones, Membrane proteins, Ribosomal proteins

## Abstract

The guided entry of tail-anchored proteins (GET) pathway assists in the posttranslational delivery of tail-anchored proteins, containing a single C-terminal transmembrane domain, to the ER. Here we uncover how the yeast GET pathway component Get4/5 facilitates capture of tail-anchored proteins by Sgt2, which interacts with tail-anchors and hands them over to the targeting component Get3. Get4/5 binds directly and with high affinity to ribosomes, positions Sgt2 close to the ribosomal tunnel exit, and facilitates the capture of tail-anchored proteins by Sgt2. The contact sites of Get4/5 on the ribosome overlap with those of SRP, the factor mediating cotranslational ER-targeting. Exposure of internal transmembrane domains at the tunnel exit induces high-affinity ribosome binding of SRP, which in turn prevents ribosome binding of Get4/5. In this way, the position of a transmembrane domain within nascent ER-targeted proteins mediates partitioning into either the GET or SRP pathway directly at the ribosomal tunnel exit.

## Abstract (web summary)

The guided entry of tail-anchored proteins (GET) pathway assists in the delivery of such proteins to the ER. Here, the authors reveal that the pathway components Get4/5 probe a region near the ribosomal exit tunnel. Upon emergence of a client protein, Get4/5 recruits Sgt2 and initiates the targeting phase of the pathway.

## Introduction

Integral membrane proteins that follow the secretory and endocytic pathways are synthesized on cytosolic ribosomes and targeted to the membrane of the endoplasmic reticulum (ER). A general challenge during the targeting of membrane proteins is aggregation of hydrophobic transmembrane (TM) domains prior to insertion into the lipid bilayer. In the case of ER-targeted proteins, aggregation is prevented by sophisticated machineries, which selectively recognize and shield TM domains in the cytosol^1–5^.

Many ER membrane proteins are targeted cotranslationally with the assistance of the highly conserved signal recognition particle (SRP)^1–5^. Eukaryotic SRP is a multi-subunit ribonucleoprotein complex, which binds to translating ribosomes exposing nascent SRP recognition sequences comprising N-terminal signal sequences and signal anchor (SA) sequences. The latter initially serve as targeting signals and ultimately as TM domains that anchor mature ER-targeted proteins within the membrane. Ribosome-bound SRP is positioned such that its 54 kDa subunit termed Srp54 binds to emerging SRP recognition sequences in close proximity to the polypeptide exit tunnel of the ribosome. The complex consisting of SRP and the ribosome-bound nascent chain (RNC) is subsequently targeted to the ER membrane where SRP interacts with the SRP receptor, resulting in SRP release and docking of the translating ribosome to the ER translocon. As translation proceeds, the nascent membrane protein is inserted into the ER membrane^1–5^.

The SRP-dependent mode of targeting elegantly circumvents aggregation of TM proteins, as it couples protein synthesis with membrane insertion of TM domains. However, a specific sub-group of TM proteins cannot enter the cotranslational, SRP-dependent pathway, because these proteins possess only a single TM domain at their very C-terminus, termed a tail-anchor (TA) sequence. TA sequences become exposed to the outside of the polypeptide exit tunnel only after the nascent polypeptide is released from the ribosome upon translation termination. The pathway responsible for targeting of TA proteins to the ER membrane is termed the guided entry of TA protein (GET) pathway^6–9^. According to current models, the GET pathway is initiated shortly after release of TA proteins from the ribosome, when the hydrophobic TA sequence is captured by the C-terminal domain of a homodimeric chaperone-like protein termed Sgt2^10,11^. Within the Sgt2/TA protein complex, the N-terminal domain of Sgt2 recruits the Get5 subunit of the heterotetrameric Get4_2_Get5_2_ complex (Get4/5), allowing, in turn, binding of the homodimeric targeting factor Get3 to Get4^10–13^. After the TA sequence is transferred from Sgt2 to Get3 and the Get3/TA protein complex dissociates from the pre-targeting complex, the TA protein is delivered to the Get1/2 insertase in the ER membrane^1,5,11,14–16^.

Considering the relatively recent discovery of the GET pathway, it is remarkable how well many of its features are understood at structural and mechanistic levels, including TA protein capture by Get3 and subsequent membrane delivery^1,5,11,14–17^. However, our understanding of the initial phase of the GET pathway lags behind. SRP scans translating ribosomes and displays enhanced binding affinity even before nascent TM domains become fully exposed outside of the exit tunnel^4,18–22^. In contrast, precisely where and when Sgt2 captures TA proteins and why Get3 does not bind directly to released TA proteins is currently not understood. This may be due to the immense experimental challenge of analyzing the short period between peptidyl-tRNA cleavage upon translation termination and complete exit of released polypeptides from the ribosomal tunnel.

Preliminary evidence suggests that, even though the GET pathway is considered to employ a posttranslational mechanism, it may be directly coupled to the emergence of its clients from the ribosome. A high-throughput study identified Get5 as a ribosome-associated protein^23^ and later work revealed that indeed the yeast Get4/5 complex binds to ribosomes independently of their translational status, however, displays enhanced affinity to those ribosomes with a TM sequence inside the ribosomal tunnel^24^. These findings hint at a direct transfer of TA proteins from the ribosome to the GET pathway, with Get4/5 potentially being the earliest sensing component. Such a scenario, however, is difficult to reconcile with current models, which appear well supported by extensive in vitro reconstitution approaches and consider Sgt2 as the first GET pathway component interacting with TA proteins^1,5,11,14–16^.

Here we investigated the early steps of the GET pathway, with a focus on the steps that occur upon exit of TA sequences from the polypeptide exit tunnel of the ribosome. We show that Get4/5 binds to ribosomes with high affinity via the Get5 subunit, which contacts ribosomal proteins Rpl26/uL24 and Rpl35/uL29 at the exit of the ribosomal tunnel (names of ribosomal proteins are given according to the yeast standard nomenclature). Get4 is not required for stable ribosome-binding of Get5, but binds near h46/h47 of the 25S rRNA in the complex. The ribosomal binding site of Get5 overlaps with that of SRP, and SRP can prevent Get4/5 from binding to the ribosome. However, once Get4/5 is bound to the ribosome, Sgt2 is recruited to the tunnel exit and gains direct access to exposed TA sequences. This Get4/5-dependent recruitment of Sgt2 improves the efficiency by which Sgt2 captures TA proteins upon release from the ribosome. The combined data indicate that ribosome-bound Get4/5 couples translation of TA proteins to their entry into the GET pathway, thereby minimizing cytosolic exposure of TA sequences.

## Results

### The Get4/5 complex interacts with ribosomes via Get5

**Fig. 1:**
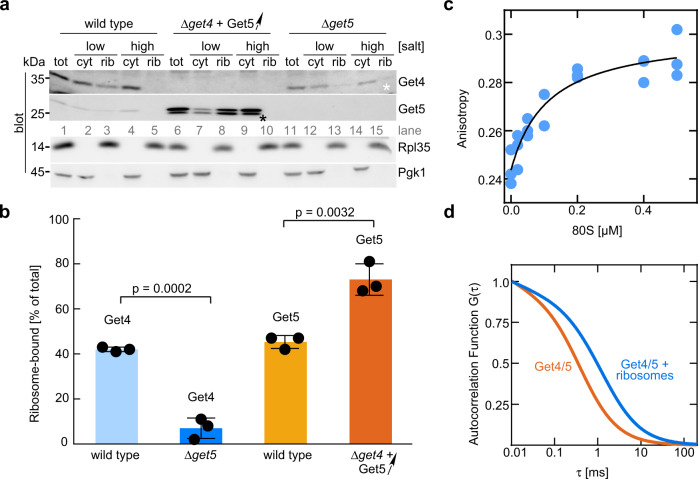
The Get4/5 complex binds to ribosomes with high affinity. **a** The Get4/5 complex binds to ribosomes via Get5. Total cell extracts (tot) of wild type, a Δ*get4* strain overexpressing Get5 (Δ*get4* + Get5↑, Supplementary Fig. 1), or a Δ*get5* strain were separated into a cytosolic fraction (cyt) and a ribosomal pellet (rib) under low salt (120 mM KOAc) or high salt (800 mM KOAc) conditions. The black asterisk indicates the fraction of overexpressed Get5 migrating with lower molecular mass, likely due to partial degradation of overexpressed Get5. The white asterisk indicates the fraction of aggregated Get4, which sediments together with ribosomes under high salt conditions in the absence of Get5. Aliquots were analyzed via immunoblotting with α-Get4, α-Get5, α-Rpl35 (ribosomal marker), and α-Pgk1 (cytosolic marker). **b** Quantification of the fraction of Get4 and Get5 bound to ribosomes. Given is the fraction of Get4 or Get5 recovered in low salt ribosomal pellets (rib) compared with the total (rib+cyt) based on the analysis of experiments as shown in **a**. Get4 or Get5 recovered in high salt ribosomal pellets was subtracted as a background. The results of each biological replicate are shown as black dots. Error bars indicate the standard deviation of the mean. Indicated *p* values were calculated using a two-sided Student’s *t* test with GraphPad Prism. **c** Get4/5 binds to purified 80S ribosomes with high affinity. Fluorescence anisotropy-based binding assays were performed with Get4/5-Atto390 (20 nM) and purified 80S ribosomes (see Methods). The data were fit to a one site, saturation binding curve, from which the equilibrium dissociation constant and the standard error of the mean (*K*_d_ = 110 ± 40 nM) was determined using GraphPad Prism. **d** Get4/5 binding to the 80S ribosome monitored by FCS. Autocorrelation functions are represented for Get4/5-Atto655 alone (red) and in complex with the 80S ribosomes (blue). Concentrations of Get4/5-Atto655 and 80S ribosomes were 5 nM and 1 µM, respectively.

The cytosolic Get4/5 complex is distributed between a free and a ribosome-bound pool (Fig. 1a, b, and ref. ^24^). To understand whether one or both subunits of Get4/5 are required for ribosome binding, recruitment of Get4 to the ribosome was analyzed in a Δ*get5* strain, and Get5 was analyzed in a Δ*get4* strain. As Get5 is unstable in a Δ*get4* strain (Supplementary Fig. 1), Get5 was overexpressed in the Δ*get4* background (Fig. 1a, Δ*get4* + Get5↑). Ribosome binding was assessed in a sedimentation assay by separating ribosomes from cytosolic components under low salt (120 mM KOAc) or high salt (800 mM KOAc) conditions. In this assay, ribosome-bound factors cosediment with ribosomes under low-salt conditions, but are released to the cytosolic supernatant when the salt concentration is high. The assay was employed to distinguish ribosome association from protein aggregation, because protein aggregates cosediment with core ribosomal particles under low as well as high salt conditions^25^. In the wild type, >40% of Get4/5 was ribosome-bound under low salt conditions and was released from the ribosome under high salt conditions (Fig. 1a, b, wild type). When Get4 was absent, >70% of overexpressed Get5 was ribosome-bound in a salt-sensitive manner (Fig. 1a, b, Δ*get4* + Get5↑). When Get5 was absent, a significant amount of Get4 was recovered in the pellet even under high salt conditions, suggesting partial aggregation of Get4 (Fig. 1a, white asterisk). The fraction of Get4, which was recovered in the ribosomal pellet in a salt-sensitive manner in the absence of Get5 was strongly reduced to ~7% (Fig. 1a, b, Δ*get5*). We conclude that Get5 interacts with ribosomes stably and independently of Get4, whereas Get4 by itself associates with ribosomes with much lower affinity and requires Get5 for efficient ribosome binding.

### Get4/5 binds to non-translating ribosomes with high affinity

In order to determine the affinity of Get4/5 to 80S ribosomes, the Get4/5 complex was purified, Get4 was labeled with a fluorescent dye Atto390 (Get4/5-Atto390, see Methods) and the binding was monitored using the change of the anisotropy of the fluorophore. Fluorescence anisotropy changes followed a saturation binding curve reflecting a single binding site with a *K*_d_ of 110 ± 40 nM (Fig. 1c). To monitor the interaction between Get4/5 and the ribosome directly, single molecule fluorescence correlation spectroscopy (FCS) was used (Fig. 1d and Supplementary Table 1). FCS reveals the diffusion times of molecules as they move through the confocal volume of a microscope. As the 80S ribosome (3.2 MDa^26^) is much larger than Get4/5 (120 kDa, α_2_β_2_ complex), the diffusion time of Get4/5 alone will be much faster than when it is in complex with the ribosome. For FCS experiments, Get4/5 was labeled with Atto655 and the diffusion properties of Get4/5-Atto655 were studied with or without ribosomes. Fitting the FCS autocorrelation functions showed that in the sample with Get4/5-Atto655 alone, the majority of the molecules moved with an average diffusion time of 0.39 ms; a small fraction of molecules that diffused more rapidly likely represented residual free dye (Supplementary Table 1). From the diffusion time of Get4/5-Atto655, we calculated a diffusion coefficient of 87 ± 2 μm^2^ s^−1^, which is close to the value of 70 μm^2^ s^−1^ estimated based on the size^27^ and hydrodynamic radius^28^ of Get4/5. Addition of 80S ribosomes changed the diffusion time dramatically to approximately 1.6 ms for 67% of Get4/5-Atto655 molecules, reflecting Get4/5/80S complex formation. From the diffusion time, we calculated a diffusion coefficient of 20 μm^2^ s^−1^, which is close to that expected for diffusion of the 80S ribosome (12–18 μm^2^ s^−1^)^29,30^. Together, the data confirm that Get4/5 binds to ribosomes directly and indicate that the affinity of Get4/5 for empty ribosomes is in the submicromolar range (see also Supplementary Note 1).

### Get5 contacts Rpl35 and Rpl26 at the ribosomal tunnel exit

**Fig. 2:**
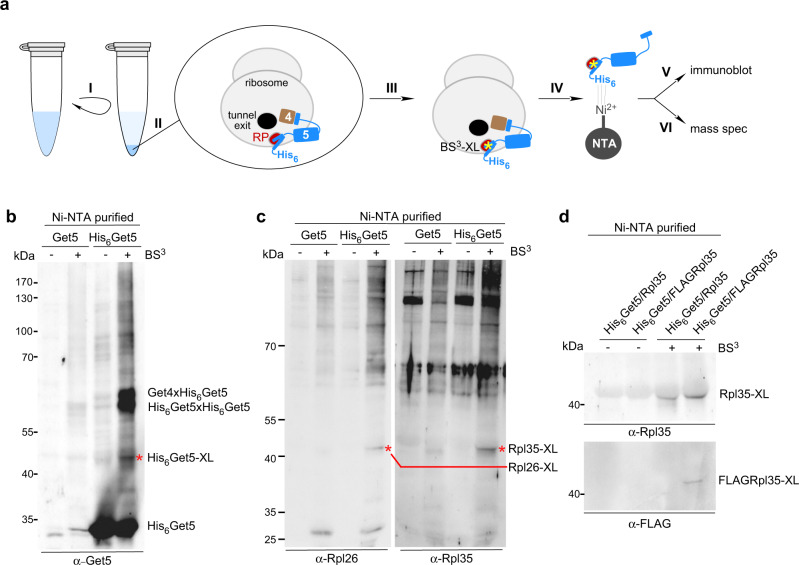
Ribosome-bound Get5 contacts Rpl26 and Rpl35 at the ribosomal tunnel exit. **a** Crosslinking analysis of ribosomal protein contacts. Ribosomes in total cell extract were (I) collected via centrifugation, (II) resuspended in low salt buffer, and (III) crosslinked with the homobifunctional, amino reactive crosslinker BS^3^. (IV) The crosslinked material was denatured and subsequently His_6_Get5-containing crosslink products were isolated via Ni-NTA purification (see Methods). His_6_Get5 and crosslinked proteins, termed Ni-NTA purified His_6_Get5, was analyzed (V) via immunoblotting with antibodies recognizing candidate ribosomal proteins and (VI) via mass spectrometry (see Supplementary Fig. 2). For simplicity, only one heterodimeric subcomplex of the Get4_2_Get5_2_ tetramer is depicted in all cartoons. **b**, **c** Immunodetection of Get5 crosslink products. His_6_Get5, with or without crosslinking, was purified via Ni-NTA as described in **a** from cells expressing either His_6_Get5, or, as a control, untagged Get5. Aliquots of the indicated samples were analyzed via immunoblotting with α-Get5 (**b**), α-Rpl26, or α-Rpl35 (**c**). Red asterisks indicate crosslink products, recognized by α-Get5 (His_6_Get5-XL in **b**), α-Rpl26, or α-Rpl35 (Rpl26-XL, Rpl35-XL in **c**). Crosslink products between His_6_Get5 and Get4 subunits are indicated (see also Supplementary Fig. 2b). **d** Identification of Rpl35 as a crosslink partner of Get5. Ni-NTA purified His_6_Get5 was generated as described in panel **a** from cells coexpressing His_6_Get5 and FLAG-tagged Rpl35 (FLAGRpl35). Aliquots were analyzed via immunoblotting with α-Rpl35 (upper panel) or α-FLAG (lower panel).

We next focused on the identification of contacts between Get5 and ribosomal proteins using a crosslinking approach (Fig. 2a). To enhance the occupancy of ribosomes with Get4/5 during crosslinking, we overexpressed Get4 and an N-terminally His_6_-tagged Get5 variant (His_6_Get5), or, as a control, Get4 and untagged Get5. Ribosomes from these strains were isolated under low salt conditions, crosslinking was performed, and His_6_Get5 crosslink products were purified under denaturing conditions via Ni-NTA (Fig. 2a and Supplementary Fig. 2a). Analysis of the Ni-NTA-purified material revealed several His_6_Get5 crosslink products in the low and high molecular mass ranges (Fig. 2b). The most prominent crosslink products comprised the complex of two Get5 moieties (His_6_Get5xHis_6_Get5) and the complex of Get4 and Get5 (Get4xHis_6_Get5) (Fig. 2b and Supplementary Fig. 2b). The same approach was used for cells overexpressing His_6_-tagged Get4 (His_6_Get4) and untagged Get5; however, no additional crosslink products were detected besides the His_6_Get4xGet5 product (Supplementary Fig. 2b). His_6_Get5 crosslink products were initially probed with antibodies from our collection (α-RPP0/uL10, α-Asc1/RACK1, α-Rps3/uS3, α-Rps9/uS4, α-Rps20/uS10, α-Rpl4/uL4, α-Rpl17/uL22, α-Rpl31/eL31, α-Rpl25/uL23, α-Rpl16/uL13, α-Rpl26/uL24, and α-Rpl35/uL29, see Source Data file). This analysis revealed that α-Rpl26 and α-Rpl35 recognized a His_6_Get5 crosslink product of approximately 40 kDa, which corresponds to the expected mass of Rpl26xHis_6_Get5 or Rpl35xHis_6_Get5, respectively (Get5: 25 kDa, Rpl26: 14 kDa, Rpl35: 14 kDa) (Fig. 2c, Rpl35-XL and Rpl26-XL).

**Fig. 3:**
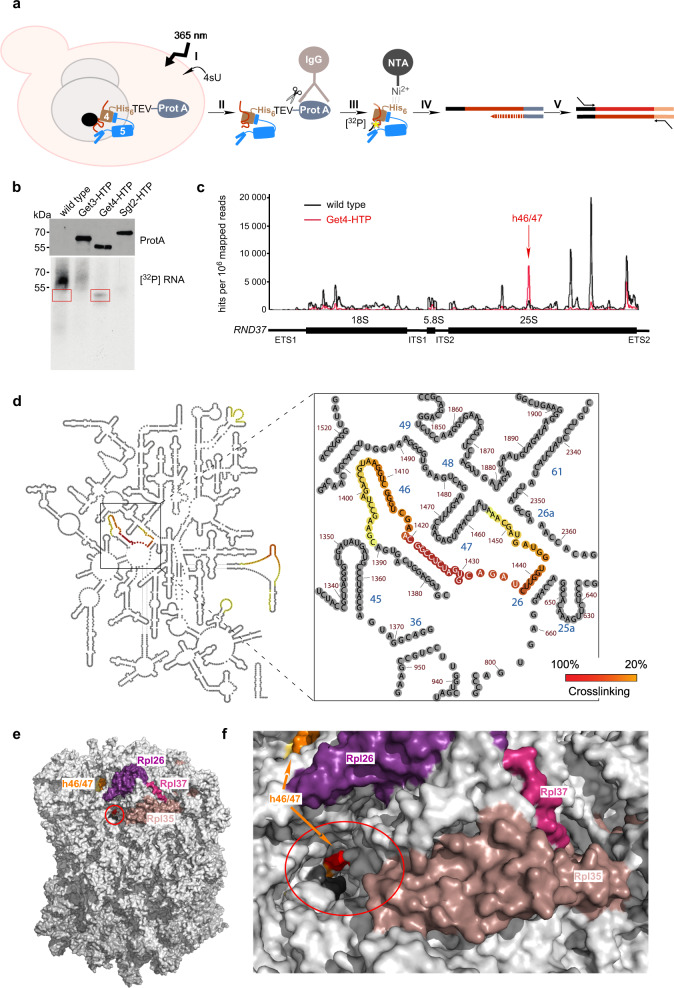
Ribosome-bound Get4 contacts h46/47 of the 25S rRNA. **a** Crosslinking and analysis of cDNA (CRAC). (I) Yeast expressing His_6_-TEV-ProtA-tagged (HTP-tagged) Get4 (Get4-HTP) was grown in the presence of 4-thiouracil (4sU) and was then crosslinked via irradiation at 365 nm. (II) Get4-HTP-containing complexes were purified under native conditions, RNA was trimmed, and TEV protease-mediated cleavage was performed. (III) RNA fragments were [^32^P]-labeled, adaptors were ligated, His_6_Get4-containing crosslink products were denatured, and purified via Ni-NTA. (IV) cDNA libraries were generated, (V) amplified by PCR, and sequenced. Yeast cell (pink), ribosome (gray), polypeptide tunnel exit (black), crosslinked rRNA (red), Get4/5 as in Fig. 2a. **b** Get4 contacts RNA. ProtA-purified, trimmed, [^32^P]-labeled, and crosslinked material (ProtA immunoblot, upper panel) and 6 h autoradiography ([^32^P]-labeled RNA, lower panel) of the indicated strains. Membrane regions, which were employed for further analysis as shown in panels **c** and **d** are boxed in red. **c** Get4 contacts 25S rRNA h46/h47. Sequencing reads mapping to nucleotides of the *S. cerevisiae RDN*37 rDNA sequence. Get4-HTP (red) and wild type (black). Shown is a representative experiment of two biological replicates (accession code GSE151664). PCR amplification (panel **a**, step V) was 35 cycles for wild type, and 24 cycles for Get4-HTP. **d** Heat map of Get4 contacts to 25S rRNA. 25S rRNA secondary structure and zoom into the rRNA region crosslinked to Get4^72^. Red (100%) indicates the maximum number of reads obtained, lower numbers of reads (>20%) are shown in shades of orange–yellow. **e** Contacts between Get4/5, ribosomal proteins, and rRNA. Surface representation of the yeast 80S ribosome (PDB 4V88^78^). 60S (light gray) and 40S (gray). Contacts of Get5 to ribosomal proteins close to the tunnel exit: Rpl26 (deep purple), Rpl35 (dirty violet), Rpl37 (hot pink). A red circle indicates the ribosomal tunnel exit. Additional contacts of Get5 are shown in Supplementary Fig. 3j and Table 1. **f** Zoom into the ribosomal tunnel. Color code as in panel **e**. The tunnel exposed β-sheet of Rpl17 is indicated in black. Contacts of Get4 to h46/h47 of the 25S rRNA are as in **d** (see Supplementary Fig. 3k).

In a parallel approach, crosslinked, Ni-NTA purified His_6_Get5 and appropriate controls (Supplementary Fig. 2c) were analyzed by mass spectrometry (see Methods, Supplementary Dataset 1, and Source Data file). From the data obtained by mass spectrometry, potential ribosomal crosslinking partners of His_6_Get5 were selected based on the following criteria: (i) the analysis was confined to core ribosomal proteins (RPs); (ii) only those RPs were selected for which the total spectral counts of His_6_Get5xRP was at least 1.4-fold increased compared to the Get5xRP control; and (iii) the *p* value was ≤0.01 (Supplementary Dataset 1). Only four large subunit ribosomal proteins (Rpl20/eL20, Rpl23/uL14, Rpl26/uL24, and Rpl37/eL37) and two small subunit ribosomal proteins (Rps19/eS19 and Rps29/uS14) met these criteria (Table 1). The mass spectrometry approach confirmed Rpl26 as a contact of ribosome-bound His_6_Get5, however, did not identify Rpl35 as a crosslinking partner of His_6_Get5. We therefore tested whether the crosslinked species recognized by α-Rpl35 (Fig. 2c) was indeed His_6_Get5xRpl35. To that end, we employed strains expressing FLAG-tagged Rpl35 (FLAGRpl35) or endogenous untagged Rpl35 in a His_6_Get5 background. After crosslinking and purification a ~40 kDa crosslink product was detected by both α-Rpl35 and α-FLAG specifically in the His_6_Get5/FLAGRpl35 strain (Fig. 2d). The combined data identify two major ribosomal contacts of Get5 as proteins Rpl26 and Rpl35. Of note, Rpl26 and Rpl35 localize adjacent to each other in the proximity of the ribosomal tunnel exit (see also Fig. 3 and Supplementary Fig. 3). The reason why the crosslink of His_6_Get5 to Rpl35 was not identified via the mass spectrometry approach remains unclear; we note that small proteins containing a large number of positively charged lysine and arginine residues are frequently difficult to identify via mass spectrometry analysis^31^(Supplementary Fig. 2d).

**Table 1:** Contacts of Get5 to ribosomal proteins identified by mass spectrometry.

Hit	MW (kDa)	Coverage (%)	*p* value	Total spectra	His_6_-Get5/Get5 (total spectra)
Rpl20a (eL20)	20.4	62	0.0001	405	1.45
Rpl23a (uL14)	14.5	43	0.0078	31	1.47
Rpl26b (uL24)	14.2	30	0.00036	23	1.44
Rpl37a (eL37)	9.9	25	0.0088	25	1.60
Rps19a (eS19)	15.9	34	0.00011	21	1.47
Rps29a/b (uS14)	6.7	70	0.0044	71	1.79
His_6_-Get5	23.7	84	0.0001	757	5.29
Get4	36.3	43	0.0001	152	3.15
Sgt2	37.2	14	0.0015	9	n.d. (0 in Get5 control)

Given are the conventional yeast names of ribosomal proteins and in brackets their names according to the unified nomenclature^77^. The *p* value was determined by a one-way ANOVA test (performed in Scaffold and followed by a Benjamini–Hochberg correction) and core ribosomal proteins with a *p* value <0.01 were selected according to their enrichment in the His-tagged sample with a 1.4-fold enrichment used as a threshold (see Supplementary Dataset 1).

### Get4 contacts helix 46/47 of the 25S ribosomal RNA

To map the binding site of Get4/5 on the ribosome in more detail, we utilized the method of crosslinking and analysis of cDNA (CRAC)^32,33^. In these experiments, proteins expressed with a His_6_-TEV-ProtA-tag (HTP-tag) are crosslinked to their associated RNAs in vivo and subsequently RNA fragments protected by the HTP-tagged protein are identified (Fig. 3a). Yeast strains expressing C-terminally HTP-tagged Get4, and as controls Get3 and Sgt2, from their genomic loci were generated (Supplementary Fig. 3a, b). For reasons unknown and despite repeated attempts, the strain expressing Get5-HTP from the *GET5* genomic locus displayed growth defects and a low Get5-HTP expression level (Supplementary Fig. 3a, b). Thus, plasmid encoded N- or C-terminally HTP-tagged Get5 constructs were expressed in a Δ*get5* strain (Supplementary Fig. 3c, d). Crosslinking of the HTP-tagged proteins to cellular RNAs revealed that only Get4-HTP was specifically crosslinked to RNA (Fig. 3b and Supplementary Fig. 3e). The membrane area containing RNAs specifically crosslinked to Get4-HTP, and the equivalent area of the wild type lane, were excised (Fig. 3b). RNAs were eluted, reverse transcribed and cDNA libraries amplified by PCR (Supplementary Fig. 3f) were subjected to next-generation sequencing. Analysis of the relative distributions of normalized, mapped sequencing reads (Supplementary Fig. 3g) derived from different classes of RNAs revealed enrichment of tRNA sequences with Get4-HTP compared with the wild type control (Supplementary Fig. 3h, upper panel and Supplementary Table 2). The relative distribution of sequencing reads derived from different tRNA isoacceptors was, however, not significantly altered between the wild type and Get4-HTP samples (Supplementary Fig. 3i). Non-specific enrichment of tRNAs with RNA-binding proteins during CRAC was previously reported and likely reflects the high abundance of tRNAs and their accessibility for crosslinking, rather than specific interactions^34^. We thus did not follow up on this observation. Analysis of the relative distributions of sequencing reads among other classes of RNA revealed an increased proportion of rRNA sequences associated with Get4-HTP compared with the wild type (Supplementary Fig. 3h, lower panel). Therefore, the numbers of sequencing reads in the wild type and Get4-HTP data sets mapping to each nucleotide of the *RDN37*, encoding the initial pre-rRNA transcript containing the 25S, 18S, and 5.8S rRNA sequences, were analyzed. This revealed that Get4-HTP, but not the wild type control, specifically crosslinked to a stretch of ~70 nucleotides encompassing helices h46 and h47 of 25S rRNA domain III (Fig. 3c, d). Thus, Get4 did not form significant crosslinks with ribosomal proteins, (Supplementary Fig. 2b), however, was in close proximity to 25S rRNA h46/h47 (Fig. 3d). These data suggest that the minor fraction of Get4, which was bound to ribosomes in the absence of Get5 (Fig. 1a, b) results from low-affinity binding of Get4 to rRNA.

In summary, five 60S ribosomal proteins were identified as crosslinking partners of Get5 (Table 1, Fig. 3e and Supplementary Fig. 3j). Three of these, Rpl26, Rpl37, and Rpl35, form a cluster, ideally positioned to provide an extended binding surface for the Get4/5 complex in close proximity of the ribosomal tunnel exit (Fig. 3f and Supplementary Fig. 3k, l). Of particular note is the Get4/5 contact to Rpl35, as eukaryotic SRP^4,35,36^, nascent polypeptide associated complex (NAC)^37,38^, the amino peptidase Map1^39^, and the Sec61 translocon^40,41^ all interact with Rpl35. Get4 contacts h46/47 of the 25S rRNA adjacent to Rpl26, which is located at the surface of the ribosome and partially within the ribosomal exit tunnel (Supplementary Fig. 3k). Additional contacts of Get5 (Rpl20, Rpl23, Rps19, and Rps29) faced the opposite side of the ribosome (Supplementary Fig. 3j) and the relevance of these contacts requires further investigation.

### Get4/5 and SRP possess overlapping ribosome-binding sites

**Fig. 4:**
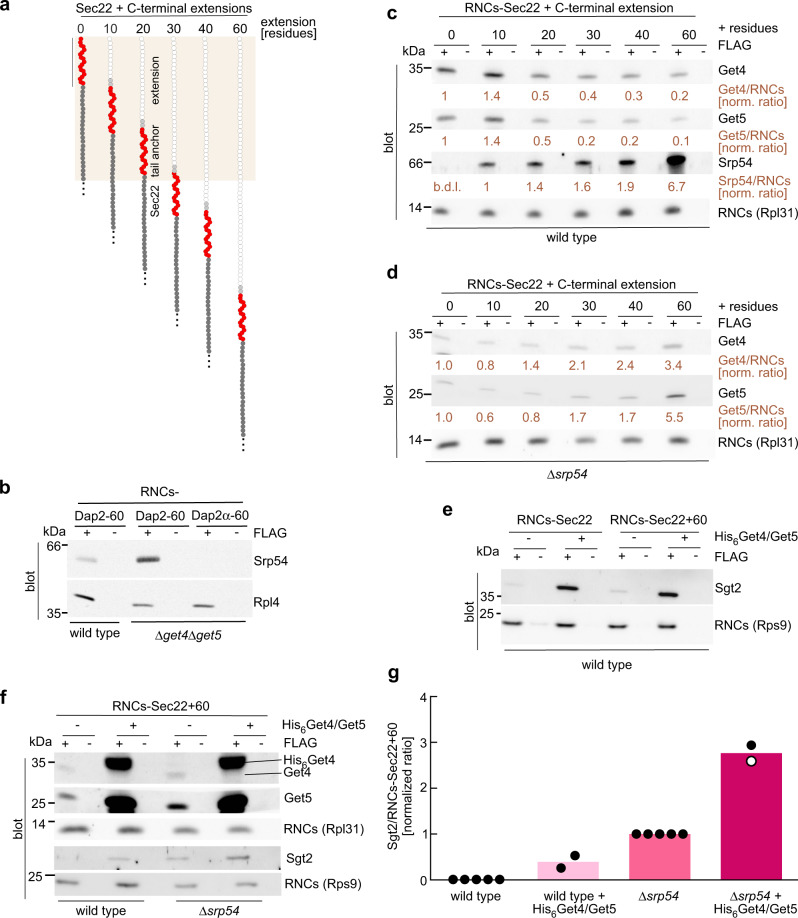
Competition between Get4/5 and SRP with respect to ribosome-binding. **a** Schematic representation of in vitro analyzed RNCs. The C-terminus of the tail-anchor (TA) protein Sec22 was extended by 10–60 residues. Estimate of the nascent chain covered by the ribosomal tunnel^43^ (beige), Sec22 (dark gray, tail-anchor in red, C-terminal extension in light gray and white; for details see Supplementary Fig. 4a). **b** SRP binding to RNCs-Dap2-60 is enhanced in the absence of Get4/5. FLAG-tag pull-down reactions (see Methods and Supplementary Fig. 4b) with FLAG-tagged (+FLAG) or non-tagged (−FLAG) Dap2-60 or Dap2α−60 (control without TM domain^18^) were generated in translation extracts as indicated (Supplementary Fig. 4f). The relative amount of RNCs (Rpl4) and bound SRP (Srp54) was assessed by immunoblotting. **c**–**d** SRP reduces binding of Get4/5 to RNCs when the TA sequence is exposed outside of the ribosomal tunnel. RNCs carrying FLAG-tagged Sec22 variants (**a**) were generated in a wild type (**c**) or Δ*srp54* translation extract (**d**). FLAG-pull-down reactions were performed as in **b** and intensities of immunostained bands were determined densitometrically. Relative ratios of Get4/Rpl31, Get5/Rpl31, and Srp54/Rpl31 were calculated and the ratio obtained for RNCs-Sec22 was set to 1; as the intensity of Srp54 on RNCs-Sec22 was below the detection limit (b.d.l.), RNCs-Sec22+10 were used for Srp54 normalization. See also Supplementary Fig. 4c. **e** Recruitment of Sgt2 to RNCs-Sec22 depends on Get4/5. FLAG-tag pull-down reactions were performed as in **b**. As indicated (+), purified His_6_Get4/Get5 (2 µM) was added prior to the translation reaction. **f** Sgt2 recruitment to RNCs-Sec22+60 is enhanced at high ribosome occupancy with Get4/5, or when SRP is absent. FLAG-tag pull-down reactions were performed and analyzed as in **b** with His_6_Get4/Get5 addition as in **e**. RNCs (Rps9 or Rpl31). **g** Quantification of Sgt2 recruitment to RNCs-Sec22+60. Ribosome-binding of Sgt2 was analyzed as in **f**. Shown are the normalized mean of five (wild type and Δ*srp54*) and two (wild type and Δ*srp54* + His_6_Get4/Get5) biological replicates (bars) and the result of each experiment (dots) (see Methods).

The findings described above (Figs. 2, 3e, f, Supplementary Fig. 3k, l) suggested that the ER-targeting factors Get4/5 and SRP^4,35,36^ have overlapping ribosome-binding sites. To test this possibility, we employed a yeast in vitro translation system, in which we generated RNCs carrying the yeast TA protein Sec22 as a nascent chain (Supplementary Fig. 4a). RNCs and ribosome-associated factors can be affinity purified via a FLAG-tag fused to the N-terminus of the nascent chain (Supplementary Fig. 4b). In order to test the effect of the emerging TA sequence, a linker of increasing length was added to Sec22 following the C-terminal TA sequence (RNCs-Sec22 + 10–60), Fig. 4a and Supplementary Fig. 4a). In a first series of experiments, RNCs carrying Sec22, Sec22 + 10, or Sec22 + 20, with the TA sequence inside of the ribosomal tunnel, were tested for the binding of Get4/5 and SRP (Supplementary Fig. 4c, d). After in vitro translation, 2–3% of ribosomes were isolated as RNCs carrying FLAG-tagged nascent chains (Supplementary Fig. 4c and^42^). When RNCs carried the most N-terminal 100 residues of the soluble protein Pgk1 as a nascent chain ~2% of Get4/5 and 0.3% of SRP were associated (Supplementary Fig. 4c). When RNCs, however, carried nascent Sec22 with the TA sequence inside of the tunnel, binding of Get4/5 was increased to 3–6% and that of SRP to 2–3% (Supplementary Fig. 4c). Binding of Get4/5 to RNCs-Sec22 or RNCs-Sec22 + 60 was independent of Sgt2 or Get3, confirming that Get4/5 was bound to RNCs carrying FLAG-tagged nascent chains (Supplementary Note 2 and Supplementary Fig. 4e, f). The combined data confirm previous findings indicating that a TA sequence/TM domain inside of the ribosomal tunnel enhances the affinity of Get4/5 and SRP for ribosomes^4,18,24^. Moreover, the side-by-side analysis of Get4/5 and SRP (Supplementary Fig. 4c) suggested that the two targeting factors display similar affinities for ribosomes containing a TA sequence within the ribosomal tunnel. To further substantiate competition between Get4/5 and SRP, we employed the SRP substrate Dap2, a type 2 membrane protein, the early targeting steps of which were characterized in detail^18,42,43^. SRP binds with low affinity to RNCs carrying the N-terminal 60 residues of Dap2 (RNCs-Dap2-60)^18^, which contain the Dap2 TM domain (residues 30-45) inside of the ribosomal tunnel (Supplementary Fig. 4g). To test whether Get4/5 affected this low-affinity binding of SRP to RNCs-Dap2-60, SRP binding was studied in the presence or absence of Get4/5 (Fig. 4b). The occupancy of RNCs-Dap2-60 with SRP was significantly increased when Get4/5 was absent from the reaction (Fig. 4b). We conclude that Get4/5 and SRP compete for ribosome binding when a TM domain is inside of the ribosomal tunnel (Supplementary Fig. 4c, Fig. 4b, and Supplementary Note 3).

The analysis was then extended to RNCs bound to Sec22 with longer C-terminal extensions (Fig. 4a). Exposure of the TA sequence outside of the ribosomal tunnel resulted in reduced binding of Get4/5, while at the same time binding of SRP was strongly increased (Fig. 4c). This observation suggests that Get4/5 cannot bind if SRP is bound to RNCs exposing a TM domain. To test this directly, RNCs were generated in an in vitro translation reaction lacking SRP (Fig. 4d and Supplementary Fig. 4f). When SRP was absent, the amount of Get4/5 bound to RNCs remained high, even when the TA sequence was accessible (Fig. 4d). We conclude that tight binding of SRP to ribosomes exposing an SRP recognition sequence disfavors ribosome-binding of Get4/5.

### Ribosome-bound Get4/5 recruits Sgt2 to facilitate TA protein transfer to Sgt2

The Get4/5 complex forms an elongated heterotetramer^11,28^, which interacts with Sgt2 via the ubiquitin-like (UBL) domain of Get5^11,44^. As Sgt2 is the GET pathway component, which first captures newly synthesized TA proteins, we asked whether ribosome-bound Get4/5 is able to interact with Sgt2 and by that, positions Sgt2 in close proximity of the tunnel exit (Supplementary Fig. 4h). FLAG-tag pull-down experiments with RNCs-Sec22 (TA sequence within the ribosomal tunnel) revealed that the amount of ribosome-associated Sgt2 was close to the detection limit of the Sgt2 antibody, and the result remained ambiguous (Fig. 4e, lane 1 and 2). We reasoned that if Get4/5 indeed recruited Sgt2, a higher occupancy of RNCs with Get4/5 should result in enhanced recruitment. To test this possibility, purified His_6_Get4/Get5 was added to pull-down reactions (Fig. 4e and Supplementary Fig. 4i, j). Indeed, when the occupancy of RNCs with Get4/5 was high, significantly more Sgt2 was associated with RNCs-Sec22, even though the concentration of Sgt2 in the reaction remained unaltered (Fig. 4e, lanes 3 and 4). A similar increase in Sgt2 recruitment was observed when purified His_6_Get4/Get5 was added to RNCs-Sec22 + 60, which expose the TA sequence outside of the ribosomal tunnel (Fig. 4e, lanes 5–8). Binding of Get4/5, as well as Get4/5-dependent binding of Sgt2, was also tested with RNCs carrying Sed5 or Bos1 (Supplementary Fig. 4k). RNCs-Sed5 + 60 or -Bos1 + 60 recruited Get4/5 and a low amount of Sgt2, close to the detection limit of the Sgt2 antibody, in a wild type translation extract (Supplementary Fig. 4k). When His_6_Get4/Get5 was added to the reaction, binding of Sgt2 was strongly enhanced (Supplementary Fig. 4k). Consistent with enhanced association of Get4/5 with RNCs-Sec22 + 60 in the absence of SRP (Fig. 4d, f), Sgt2 recruitment to RNCs-Sec22 + 60 was increased in the absence of SRP, and was further stimulated about 3-fold when purified His_6_Get4/Get5 was added to the reaction (Fig. 4f, g).

**Fig. 5:**
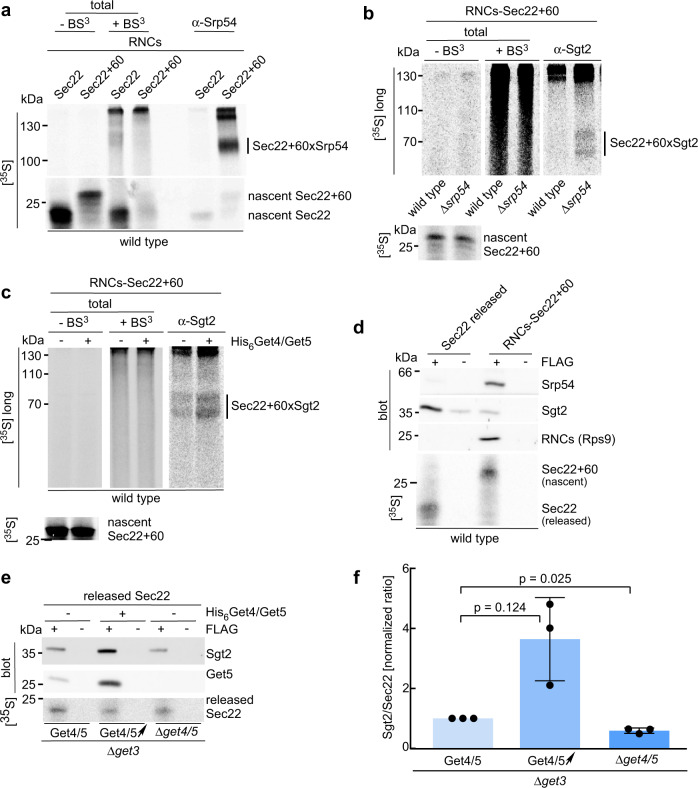
Ribosome-bound Sgt2 is in contact with a nascent TA sequence. **a** SRP contacts the TA sequence of nascent Sec22+60 exposed outside of the ribosomal tunnel. RNCs carrying radiolabeled nascent Sec22 or Sec22+60 were crosslinked with BS^3^ and SRP was affinity purified with α-Srp54. Total (5%) translation reactions before (−BS^3^) or after (+BS^3^) crosslinking. See Supplementary Fig. 5a and Methods. Shown is an autoradiograph; Sec22+60xSrp54 represents the crosslink product between Srp54 and nascent Sec22+60. **b** Sgt2 contacts nascent Sec22+60 when SRP is absent from the reaction. Radiolabeled RNCs-Sec22+60 generated in wild type or Δ*srp54* translation extracts were analyzed as detailed in **a** using α-Sgt2 for affinity purification of crosslink products. Sec22+60xSgt2 represents crosslink products between Sgt2 and nascent Sec22+60. **c** Sgt2 contacts nascent Sec22+60 when RNCs are highly occupied with Get4/5. Radiolabeled RNCs-Sec22+60 were analyzed with or without purified His_6_Get4/Get5 (2 µM) (Supplementary Fig. 4i). **d** Ribosome-released Sec22 is mainly bound to Sgt2, whereas nascent Sec22+60 is mainly bound to SRP. FLAG-tag pull-down reactions were performed with radiolabeled FLAG-tagged Sec22 and RNCs-Sec22+60 (Supplementary Fig. 4b and Supplementary Fig. 5f). Samples containing similar amounts of released Sec22 or RNCs-Sec22+60 were analyzed for binding of SRP (Srp54) or Sgt2 via immunoblotting. α-Rps9 was employed to detect RNCs. **e** Ribosome-bound Get4/5 improves the capture of released Sec22 by Sgt2. Radiolabeled released Sec22 was generated in Δ*get3* or Δ*get3*Δ*get4*Δ*get5* translation extracts (Supplementary Fig. 4f). When indicated (+), purified His_6_Get4/Get5 (2 µM) was added prior to the translation reaction. FLAG-tag pull-down reactions were performed and equal amounts of affinity purified, released Sec22 were analyzed for Sgt2 and Get4/5 (Get5). **f** Quantification of the capture of released Sec22 by Sgt2. The relative efficiency with which Sgt2 was bound to released Sec22 (**e**) was determined (see Methods). Shown is the mean of three independent experiments (bars), with the standard deviation, and the result of each experiment (dots). *p* values were calculated by one-way ANOVA with GraphPad Prism.

Proximity of nascent Sec22 (with TA inside of the ribosomal tunnel) and Sec22 + 60 (with TA outside of the ribosomal tunnel) to SRP, Get4/5, and Sgt2 was further analyzed by crosslinking (Supplementary Fig. 5a). SRP did not contact nascent Sec22, however, was in direct contact with nascent Sec22 + 60 (Fig. 5a, see Fig. 4c for occupancy of RNCs-Sec22/Sec22 + 60 with SRP). Thus, a bona fide TA sequence served as an SRP recognition sequence when it was exposed as a nascent chain outside of the ribosomal tunnel. We did not detect crosslinks between Get4 or Get5 and nascent Sec22 + 60 (Supplementary Fig. 5b, c), or between Sgt2 and nascent Sec22, even when the occupancy of RNCs-Sec22 with Get4/5 and Sgt2 was high (Supplementary Fig. 5d, see Fig. 4e for occupancy of RNCs-Sec22 with Sgt2). However, when ribosomes carried nascent Sec22 + 60, and thus the TA sequence was exposed, Sgt2 was crosslinked to the nascent chain (Fig. 5b, c). In a wild type translation extract, crosslinking between Sgt2 and nascent Sec22 + 60 was weak, in some experiments barely above the detection limit (Fig. 5b, c). Crosslinking between Sgt2 and nascent Sec22 + 60 was significantly increased when SRP was absent (Fig. 5b) or when the occupancy of RNCs-Sec22 + 60 with Get4/5 was high (Fig. 5c). Thus, the extent of crosslinking between Sgt2 and nascent Sec22 + 60 correlated with the occupancy of RNCs-Sec22 + 60 with Get4/5/Sgt2 (Fig. 4e, f). The ribosome binding and crosslinking data shown in Figs. 4 and 5 are consistent with the well-characterized properties of SRP, which efficiently interacts with nascent TM domains, and Get4/5, which does not interact with TA sequences directly^1,5,15,16^. In addition, the data reveal that Sgt2 was recruited to RNCs via Get4/5 even before the TA sequence emerged from the tunnel (Fig. 4e), but moved into close proximity to the nascent TA protein only after the TA sequence had emerged from the ribosomal tunnel (Fig. 5b, c).

In vivo, TA sequences do not exit the ribosomal tunnel during ongoing translation, and emerge only after translation termination (Supplementary Fig. 5e). To investigate TA recognition during peptide release, we produced a ribosome-released TA protein by programming the yeast translation extract with an mRNA encoding full-length Sec22 including the stop codon (Supplementary Fig. 5f). We first assessed how much Sgt2 or SRP was bound to released Sec22 compared with nascent Sec22 + 60. To control for equal loading, released Sec22 and nascent Sec22 + 60 were labeled with [^35^S]-methionine (Fig. 5d). Consistent with previous data^1,5,15,16^, Sec22 released upon translation was mainly bound to Sgt2 (Fig. 5d and Supplementary Fig. 5g and h), whereas nascent Sec22 + 60 was mainly associated with SRP (Fig. 5d). These data raised the question of whether the Get4/5-dependent recruitment of Sgt2 to ribosomes (Fig. 4) facilitated the capture of released Sec22 by Sgt2. As Get4/5 has an additional role during a later step of the GET pathway when the TA protein is transferred from Sgt2 to Get3, the analysis was performed in the absence of Get3^45^. To that end, capture of Sec22 by Sgt2 was analyzed in a Δ*get3* translation extract with or without addition of purified His_6_Get4/5, and in a Δ*get3*Δ*get4*Δ*get5* translation extract (Fig. 5e and Supplementary Fig. 4f). In this setup, addition of purified His_6_Get4/Get5 consistently enhanced binding of Sec22 to Sgt2 by more than twofold (Fig. 5e, f). Moreover, a smaller fraction of Sec22 was bound to Sgt2 when Get4/5 was absent from the reaction (Fig. 5e, f). We conclude that Get4/5 is not only required for the transfer of TA proteins from Sgt2 to Get3^1,5,10,15,16,45^, but prior to that, Get4/5 facilitates efficient transfer of TA proteins to Sgt2 upon ribosome-release.

### In vivo binding of Sgt2 to ribosomes depends on Get4/5

**Fig. 6:**
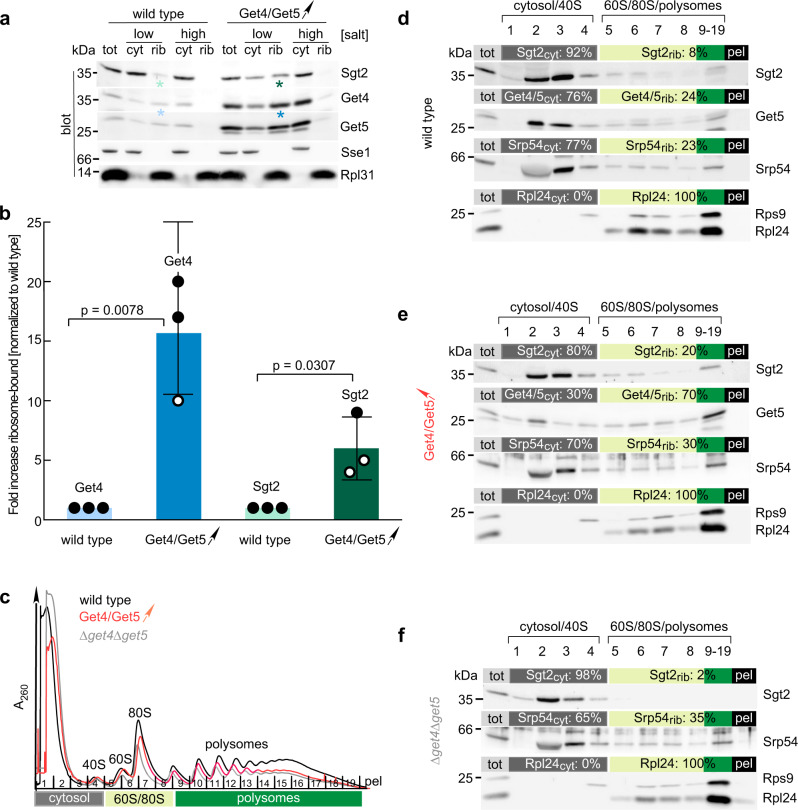
Sgt2 is recruited to translating ribosomes via Get4/5 in vivo. **a** Sgt2 is associated with ribosomes in a Get4/5-dependent manner. Total cell extract (tot) of wild type or a strain overexpressing Get4/5 (Get4/Get5↑) was analyzed as described in Fig. 1a. Aliquots were analyzed by immunoblotting with α-Get4, α-Get5, α-Sgt2, α-Rpl31 (ribosomal marker), and α-Sse1 (cytosolic marker). Colored asterisks indicate the bands analyzed in **b**. **b** Ribosome occupancy with Sgt2 is enhanced upon overexpression of Get4/5. Ribosome-binding of Get4 and Sgt2 under low salt conditions was analyzed as in **a**. Shown is the mean (bars with standard deviation) of three independent experiments (dots). Values obtained for the wild type were set to 1. Indicated *p* values for Get4 or Sgt2 were calculated according to a two-sided Student’s *t* test using GraphPad Prism. The color code of the bars corresponds to the asterisks in **a**. **c** Ribosome profile analysis reveals a sub-population of Sgt2 in polysome fractions, which depends on the presence of Get4/5. Sucrose density gradient centrifugation was performed with total cytosolic extract prepared from wild type (black), cells overexpressing Get4/Get5 (Get4/Get5↑) (red), or Δ*get4*Δ*get5* cells (light gray). Indicated in dark gray are the major cytosolic fractions and 40S particles (1–4), in light green (5–8) fractions containing 60S and 80S ribosomal particles, and dark green (9–19) polysome fractions. **d**–**f** Aliquots of individual fractions 1–8, pooled fractions 9–19, and the pellet fraction at the bottom of the gradient tube (pel) (see **c**) were analyzed via immunoblotting with α-Sgt2, α-Get5, α-Srp54, the 40S subunit (α-Rps9), and the 60S subunit (α-Rpl24) (for details see Methods). The total (tot) corresponds to 5% of the cytosolic extract loaded onto the gradient. The distribution of ribosome-bound factors between cytosol and ribosomes was estimated as follows. Intensities of immunostained bands in fractions 1–19 (100% = total intensity of fractions 1–19) was determined for each ribosome-bound factor. The percentage of each ribosome-bound factor in the cytosolic fractions 1–4 (cyt: %) and in the ribosomal fractions 5–19 (rib: %) was calculated relative to the total. The color code is as in **c**.

The above findings suggested that in living cells, a fraction of Sgt2 could be ribosome-bound. Previous analysis failed, however, to provide evidence for a ribosome-bound pool of Sgt2^24^. Based on the above data, we revisited the question of ribosome-binding of Sgt2 in vivo. For that purpose, we increased sample loading and further optimized immunoblotting with the highly specific, but poorly sensitive Sgt2 antibody (Fig. 6a). A minor portion of Sgt2 was detected in ribosomal fractions of a wild type cell extract under low salt conditions and was released by high salt treatment (Fig. 6a). Moreover, when Get4/5 was overexpressed the fraction of ribosome-bound Sgt2 was increased (Fig. 6a, b). Ribosome profile analysis in the wild type strain, a strain overexpressing Get4/5, and a Δ*get4*Δ*get5* strain was conducted to further validate Get4/5-dependent binding of Sgt2 to translating ribosomes (Fig. 6c). To enhance detection of factors bound to polysomes, a modified ribosome profile protocol, which combines the polysome fractions prior to analysis was developed (see Methods). In a total extract derived from wild type a small fraction of Sgt2 co-migrated with ribosomes (Fig. 6d, fractions 5–19), in the extract from Get4/5 overexpressing cells the fraction of Sgt2 in ribosome fractions was increased more than twofold (Fig. 6e, fractions 5–19), and when Get4/5 was absent, the amount of Sgt2 associated with ribosomes was strongly reduced (Fig. 6f, fractions 5–19). The fraction of SRP associated with ribosomes was largely unaffected by the level of Get4/5 (Fig. 6d, f, fractions 5–19); this finding is in line with the observation of Get4/5-independent binding of SRP to the ribosome. Increasing the expression level of Get4/5 resulted in a significantly higher occupancy of ribosomes with Get4/5, which in turn resulted in a higher occupancy of ribosomes with Sgt2 (Fig. 6a and d-f). Together, these data support the requirement of Get4/5 for ribosome recruitment of Sgt2.

## Discussion

In this work, we show how newly synthesized tail-anchored proteins are captured upon their emergence from the ribosomal tunnel. Five major conclusions can be drawn from the results of this study: (i) Get4/5 binds directly to the ribosome via the Get5 subunit; (ii) Get4/5 and SRP have overlapping binding sites and compete with each other for binding to the ribosome; (iii) ribosome-bound Get4/5 recruits Sgt2; (iv) ribosome-associated Sgt2 contacts exposed TA sequences; and (v) Get4/5-dependent ribosome recruitment of Sgt2 improves capture of TA proteins released to the cytosol upon translation termination.

### Get5 mediates binding of Get4/5 to the ribosome

Get5 has a modular architecture, which allows it to interact with different partner proteins. The N-terminal domain of Get5 stably binds to its partner Get4, whereas the adjacent UBL domain provides the binding site for the interaction with Sgt2, and the C-terminal domain is responsible for self-assembly into a stable homodimer^11,46^. Our analysis reveals that, in addition, Get5 contacts ribosomal proteins Rpl26 and Rpl35 close to the ribosomal tunnel exit. Which domain of Get5 mediates binding to the ribosome is currently unknown. We consider it unlikely that ribosome binding involves the N-terminal or the UBL domains, because ribosome-bound Get5 is in a complex with Get4 and can interact with Sgt2. A possible candidate is the C-terminal homodimerization domain, the function of which is presently not understood^11^. As the C-terminal domain of Get5 is negatively charged, it seems possible that electrostatic interactions mediate binding to positively charged patches within its ribosomal contact site (Supplementary Fig. 3l). Interestingly, the homodimerization domain of Get5 is not conserved in the mammalian Get5 homolog UBL4A^11^. This would be consistent with the observation that mammalian UBL4A does not directly bind to ribosomes^19^ (see also below).

### Ribosome binding of Get4/5 versus SRP: competition modulated by the emerging nascent chain

The amounts of SRP (~8000 molecules per cell^42,47^) and Get4_2_Get5_2_ (~5000 molecules per cell^47^) in living yeast cells are similar. Both proteins are much less abundant than cytosolic ribosomes, which are present at ~300,000 molecules per yeast cell^42^. This stoichiometry suggests that Get4/5 has to scan ribosomes in order to identify those carrying cognate nascent substrates, as it is well documented for SRP^4,21,48^. SRP and Get4/5 display surprisingly similar affinities for vacant ribosomes, with a *K*_d_ of 110 ± 40 nM for Get4/5 (this study) and 71 ± 36 nM^49^ or 120 ± 29 nM^50^ for SRP. Binding of Get4/5 and SRP to the ribosome is enhanced when a nascent TA sequence/TM domain enters the exit tunnel (^4,18,24^ and this study). Furthermore, the affinities of Get4/5 and SRP for translating ribosomes with a TA sequence/TM domain inside the exit tunnel resemble each other. These binding properties allow the two targeting factors to freely bind and dissociate from ribosomes in dynamic cycles and scan for TM domains emerging inside of the tunnel. Once a nascent TM domain becomes exposed outside of the tunnel, SRP binds to it with >1000-fold higher affinity^49^, whereas the affinity of Get4/5 remains largely unaffected (this study, see Supplementary Note 3).

### Get4/5 functions as an adaptor of Sgt2 at the tunnel exit

**Fig. 7:**
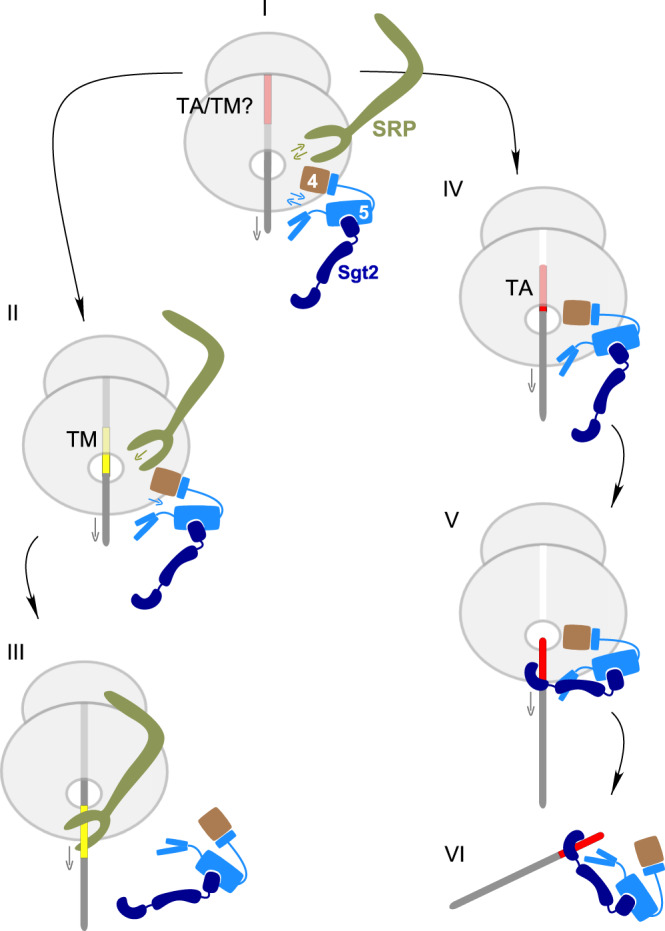
Model of TM domain capture upon exit from the ribosomal tunnel. (I) A sequence with the characteristics of a hydrophobic α-helical transmembrane domain emerges from the peptidyl transferase center and enters the ribosomal tunnel. At that point, SRP and Get4/5 cycle on and off ribosomes, as it remains unclear if the hydrophobic sequence is a TM domain (internal transmembrane domain) or a TA sequence (C-terminal transmembrane domain). (II) In case translation continues, the hydrophobic sequence remains ribosome-bound as part of the nascent chain and becomes an internal TM domain. (III) Upon exit from the tunnel, the internal TM domain is recognized by SRP. As SRP now binds with high affinity, the binding and release cycle of Get4/5, and concurrently Sgt2, is interrupted. (IV) If translation termination occurs immediately after synthesis of the hydrophobic domain, which is then termed TA sequence, SRP is not efficiently recruited to the now non-translating ribosome or the released TA sequence. (V) In this case, Get4/5, and via Get4/5 also Sgt2, continue to sample ribosomes and Sgt2 can capture the TA sequence directly upon arrival at the tunnel exit. Ribosome (light gray), nascent chain (dark gray) TA sequence (red), TM domain (yellow), Get4 (brown) Get5 (light blue), Sgt2 (dark blue), SRP (green). TA (C-terminal tail-anchor sequence) and TM (internal transmembrane domain).

Our findings reveal that Get4/5 does not only scan ribosomes but also recruits Sgt2 into close proximity of the tunnel exit. At this earliest stage of membrane protein synthesis, both Get4/5 and SRP sample ribosomes in dynamic equilibrium (Fig. 7 stage I). Depending on the type of membrane protein, either translation continues after synthesis of the TM domain, or translation termination occurs. If translation continues, an internal TM domain emerges from the tunnel, to which SRP binds with high affinity, thereby excluding Get4/5 from scanning the ribosome (Fig. 7 stage II and III). If translation termination occurs, the TM domain turns into a TA sequence, to which SRP does not bind with high affinity, Get4/5 scanning continues (Fig. 7 stage IV), and allows Sgt2, poised at the tunnel exit, to capture the released TA sequence (Fig. 7 stage V–VI).

### Transfer of TA proteins to Sgt2 after release from the ribosome

Sgt2 contains a tetratricopeptide repeat domain, which interacts with cytosolic chaperones, including the Hsp70 homolog Ssa^10,51,52^. It was recently shown that the interaction of Sgt2 with Ssa is required for the efficient capture of TA proteins by Sgt2^45^. Of note, the experimental setup of that study is significantly different from ours, as it employed either purified TA proteins, or newly synthesized TA proteins generated in an *Escherichia coli* translation system supplemented with yeast GET components and chaperones^45^. As the GET pathway is not conserved in the bacterial system, we consider it unlikely that Get4/5/Sgt2 binds to the *E. coli* ribosome. This may explain the strong tendency of the TA protein to aggregate even when Get4/5 and Sgt2 were added to the *E. coli* translation reaction. Vice versa, even though Get4/5 significantly enhanced Sgt2-binding to a released TA protein in the yeast translation system, Get4/5 was not strictly required for the capture in our experimental system. Based on the data from the Shan lab^45^ and our observations, we suggest that Ssa may facilitate the Get4/5-independent, posttranslational capture of TA proteins by Sgt2 in the yeast translation system, which contains Ssa and other cytosolic chaperones in high concentrations.

### The mammalian system: similarities and differences between Get4/5/Sgt2 and BAG6/SGTA

Mammalian cells express a complex consisting of GET4 (Get4 homolog), UBL4A (Get5 homolog), and BAG6 (no yeast homolog identified), which is termed the BAG6 complex^1,11,14^. In contrast to Get5, UBL4A does not homodimerize and does not directly bind to GET4. Instead, UBL4A and GET4 bind to BAG6 independently of each other^1,11,14,53^. Depletion of BAG6 results in defective TA protein capture by GET3 (Get3 homolog)^19^. Indeed, the BAG6 complex mediates the transfer of TA proteins from SGTA (Sgt2 homolog) to GET3^53,54^ and thus, even though structurally different, functionally resembles the yeast Get4/5 complex^10^. In contrast to Get4/5, however, substrate proteins can interact directly with the BAG6 complex via its BAG6 subunit^11,19,55,56^. Moreover, BAG6 recruits the ubiquitin ligase RNF126, which polyubiquitinates proteins bound to BAG6 inducing subsequent degradation by the proteasome^11,14,56^. Owing to its physical interaction with GET pathway components, as well as the proteolytic system, BAG6 is involved in the triage of newly synthesized proteins between biosynthesis and degradation^11,14^. Although the latter function is not conserved in yeast, the BAG6 complex is recruited to ribosomes via the BAG6 subunit and in this respect resembles the yeast homolog Get4/5^19^. The function of ribosome-bound BAG6 in mammalian cells is currently unclear. Based on the observations in this study, one could envisage that SGTA is recruited to the BAG6 complex via the UBL4A subunit. However, a recent finding reveals that this is not the case, even though SGTA binds to the mammalian ribosome^57^. Seemingly, in the mammalian system the interaction between SGTA and the ribosome is direct and independent of the BAG6 complex^57^. Similar to Sgt2, however, SGTA interacts with TM domains emerging from the ribosomal tunnel and shields them from off-pathway reactions^57^. Thus, although yeast and mammals employ different mechanisms to anchor the Get4/5/BAG6 complex and Sgt2/SGTA to ribosomes, the function of the ribosome-bound GET components in capturing TA proteins appears conserved.

## Methods

### Yeast strains and plasmids

Yeast strains and plasmids are listed in Supplementary Table 3. BY4741^58^ and MH272-3fα^59^ represent the wild type strains for all mutant strains employed in this study. The FLAGRpl35 strain was previously described^60^. Δ*get3*Δ*get4*Δ*get5* was generated by mating of Δ*get3*α with Δ*get4*Δ*get5*a^24^ followed by sporulation and tetrad dissection. The Δ*get3*Δ*sgt2* strain was generated by introducing a *sgt2*::*URA3* deletion cassette^24^ into the Δ*get3* strain. Yeast genomic DNA from MH272-3fα or BY4741 was used for PCR amplification of *SGT2, GET4*, and *GET5*, for the construction of *E. coli* and yeast expression vectors. Yeast shuttle vectors for the overexpression of Get4, Get5, N-terminally His_6_-tagged His_6_Get4, and His_6_-tagged His_6_Get5 are based on the pYEP and pRS series^61,62^. pYEplac181-His_6_-Get5 was constructed by replacing the Get5 *orf* in pYEplac181-Get5 with His_6_Get5, which was generated by PCR using synthetic DNA (Bio Cat) as a template (Supplementary Table 4). For in vitro translation experiments Sec22 + 60 was introduced into pSP65 (Addgene) using the EcoRI/XbaI sites. Sec22 + 60 consists of full-length Sec22, which was C-terminally fused to residues 2–61 of yeast Pgk1 via *Xba*I/*Hin*dIII, which introduces a two-residue linker (SR) to the C-terminus of Sec22 (Supplementary Fig. 4a). In vitro translation vectors encoding for FLAG-Sed5 + 60/Sed5 + 60 and FLAG-Bos1 + 60/Bos1 + 60 were constructed in pSP65. FLAG-Sed5 + 60/Sed5 + 60 were constructed by replacing Sec22 in pSP65-Sec22 + 60/pSP65-FLAG-with Sed5 using the EcoRI/XbaI sites. Sed5 + 60 consists of full-length Sed5, a two-residue linker (SR), and residues 2–61 of yeast Pgk1 (Source Data file). FLAG-Bos1 + 60/Bos1 + 60 were generated by PCR from a synthetic DNA template (Eurofins Genomics) (Supplementary Table 4) encoding for FLAG-Bos1 + 60. The PCR products were cloned into the SacI/HindIII site of pSP65. Bos1 + 60 consists of full-length Bos1, a two-residue linker (LQ), and residues 2–61 of yeast Pgk1 (Source Data file). Plasmids for the in vitro translation of Dap2-60 and Dap2α-60 (Source Data file) were previously described^18^. For the expression of His_6_Get4/Get5 in *E. coli* the *GET4*-coding sequence was introduced into the NdeI/EcoRI sites of pET28a (pET28a-His_6_Get4) and the *GET5*-coding sequence was introduced into the NsiI/PacI sites of pETcoco2 (pETcoco2-Get5). For the expression Get4/His_6_Get5, the *GET4* coding sequence was inserted into the *Bgl*II/*Nde*I sites and the *GET5* coding sequence was inserted into the SalI/NotI sites of the pETDuet vector (pETDuet-His_6_Get5-Get4). Yeast strains expressing Get3-HTP, Get4-HTP, Sgt2-HTP, or Get5-HTP from their genomic loci were generated by homologous recombination of an HTP cassette^33^. For the plasmid-based expression of HTP-Get5 or Get5-HTP, the *GET5*-coding sequence was introduced into pRS415-based plasmids for expression of N- or C-terminally HTP-tagged proteins via the *Bam*HI/*Hin*dIII and *Xba*I/*Bam*HI sites, respectively^63^. The Get5-HTP plasmid contains a (Gly)_4_-Ser linker between the *GET5*-coding sequence and the HTP-tag. Plasmids were introduced into a Δ*get5* background and a wild type BY4741 strain carrying the empty pRS415 plasmid (wild type_e.p._) was prepared as a control (Supplementary Table 3). Primers employed in the course of the study are listed in Supplementary Table 4.

### Heterologous expression and purification of GET pathway components from *E. coli*

pET28a-His_6_-Get4 and pETcoco2-Get5 were transformed into *E. coli* BL21(DE3) and expression was induced at 25 °C with 2 mM isopropyl-β-d-1-thiogalactopyranoside. The His_6_Get4/Get5 complex was then purified using Ni-NTA super flow beads according to the manufacturer’s manual under native conditions (QIAGEN) (Supplementary Fig. 4i). Purified His_6_Get4/Get5 was transferred to Hepes buffer (20 mM Hepes/KOH pH 7.4, 2 mM Mg(OAc)_2_, 120 mM KOAc, 5% glycerol, 1 mM phenylmethylsulfonyl fluoride (PMSF)) using PD-10 desalting columns (GE Healthcare) and was then employed in the experiments shown in Figs. 4 and 5. Get4/His_6_Get5 was expressed in *E. coli* BL21(DE3) from plasmid pETDuet-His_6_Get5-Get4. Purification of Get4/His_6_Get5 of was performed using HisTrap HP affinity chromatography (GE Healthcare) followed by size-exclusion chromatography (Superdex 200 10/300 GE Healthcare). Purified Get4/His_6_Get5 was transferred to 50 mM Tris/HCl pH 7.5, 70 mM NH_4_Cl, 30 mM KCl, 7 mM MgCl_2_ buffer using PD-10 desalting columns (GE Healthcare) prior to fluorescent labeling for anisotropy and FCS measurements shown in Fig. 1. Purified proteins were stored at −80 °C.

### Preparation of 80S ribosomes

80S ribosomes and ribosomal subunits were purified from the *Saccharomyces cerevisiae* strain YAS-2488 as described previously^64,65^. Cells were harvested in mid-log phase and resuspended in 1 ml g^−1^ of cells in lysis buffer (20 mM Hepes/KOH pH 7.5, 100 mM KOAc, 2.5 mM Mg(OAc)_2_, 1 mg ml^−1^ heparin sodium salt, 2 mM dithiothreitol (DTT)). Cell pellets frozen in liquid nitrogen were ground using an ultra-centrifugal mill according to the CryoMill protocol (Retsch). The lysate was thawed at 4 °C, 100 µl DNase and one ethylenediaminetetraacetic acid (EDTA)-free protease inhibitor tablet (Roche) was added and incubated at 4 °C for 30 min. The thawed lysate was clarified by centrifugation at 25,000 × *g* in a JLA 16.250 (Beckman Coulter) at 4 °C for 30 min. The salt concentration of the supernatant was increased to 500 mM KCl and was then filtered using 1 µm glass fiber filters (Pall Corporation). Ribosomes in the supernatant were collected through a 1 M sucrose cushion (20 mM Hepes/KOH pH 7.5, 100 mM KOAc, 400 mM KCl, 2.5 mM Mg(OAc)_2_, 1 M sucrose, 2 mM DTT) by centrifugation at 235,000 × *g* at 4 °C for 2 h in a Ti45 rotor (Beckman Coulter). Ribosomal pellets were resuspended in resuspension buffer (20 mM Hepes/KOH pH 7.5, 100 mM KOAc, 400 mM KCl, 2.5 mM Mg(OAc)_2_, 1 mg ml^−1^ heparin sodium salt, 2 mM DTT) and were incubated on ice for 15 min. Ribosomes were collected once more through a 1 M sucrose cushion (20 mM Hepes/KOH pH 7.5, 100 mM KOAc, 400 mM KCl, 2.5 mM Mg(OAc)_2_, 1 M sucrose, 2 mM DTT) at 600,000 × *g* at 4 °C for 30 min in a MLA 130 rotor (Beckman Coulter). Ribosomal pellets were then resuspended in storage buffer (50 mM Hepes/KOH, 100 mM KCl, 250 mM sucrose, 2.5 mM MgCl_2_, 2 mM DTT) at a final concentration of ~10 µM and stored at −80 °C after being flash frozen in liquid nitrogen.

### Fluorescent labeling and fluorescence measurements

Get4/5-Atto655 or Get4/5-Atto390 was generated by fluorescence-labeling purified Get4/His_6_Get5 with a fivefold excess thiol reactive ATTO-655- or ATTO-390-maleimide dye (ATTO-TEC) targeting the surface-exposed cysteine of two cysteines present in Get4 (C177, 255). Note that Get5 does not contain cysteine residues. The reaction was incubated at 20 °C for 2 h and was then quenched with 1 mM DTT. Fluorescence labeled protein was purified from free dye using gel filtration chromatography (Superdex 75). Anisotropy measurements were performed at 20 °C using fluorescence labeled Get4/5-Atto390 in a FluoroMax spectrofluorometer (Horiba Scientific). Samples were excited at 380 nm and the emission at 470 nm was measured. Titrations were performed in a 150 µl cuvette in buffer (50 mM Tris/HCl pH 7.5, 70 mM NH_4_Cl, 30 mM KCl, 7 mM MgCl_2_), the concentration of Get4/5-Atto390 was held constant (20 nM) and increasing concentrations of ribosomes were added from a working stock of 1 µM to final concentrations as indicated (20–500 nM). Anisotropy (*r*) was determined from Eq. 1:

1 $$r = \frac{{I_{VV} - G * I_{VH}}}{{I_{VV} + 2 * G * I_{VH}}}$$

where *I*_*VV*_ are the light intensities with excitation and emission polarizers in the vertical direction and *I*_*VH*_ uses a vertical excitation polarizer and a horizontal emission polarizer. *G* is the G factor measured by Eq. 2:

2 $$G = \frac{{I_{HV}}}{{I_{HH}}}$$

where *I*_*HV*_ are the light intensities with a horizontal excitation polarizer and a vertical emission polarizer and *I*_*HH*_ are the light intensities with horizontal excitation and emission polarizers.

Anisotropy measurements were plotted as a function of increasing ribosomal concentration and fit with Eq. 3.

3 $$A = \frac{{A_\infty * [Rb]}}{{K_d + [Rb]}} + A_0$$

where *A*_∞_ is the anisotropy at saturating concentrations of ribosomes [Rb], *K*_d_ is the equilibrium dissociation constant of the complex, and *A*_0_ is the anisotropy of the free protein.

### Fluorescence correlation spectroscopy

Confocal FCS measurements were performed on a PicoQuant MicroTime 200 inverse time-resolved confocal microscope. We setup the experiment in a volume of 50 µl buffer (50 mM Tris/HCl pH 7.5, 70 mM NH_4_Cl, 30 mM KCl, 7 mM MgCl_2_). The concentration of Get4/5-Atto655 was 5 nM. One to two fluorophore molecules were excited using a 635 nm continuous wave laser at ~80 μW. This signal was further filtered using two 690 nm filters and the cross-correlation of the emitted light was collected. Autocorrelation functions were measured using SymPhoTime 64 software where 314 sample points were collected over a 60 sec time period in the presence and absence of 80S ribosomes (1 μM; diluted from a ca. 10 µM stock in ribosomal storage buffer) and additional unlabeled GET proteins (1 μM final concentration). A minimum of six replicates were performed to produce one average trace and subsequently fit with a two-dimensional diffusion model^66^.

4 $$G\left( t \right) = {\sum} {\frac{\rho }{{\left[ {1 + \left[ {\frac{t}{{\tau _{Diff[i]}}}} \right]} \right]^{\left[ i \right]}}} + G_\infty }$$

*G(t)* is the autocorrelation function and *G*_*∞*_ is the autocorrelation function at infinity. *T*_Diff_ is the diffusion time of the sample and *ρ* is the contribution of the ith diffusing species. After fitting, *T*_Diff_ was determined using Eq. 4 and expressed including the standard error of the parameter obtained from the confidence of the fit. Diffusion coefficients (*D*) were determined using Eq. 5.

5 $$D = \frac{{2 * V_{EFF}}}{{4 * \tau _{Diff} * K * \pi }}$$

where *V*_*Eff*_ is the effective excitation volume and *K* (kappa) is the length to diameter ratio of the focal volume, both determined from the calibration using free ATTO-655 dye^67^. All measurements were performed at room temperature.

### Ribosome-binding assay

Yeast strains were grown to early log phase on YPD, cycloheximide was added to a final concentration of 100 µg ml^−1^ and the cultures were chilled on ice. Cells were harvested by centrifugation at 5000 × *g*. Cell pellets were resuspended in ribosome-binding buffer (20 mM Hepes/KOH pH 7.4, 2 mM Mg(OAc)_2_, 120 mM KOAc, 50 µg ml^−1^ cycloheximide, 2 mM DTT, 1 mM PMSF, protease inhibitor mix: 1.25 µg ml^−1^ leupeptin, 0.75 µg ml^−1^ antipain, 0.25 µg ml^−1^ chymostatin, 0.25 µg ml^−1^ elastinal, 5 µg ml^−1^ pepstatin A) and total cell extracts were prepared by the glass beads method^68^. After a clearing spin at 20,000 × *g*, 60 µl of the total glass beads extract (A_260_ between 80 and 100 mAU) was loaded onto a 90 µl low salt sucrose cushion (25% sucrose, 20 mM Hepes/KOH pH 7.4, 120 mM KOAc, 2 mM Mg(OAc)_2_, 2 mM DTT, 1 mM PMSF, protease inhibitor mix) or a 90 µl high-salt sucrose cushion (25% sucrose, 20 mM Hepes/KOH pH 7.4, 800 mM KOAc, 2 mM Mg(OAc)_2_, 2 mM DTT, 1 mM PMSF, protease inhibitor mix). After centrifugation at 400,000 × g at 4 °C for 25 min, the cytosolic supernatant was collected and the ribosomal pellet was resuspended in 300 µl ribosome-binding buffer. Aliquots of the total cell extract, cytosolic supernatant, and resuspended ribosomes were precipitated by addition of TCA to a final concentration of 5%. TCA pellets were dissolved in SDS sample buffer (20 mM Tris/HCl pH 6.8, 1% SDS, 5 mM EDTA, 0.08% bromphenolblue, 10% glycerol,) and were analyzed on 10% Tris-Tricine gels, followed by immunoblotting. Sse1 or Pgk1 served as cytosolic marker; Rpl35, Rpl31, or Rpl24 served as ribosomal markers.

### Ribosome profile analysis

Ribosome profiles were performed with total glass beads extract prepared as described for the ribosome-binding assay. Total glass beads extract corresponding to 10 *A*_260_ units was loaded onto an 11 ml 15–55% linear sucrose gradient prepared in ribosome-binding buffer (Gradient Master 108, Biocomp). Samples were then centrifuged for 2.5 h at 200,000 × *g* (TH641, Sorvall), and fractionated into 560 µl aliquots with a gradient fractionator monitoring *A*_260_ (Piston Gradient Fractionator, Biocomp). A total corresponding to 5% of the material loaded onto the gradient, aliquots of fractions 1–8 (560 µl each), combined fractions 9–19 (6.2 ml), and the resuspended pellet, which had formed at the bottom of the gradient tube were precipitated by the addition of TCA, and were analyzed on Tris-Tricine gels followed by immunoblotting. Please note, that combined fractions 9–19 correspond to 11 aliquots when compared with the individual fractions 1–8.

### Protein–RNA CRAC

Yeast strains expressing HTP-tagged Get3, Get4, or Sgt2 from their genomic loci or plasmid-derived HTP-tagged Get5 were analyzed by PAR-CRAC^33,69^. Cells were grown exponentially in low uracil media (10 mg/l uracil) supplemented with 100 μM 4-thiouracil before growth for an additional 4 h in the presence of 1 mM 4-thiouracil. 4-thiouridine (4sU)-containing RNAs were crosslinked to associated proteins using 600 mJ cm^−2^ irradiation at 365 nm. Protein–RNA complexes were isolated under native conditions on IgG sepharose and then under denaturing conditions on Ni-NTA. A partial RNase digest was performed using RNace-IT, and co-purified RNAs were 5′ labeled with [^32^P] and were ligated to 3′ and 5′-sequencing adaptors. The 5´adaptor contained an NNNNNAGC unique molecular identifier sequence (UMI) to allow consolidation of multiple sequencing reads derived from the same RNA template. Complexes were separated by NuPAGE and transferred to a nitrocellulose membrane. RNAs were released from the membrane by Proteinase K digestion, isolated and reverse transcribed. The cDNA libraries were amplified by PCR (wild type: 35 cycles, Get4-HTP: 24 cycles) and subjected to Illumina deep sequencing. Sequencing reads were trimmed and quality controlled using Flexbar^70^, reads shorter than 18 nucleotides were discarded. Identical sequencing reads containing the same UMI were collapsed to a single read. The remaining sequences were mapped to the *S. cerevisiae* genome using Bowtie 2^71^. Alignments containing no, or a single, mismatch were allowed and reads were then filtered to retain only those reads containing a single T-C mutation induced by the presence of 4sU. For generating heat maps showing the number of reads mapping to different nucleotides on the secondary structure of the 25S rRNA^72^ and the tertiary structure of the ribosome (PDB 4V88), programming scripts in the programming language Python (version 3.5.2) were used.

### In vitro transcription and translation

DNA templates for transcription reactions were generated by PCR using pSP65-FLAG-Sec22+60 or pSP65-Sec22+60 as templates^42^. Reverse primers were designed such that PCR products encoded stop codon-less Sec22, Sec22+10, Sec22+20, Sec22+30, Sec22+40, or Sec22+60, or the N-terminally FLAG-tagged versions, respectively. In order to generate full-length released Sec22 or FLAG-Sec22 reverse primers containing the *UAG* stop codon were designed. Transcripts were generated using SP6 polymerase (ThermoFischer Scientific)^73^. Yeast translation extract was prepared from yeast strains as indicated in Results and Figure Legends. Cells harvested from 10 l cultures were resuspended in 200 ml sorbitol buffer (1.4 M sorbitol, 50 mM K-phosphate buffer pH 7.4, 10 mM DTT) containing 2 mg zymolyase 20 T (Nacalai Tesque Inc.) g^−1^ cells. After incubation at 30 °C for 30 min, spheroblasts were collected at 4 °C and were resuspended in 300 ml sorbitol buffer (YPD medium containing 1 M sorbitol). After incubation at 22 °C for 90 min, spheroblasts were re-collected, and resuspended in 400 ml sorbitol buffer. The spheroblast suspension was transferred into two 500 ml JA-10 (Beckman Coulter) centrifuge tubes and underlaid with 200 ml of cold sorbitol buffer. After centrifugation at 4000 × *g* for 7 min (JA-10), spheroblasts were washed twice with cold sorbitol buffer and resuspended in 5-10 ml lysis buffer (20 mM Hepes/KOH pH 7.4, 100 mM KOAc, 2 mM MgOAc_2_, 2 mM DTT, 0.5 mM PMSF, 1× protease inhibitor mix). The spheroblast suspension was transferred into a 40 ml dounce homogenizer (Kontes Glass Co.) and spheroblasts were disrupted on ice by douncing with a type B pestle. The resulting extract was centrifuged at 27,000 × *g* in a SS34 rotor (Piramoon Technologies Inc.) for 18 min at 4 °C. The supernatant was collected and centrifuged at 100,000 × *g* in a 70.1 Ti rotor (Beckman Coulter) for 35 min at 4 °C. The supernatant was loaded onto a Superdex-G25 gel filtration column equilibrated with gel filtration buffer (20 mM Hepes/KOH pH 7.4, 100 mM KOAc, 2 mM MgOAc_2_, 2 mM DTT, 0.5 mM PMSF, 20% glycerol) at a flow rate of 1.5 ml min^−1^. Peak fractions with the highest A_260_ were pooled, were supplemented with 1 mM CaCl_2_, were treated with 300 U ml^−1^ micrococcal nuclease (Roche) for 15 min at 20 °C followed by addition of 2 mM EGTA, and aliquots of the resulting yeast translation extract, were stored at −80 °C^74,75^. RNCs were generated via translation reactions primed with the stop codon-less transcripts at 20 °C for 80 min^42,74^. RNCs for chemical crosslinking were generated in the presence of [^35^S]-methionine (Hartmann-Analytic). RNCs were isolated via centrifugation at 400,000 × *g* for 20 min as described^42^. Released Sec22 or FLAG-Sec22 chains for pull-down assays were labeled with [^35^S]-methionine and were isolated away from ribosomes via centrifugation at 400,000 × *g* for 20 min. As indicated purified His_6_Get4/Get5 was added to in vitro translation reactions to a final concentration of 2 µM.

### Chemical crosslinking of His_6_Get5 and Ni-NTA purification under denaturing conditions

Total cell extracts were prepared according to the CryoMill protocol (Retsch^®^). CryoMill powder was dissolved in lysis buffer (20 mM Hepes/KOH pH 7.4, 2 mM Mg(OAc)_2_, 120 mM KOAc, 50 µg ml^−1^ cycloheximide, 2 mM DTT, 1 mM PMSF, protease inhibitor mix) and ribosomes were isolated by a centrifugation step at 400,000 × *g* at 4 °C for 25 min. BS^3^ (bis-(sulfosuccinimidyl)-suberate, spacer length 1.14 nm, Thermo Scientific) was added to resuspended ribosomes to a final concentration of 150 µM, and samples were incubated on ice for 15 min. Crosslinking was then quenched by the addition of Tris base (tris(hydroxymethyl)aminomethane) to a final concentration of 50 mM. Crosslinked ribosomes were re-isolated by ultracentrifugation as described above and ribosomal pellets were resuspended in denaturing binding buffer (8 M urea, 0.1 M sodium phosphate buffer, 0.01 M Tris/HCl, pH 8.0). The material was applied to Ni-NTA super flow beads according to the manufacturer’s manual under denaturing conditions (QIAGEN) using denaturing wash buffer (8 M urea, 0.1 M sodium phosphate, 0.01 M Tris/HCl pH 6.3) and, for the elution of His_6_Get5 and crosslinked proteins denaturing low-pH elution buffer (8 M urea, 0.1 M sodium phosphate, 0.01 M Tris/HCl pH 4.5). TCA was added to the eluates to a final concentration of 5% and protein pellets were dissolved in SDS sample buffer and analyzed on Tris-Tricine gels followed by immunoblotting or analysis by mass spectrometry (Fig. 2a).

### Mass spectrometry

Crosslinked samples were diluted in NuPAGE SDS sample buffer and were run on 4–12% NuPAGE gels (Supplementary Fig. 2c). Each sample was cut into 11 slices and was digested with trypsin. Following digestion, two technical replicates of LC-MS-MS were performed. All peptides were screened against a UniProt yeast database and further analyzed via the Scaffold 4.9.0. software (Supplementary Dataset 1 and Source data file). Selection of ribosomal crosslinking partners of His_6_Get5 (Table 1) is described in Results. Yeast possesses two paralogues (designated a and b) of many ribosomal protein genes. The corresponding proteins often differ in only a few residues. If the mass spec results did not allow to distinguish between a and b forms, the data were included into the same cluster.

### Chemical crosslinking of RNCs and denaturing immunoprecipitation of crosslink products

RNCs prepared by in vitro translation (see above) were separated from cytosolic components and released nascent chains by centrifugation through a sucrose cushion at 400,000 × *g* for 20 min. For that purpose, 60 µl translation reaction were loaded onto a 90 µl cushion (25% sucrose in resuspension buffer: 20 mM Hepes/KOH pH 7.4, 2 mM Mg(OAc)_2_, 120 mM KOAc, 1 mM PMSF, protease inhibitor mix). After resuspension of RNCs in resuspension buffer crosslinking was performed by the addition of BS^3^ to a final concentration of 400 µM for 20 min on ice. Crosslinking reactions were then quenched by the addition of Tris base to a final concentration of 50 mM. Crosslinked protein samples were precipitated by the addition of TCA (final concentration 5%). Pellets were collected by centrifugation and were dissolved in dissociation buffer (200 mM Tris/HCl pH 7.5, 4% SDS; 10 mM EDTA, 100 µg ml^−1^ BSA, protease inhibitor mix, 1 mM PMSF). The resulting denatured protein samples were used to identify crosslink products between SRP, Get4/5, or Sgt2 and nascent chains via affinity purification. For this purpose, protein A sepharose beads (GE Healthcare) were pre-coated with antibodies (Eurogentec) directed against Srp54, Get4, Get5, or Sgt2 and subsequently the denatured crosslinked material was allowed to bind to the beads^42^. Crosslink products bound to protein A sepharose beads were released by addition of SDS sample buffer at 95 °C for 10 min. Samples were analyzed on 10% Tris-Tricine gels followed by autoradiography.

### FLAG-tag pull-down reactions

FLAG-tag pull-down experiments were performed as described^20^. RNCs were separated from cytosolic components by centrifugation through a sucrose cushion as described above, were resuspended in resuspension buffer and were then added to 20 μl of pre-washed Anti-FLAG^®^ M2 affinity beads (FLAG-beads, Sigma) resuspended in 600 μl resuspension buffer. Non-tagged and N-terminally FLAG-tagged RNCs were analyzed in parallel in each experiment to determine background binding (Supplementary Fig. 4b and Supplementary Fig. 5f). Material bound to FLAG-beads was separated on 10% Tris-Tricine gels, transferred to nitrocellulose membranes, and was analyzed via immunoblotting. In case FLAG-tag pull-down experiments were performed with released Sec22 chains, the samples were diluted 1:7 with resuspension buffer and were then subjected to FLAG-tag pull-down reactions. After the binding reaction, FLAG-beads were washed with resuspension buffer and bound material was eluted by incubation with SDS sample buffer at 30 °C for 20 min. Subsequently, SDS sample buffer was removed from the beads, cleared by centrifugation at 5000 × *g* for 30 sec, transferred into a fresh 1.5 ml tube, and boiled at 95 °C for 5 min.

### Quantification of ribosome-bound Sgt2 in FLAG-tag pull-down reactions

The relative occupancy of RNCs with Sgt2 was determined by quantitative analysis of experimental data as shown in Fig. 4f. For each replicate, the amount of Sgt2 and RNCs (Rps9) was determined by densitometry. As a first step, Sgt2 values were normalized for the amount of RNCs (ratio: Sgt2/Rps9). Normalized Sgt2 values obtained for the Δ*srp54* samples were set to 1. The amount of Sgt2 bound to wild-type RNCs-Sec22+60 without addition of purified His_6_Get4/Get5 was below the detection limit of ImageJ.

### Quantification of the capture of released Sec22 by Sgt2

The relative efficiency with which Sgt2 was bound to released Sec22 was determined by quantitative analysis of data as shown in Fig. 5e. For each replicate, the relative amount of Sgt2 was determined by densitometry of immunoblots. In parallel, the amount of [^35^S]-labeled Sec22 was determined by autoradiography. Sgt2 values were normalized for the amount of [^35^S]-labeled Sec22 (ratio: Sgt2/Sec22) and normalized Sgt2 values in the Δ*get3* samples were set to 1.

### Statistics and reproducibility

The following experiments were repeated independently with similar results and represent biological replicates. Figure 1a: *n* = 3. Figure 1c: *n* = 3 for each concentration, except for the measurements at 0.1 µM 80S ribosome concentration, which was *n* = 2. Figure 1d: *n* = 6. Figure 2b–d: *n* = 2. Figure 3b: *n* = 2. Figure 4b: *n* = 2. Figure 4c: *n* = 3. Figure. 4d, e: *n* = 2. Figure. 4f, g: wild type and Δ*srp54* *n* = 5, wild type + His_6_Get4/Get5 and Δ*srp54* + His_6_Get4/Get5 *n* = 2. Figure 5a: *n* = 1. Figure. 5b–d: *n* = 2. Figure 5e: *n* = 3. Figure 6a: *n* = 3. Figure. 6c–f: *n* = 2. Supplementary Fig. 1: *n* = 2. Supplementary Fig. 2a, b: *n* = 2. Supplementary Fig. 2c: *n* = 2 (technical replicates of LC-MS). Supplementary Fig. 3a–e: *n* = 1. Supplementary Fig. 3f: *n* = 2. Supplementary Fig. 4c, 4d, and 4f: *n* = 1. Supplementary Fig. 4e, j, and 4k: *n* = 2. Supplementary Fig. 5b–d, 5g, and 5h: *n* = 2.

### Miscellaneous

Immunoblots were developed using an ECL camera (Fusion Pulse 6, Vilber). Autoradiographs were analyzed and quantified with a Typhoon 9410 (GE Healthcare Life Sciences). Quantitative analysis of immunoblots was performed using ImageJ 1.52a (National Institutes of Health, USA). Statistical analysis was performed with GraphPad Prism 6.07. Models of ribosomal particles were generated with PyMOL 2.2.0. Polyclonal antibodies directed against Srp54^42^ (1:4000), Sgt2^24^ (1:5000), Get4^24^ (1:5000), Get5^24^ (1:5000), Get3^24^ (1:5000), Rpl4^24^ (1:10,000), Rpl24^42^ (1:10,000), Rpl35^60^ (1:10,000), Rpl31^60^ (1:5000), Rpl26^60^ (1:2000), Rps9^42^ (1:5000), Pgk1^75^ (1:2000), Kar2^76^ (1:2000), Sse1^42^ (1:20,000) (Eurogentec, Rospert lab antibody collection), and PAP (Sigma ID P1291, 1:5000) were raised in rabbit. α-FLAG (Sigma F3165, 1:2000) and α-His (Qiagen 34660, 1:2000) are monoclonal antibodies. Horseradish peroxidase-conjugated protein A (ThermoFischer Scientific 101023, 1:5000) was employed as a secondary reagent to detect primary antibodies. Dilutions employed for immunoblotting experiments are given in brackets. References, which include validation data of the different antibodies are indicated.

### Reporting summary

Further information on research design is available in the Nature Research Reporting Summary linked to this article.

## Supplementary information

Supplementary Information

Peer Review File

Supplementary Dataset 1

Reporting Summary

Description of Additional Supplementary Files

## Data Availability

The CRAC data sets of Get4-HTP and the wild type yeast control are deposited in the Gene Expression Omnibus database under the accession code GSE151664. Other data that support the findings of this study and biological materials are available from the corresponding authors on reasonable request. Source data are provided with this paper.

## Source data

Source Data
