# Supplementary material for: Ribosome-bound Get4/5 facilitates the capture of tail-anchored proteins by Sgt2 in yeast
Source: Nat Commun. 2021 Feb 4;12:782. doi: 10.1038/s41467-021-20981-3 (PMC7862611; doi:10.1038/s41467-021-20981-3)
Supplement: Supplementary file 1 — Supplementary Information [file 41467_2021_20981_MOESM1_ESM.pdf]

Ribosome-bound Get4/5 facilitates the capture of tail-anchored proteins by Sgt2 in yeast

Ying Zhang[1,2,6], Evelina De Laurentiis[3,4,6], Katherine E. Bohnsack[3], Mascha Wahlig[1,2], Namit Ranjan[5], Simon Gruseck[1,2], Philipp Hackert[3], Tina Wölfle[1,2], Marina V. Rodnina[4,5], Blanche Schwappach[3,4]✉, and Sabine Rospert[1,2]✉

[1]Institute of Biochemistry and Molecular Biology, ZBMZ, Faculty of Medicine, University of Freiburg, D-79104 Freiburg, Germany

[2]BIOSS Centre for Biological Signaling Studies, University of Freiburg, D-79104 Freiburg, Germany

[3]Department of Molecular Biology, University Medical Center Göttingen, Humboldtallee 23, D-37073 Göttingen, Germany

[4]Cluster of Excellence "Multiscale Bioimaging: from Molecular Machines to Networks of Excitable Cells" (MBExC)

[5]Max-Planck Institute for Biophysical Chemistry, Am Faßberg 11, D-37077 Göttingen, Germany

[6]These authors contribuated equally: Ying Zhang, Evelina De Laurentiis

✉email: blanche.schwappach@med.uni-goettingen.de; sabine.rospert@biochemie.uni-freiburg.de

Supplementary Information

Supplementary Notes 1 - 3

Supplementary Methods

Supplementary Figures and Figure Legends 1 - 5

Supplementary Tables 1 - 4

Supplementary References

Supplementary Notes

Supplementary Note 1. Estimation of Get4/5 affinity for ribosomes in cell extract. To estimate an apparent K_d value in yeast cell extract, we made the following assumptions: i) the volume of a haploid yeast cell is ~ 70 μm^3 [1]; ii) a yeast cell contains 5000 $Get4_2Get5_2$ complexes [2] and 300000 ribosomes [3]; iii) an OD_{600} of 1 in our experimental setup corresponds to 4.48 x 10^7 cells ml^{-1} [3]. Based on these assumptions, the concentration of $Get4_2Get5_2$ in a yeast cell is ~ 120 nM and of ribosomes 7 µM. The yeast cell content is diluted 9.6-fold in ribosome binding experiments. In these experiments about 40% of Get4/5 was associated with ribosomes (Fig. 1a and 1b). We estimate an apparent K_d of 1.1 µM for the binding of $Get4_2Get5_2$ to ribosomes in cell extract, which is 10-fold above the K_d value of 110 ± 40 nM determined with purified components (Fig. 1c).

This 10-fold increase is likely due to a number of differences between the two experimental approaches. One important limitation of ribosome binding experiments in cell extract is that they are not performed at equilibrium. Moreover, one has to consider that the free concentration of $Get4_2Get5_2$ is likely lower than assumed above, because Get4/5 binds to Get3 with a K_d of 3.2 nM in the presence of ATP [4]. The UBL domain of Get5 binds to the N-terminus of Sgt2 with a K_d of 50 nM [5]; likely the affinity between the full-length proteins is even higher. Thus, a fraction of Get4/5 is associated with the soluble GET components ([6,7] and Supplementary Fig. 5g and 5h). As binding of Get4/5 to cytosolic Sgt2 and Get3 is likely competitive with binding to ribosomes, this will increase the apparent K_d for Get4/5 binding to ribosomes in yeast cell extract (see also Supplementary Note 2).

Supplementary Note 2. Get4/5 binds to RNCs even when Sgt2 and Get3 are absent from the reaction. In the course of this study, we have performed experiments with ribosome nascent chain complexes (RNCs) (see Figs. 4 and 5). In some of these experiments, RNCs were affinity purified via a FLAG-tag at the N-terminus of the nascent chain and copurification of associated factors was analyzed (Supplementary Fig. 4b). RNC preparations used for this type of experiment need to be free of ribosome-released FLAG-tagged chains. To remove trace amounts of released nascent chains, RNCs were collected by ultracentrifugation at 400000 x g and were then carefully resuspended (see Methods). Of note, RNC preparations also contain vacant 80S ribosomes, which cosediment with RNCs at 400000 x g. Get4/5 and Sgt2 was bound to RNCs generated by this method (Figs. 4 and 5, Supplementary Figs. 4 and 5).

To unequivocally demonstrate that Get4/5 binds to RNCs, rather than being bound to a minor contamination of released chains, we performed additional control experiments. Although Get4/5 itself does not directly interact with cytosolic TA proteins ([8,9] and Supplementary Fig. 5h), Get4/5 can interact with Sgt2 via the Get5 subunit and with Get3 via the Get4 subunit. Because Sgt2 and Get3 directly bind to TA proteins after ribosome-release [8,9], it is feasible that Get4/5 binds to released TA proteins via Sgt2 or Get3. To exclude this possibility, we performed FLAG-tag pull down experiments side by side in a wild type and a *Δget3Δsgt2* translation extract (Supplementary Fig. 4e). Get4/5 copurified with FLAG-tagged RNCs even in the absence of Sgt2 and Get3. The finding confirms that Get4/5 binds to RNCs, rather than to Sgt2/TA protein or Get3/TA protein complexes. Interestingly, the experiment revealed that more Get4/5 co-purified with RNCs in the absence of Sgt2 and Get3 (Supplementary Fig. 4e). This was not due to Get4/5 aggregation, as the - FLAG control was free of Get4/5

(Supplementary Fig. 4e). The most likely explanation for this observation is that ribosomes on the one side, and Sgt2 and/or Get3 on the other, compete for binding to Get4/5 (Supplementary Note 1). According to this model, more Get4/5 is available to bind to RNCs/ribosomes when the competing factors Sgt2/Get3 are absent from the reaction.

Supplementary Note 3. Estimation of relative affinities of Get4/5 and SRP for RNCs. RNCs analyzed via FLAG-tag pull down experiments (Supplementary Fig. 4b) were generated in a yeast translation extract (see Methods). In order to estimate the fraction of ribosomes (Rps9), Get4/5 (Get4), and SRP (Srp54) isolated via FLAG-tagged nascent chains, we used the intensities of immunostained Rps9, Get4, and Srp54 bands in the total translation reactions as reference values (Supplementary Fig. 4c, input 1% - 4%). The analysis revealed that 1.7% - 3.5% of ribosomes present in the translation reaction were isolated as RNCs via the nascent chain FLAG-tag (Supplementary Fig. 4c, Rps9 [% of input]). This value corresponds well with the observation that approximately 2% of ribosomes in a yeast translation extract translate exogenously added transcripts [3]. According to this analysis 3.2 - 6% of Get4/5 and 2.2 - 3.3% of SRP was bound to RNCs with a nascent TA sequence inside of the ribosomal tunnel (Supplementary Fig. 4c, lanes Sec22, Sec22+10, and Sec22+20, Get4 and Srp54 [% of input]).

According to published work the ratio between $Get4_2Get5_2$:SRP:ribosomes inside a yeast cell is about 1:1.5:60 ([2,3] and see Discussion). Thus, the number of $Get4_2Get5_2$ and SRP complexes in a cell is rather similar. The ratio of soluble cytosolic proteins such as Get4/5 and SRP remains largely unchanged in a translation extract, which is basically a cytosol preparation (Supplementary Fig. 4d and see Methods). If this assumption is correct, the number of Get4/5 and SRP complexes in the input material is similar, and also the fraction of these complexes bound to RNCs-Sec22, -Sec22+10, and -Sec22+20 is similar (Supplementary Fig. 4c, Get4 and Srp54 [% of input]). Accordingly, the affinities of Get4/5 and SRP for RNCs with a nascent TA sequence inside of the tunnel likely lie within the same order of magnitude. The estimate corresponds well with the observation that the affinities of Get4/5 and SRP for empty ribosomes are also similar (see Discussion). Notably, upon exposure of the TA sequence outside of the ribosomal tunnel, the affinity of SRP dramatically increases (Fig. 4c). Consistent with previous data [10,11] a larger fraction of Get4/5 was bound to RNCs-Pgk1-100 when compared to SRP (Supplementary Fig. 4c, lanes RNCs-Pgk1-100). This observation suggests that Get4/5 displays a higher affinity than SRP for RNCs carrying soluble nascent chains.

Supplementary Figures

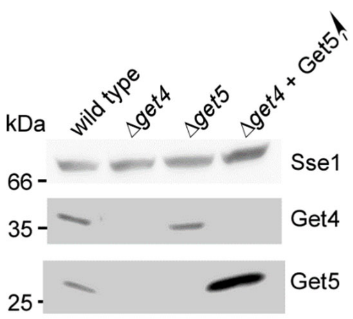

Supplementary Figure 1. Get5 is strongly destabilized in a Δ*get4* strain. Immunoblot of total cell extract derived from wild type, Δ*get4,* Δ*get5,* and Δ*get4* + Get5↑ strains decorated with α-Get4, α-Get5, and α-Sse1 (loading control).

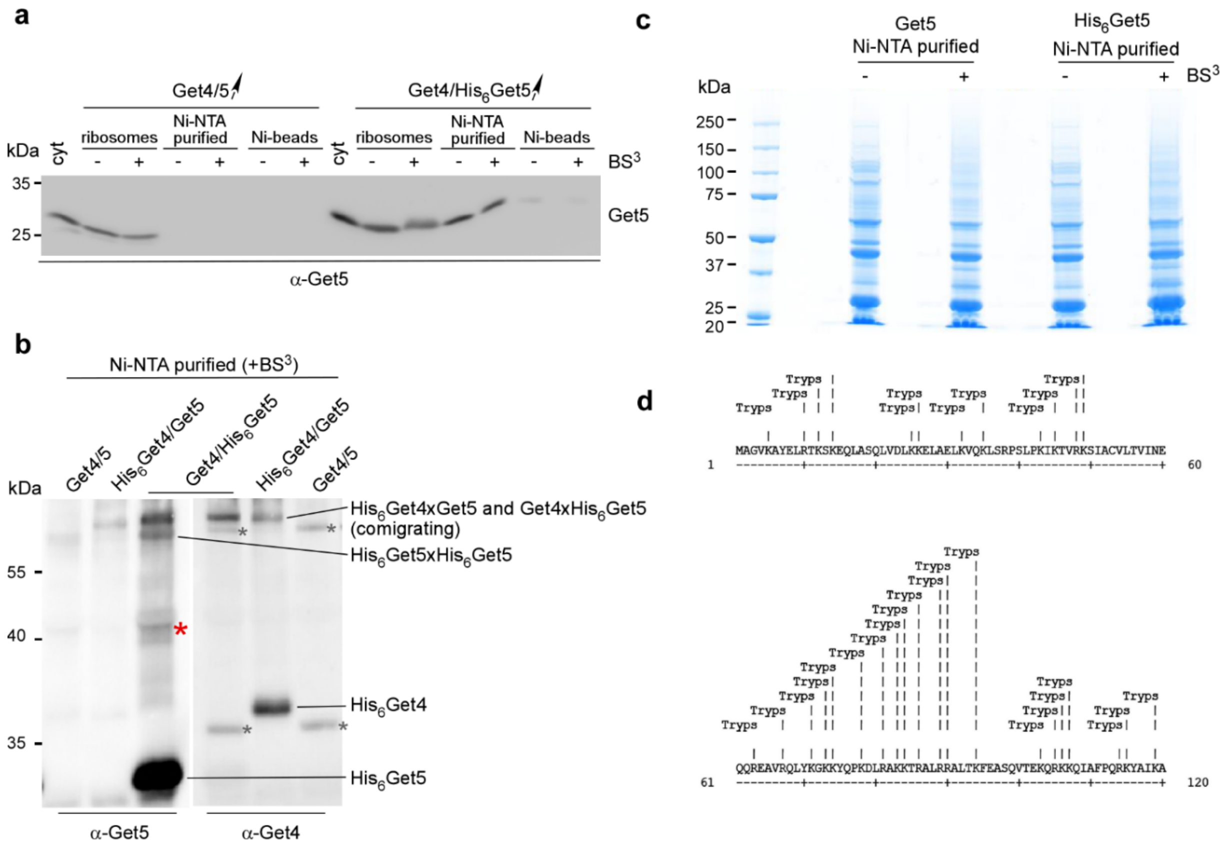

Supplementary Figure 2. Analysis of protein crosslinks to ribosome-bound Get5. (a) Crosslinking and Ni-NTA purification of His6Get5 under denaturing conditions. Total extract of strains overexpressing either Get4/5 or Get4/His6Get5 were separated into cytosolic fractions (cyt) and ribosomal pellets (ribosomes) under low salt conditions as described in Fig. 1a. Subsequently, ribosomal pellets were resuspended and half of each sample was crosslinked with BS^3, while the other half was incubated without BS^3 as a control. Samples were denatured and His6Get5 and proteins crosslinked to it were purified via Ni^{2+}-NTA. His6Get5 was eluted from the Ni-beads by applying low-pH buffer (Ni-NTA purified). Residual His6Get5 represents material which remained bound to the Ni-beads and was released after boiling the beads in sample buffer (Ni-beads). For details see Methods and Fig. 2a. Shown is an immunoblot decorated with α-Get5. **(b)** Identification of major Get4 and Get5 crosslink products. Total extract of strains overexpressing Get4/His6Get5 or His6Get4/Get5 in a Δ*get4*Δ*get5* background and a strain overexpressing Get4/5 were treated as described in panel a (see also Fig. 2a). Isolated His6-tagged proteins were analyzed via immunoblotting with α-Get4 or α-Get5. Side-by-side analysis

revealed His_6Get5xHis_6Get5, His_6Get4xGet5, and Get4xHis_6Get5 as major crosslink products. The crosslink product at ~ 42 kDa in the Get4/His_6Get5 sample labeled with the red asterisk is likely a mixture of His_6Get5xRpl26 and His_6Get5xRpl35 (see Fig. 2d). A number of less abundant crosslink products were detected by α-Get5 in the Get4/His_6-Get5 sample. Of note, α-Get4 recognized His_6Get4xGet5 as well as His_6Get5xGet4, but no other bands. Black asterisks label unspecific background bands recognized by α-Get4. **(c)** Ni-NTA purified crosslinking products for mass spectrometry. Aliquots of Ni-NTA purified samples as shown in panel a were separated via SDS-PAGE and were stained with Colloidal Coomassie (G250) for further analysis via mass spectrometry as detailed in Methods. **(d)** Cleavage prediction of Rpl35 upon trypsin digestion. Crosslinked protein samples for mass spectrometry were digested with trypsin. The predicted trypsin cleavage pattern of Rpl35a [12] reveals numerous cleavage sites.

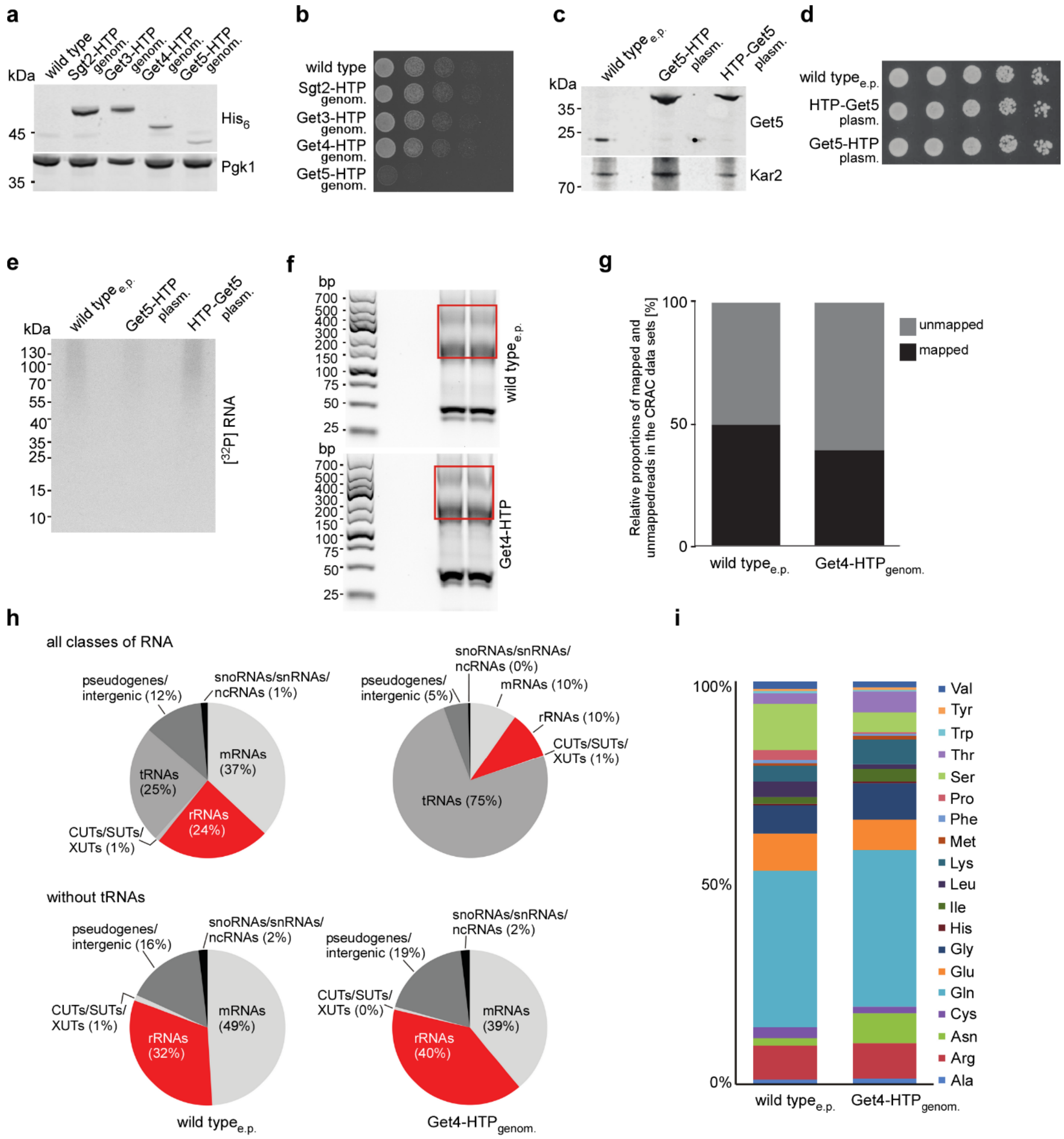

Supplementary Figure 3. Ribosomal contacts of Get4/5.

j

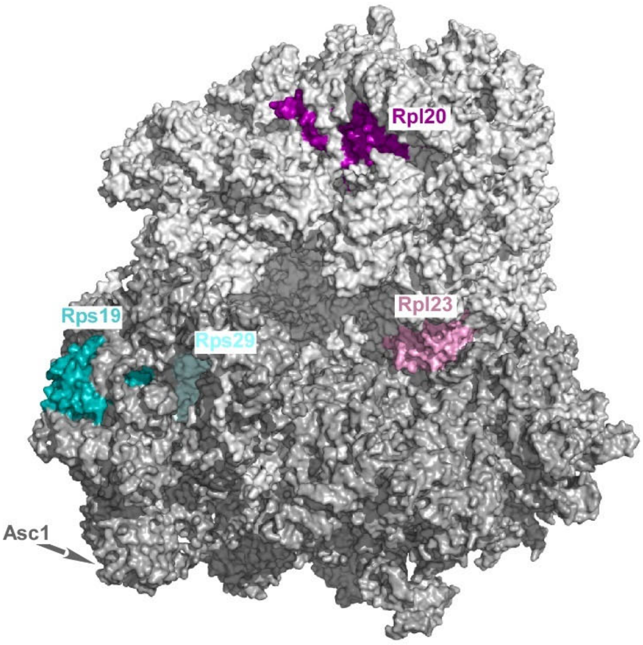

k

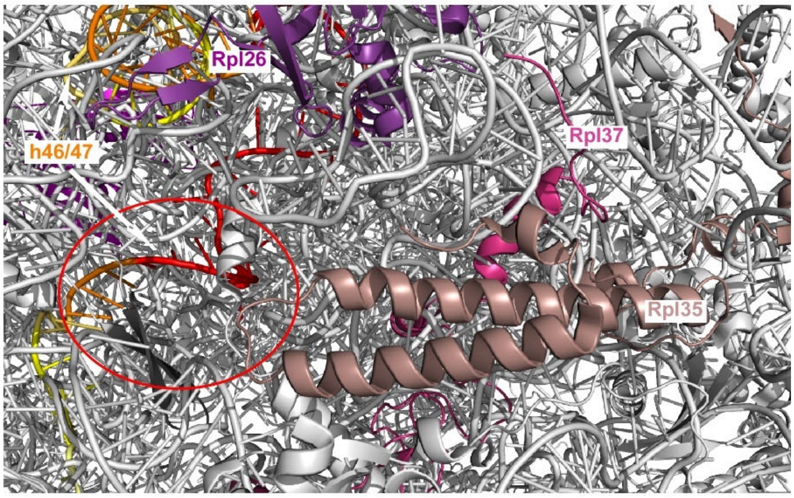

l

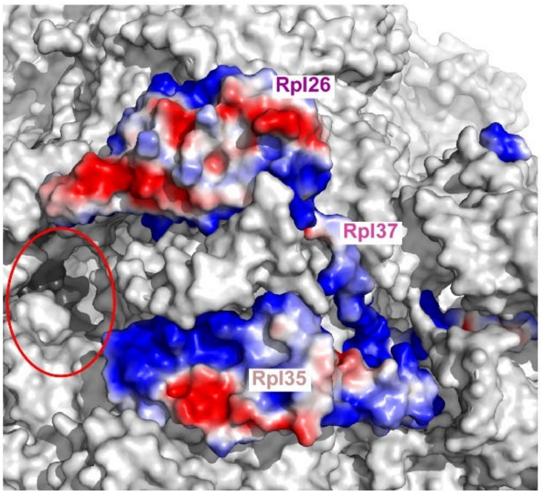

Supplementary Figure 3. **Ribosomal contacts of Get4/5** (continued)**. (a)** Expression of genomically HTP-tagged Sgt2, Get3, Get4, or Get5. Total protein extracts were prepared from strains expressing C-terminally HTP-tagged GET components as indicated. Samples were analyzed by immunoblotting using

α-His for the detection of HTP-tagged proteins and α-Pgk1 as a loading control. Blots were scanned using an Odyssey CLx infrared scanner (LI-724 COR Biosciences) with LiCOR 926-32213, 926-68023, or LiCOR 926-32212 as secondary antibodies (dilution 1:10000). **(b)** Phenotypic analysis of yeast strains expressing Get4-HTP, Get5-HTP, Sgt2-HTP, and Get3-HTP. Strains as shown in panel a were grown to log phase and subsequently serial 5-fold dilutions were spotted onto a YPD plate, which was incubated at 30° C for 2 days. The growth defect observed for the Get5-HTP$_{genom.}$ strain is likely due to an unwanted genomic event that occurred during the tagging procedure. The Get5-HTP$_{genom}$. strain was thus omitted from further analysis. Please note, that a Δ*get5* strain expressing Get5-HTP from a low copy plasmid displayed wild type growth (see panel d). **(c)** Expression of plasmid-derived Get5-HTP and HTP-Get5. Total protein extracts from Δ*get5* strains expressing Get5-HTPor HTP-Get5, and as a control wild type$_{e.p.}$ that harbors the empty pRS415 plasmid, were analyzed via immunoblotting using α-Get5 and α-Kar2 as a loading control. Blots were analyzed as described in panel a. **(d)** Growth of yeast strains expressing plasmid derived Get5-HTP or HTP-Get5. Phenotypic analysis of yeast strains as shown in panel c. Strains were grown to log phase in SD medium and subsequently 5-fold serial dilutions were spotted onto SD plates, which were incubated at 30° C for 2 days. **(e)** CRAC analysis of Get5-HTP and HTP-Get5. Shown is an autoradiography (16 h exposure) of the material after ProtA purification, RNA trimming, and [^{32}P]-labeling. The corresponding immunoblot is shown in panel c. **(f)** PCR amplification of wild type and Get4-HTP cDNA libraries. After reverse transcription, cDNA libraries were PCR amplified using 35 cycles (wild type) or 24 cycles (Get4-HTP). PCR products were separated by agarose gel electrophoresis, were visualized by SyberGold staining and the area of the gel indicated by the red box was excised. PCR products were extracted from the gel, purified and analyzed by Illumina next-generation sequencing. **(g)** Mapped *versus* unmapped reads in the CRAC data sets. CRAC data presented in the manuscript were normalized (per million mapped reads) before further analysis. **(h)** Relative distribution of reads derived from different classes of RNA. The relative proportions of reads derived from different classes of RNAs is shown for the wild type and Get4-HTP CRAC samples. Relative proportions of reads derived from all classes of RNA (upper panel) and without those derived from tRNAs (lower panel). Ribosomal RNAs (rRNAs), messenger RNAs (mRNAs), cryptic unstable transcript/stable uncharacterized transcripts/Xrn1-sensitive unstable transcripts (CUTs/SUTs/XUTs), small nucleolar RNAs (snoRNAs), small nuclear RNAs (snRNAs) non-coding RNAs (ncRNAs), transfer RNAs (tRNAs), pseudogenes/intergenic regions. **(i)** Relative distribution of reads derived from different tRNA isoacceptors. The relative proportions of reads derived from different tRNA isoacceptors is shown for the wild type and Get4-HTP CRAC samples. **(j)** Ribosomal proteins and rRNA contacts of Get4/5 distant from the tunnel exit. Surface representation of the small and large subunit of the yeast ribosome (PDB 4V88 [13]) in light gray (60S) and gray (40S). The position of the small ribosomal subunit protein Asc1 is indicated for orientation. Rpl20 (light magenta), Rpl23 (light pink), Rps19 (teal) and Rps29 (pale cyan). See also Table 1. **(k)** Cartoon representation of the ribosomal tunnel exit region with a focus on h46/h47. Rpl26 (deep purple), Rpl35 (dirty violet), Rpl37 (hot pink). h46 and h47 of the 25S rRNA, which are contacted by Get4, are highlighted in the same shades of yellow to red as in Fig. 3d. The tunnel exposed β-sheet of Rpl17 is indicated in black. For a surface representation see Fig. 3f. **(l)** Surface exposed positively charged residues of Rpl26, Rpl35, and Rpl37. Electrostatic surface potential of Rpl26, Rpl35, and Rpl37 assembled into 60S particles. -2 kT/e (deep red) to +2 kT/e (deep blue). The tunnel-exposed β-sheet of Rpl17 is indicated in black. The tunnel exit region is indicated by the red circle.

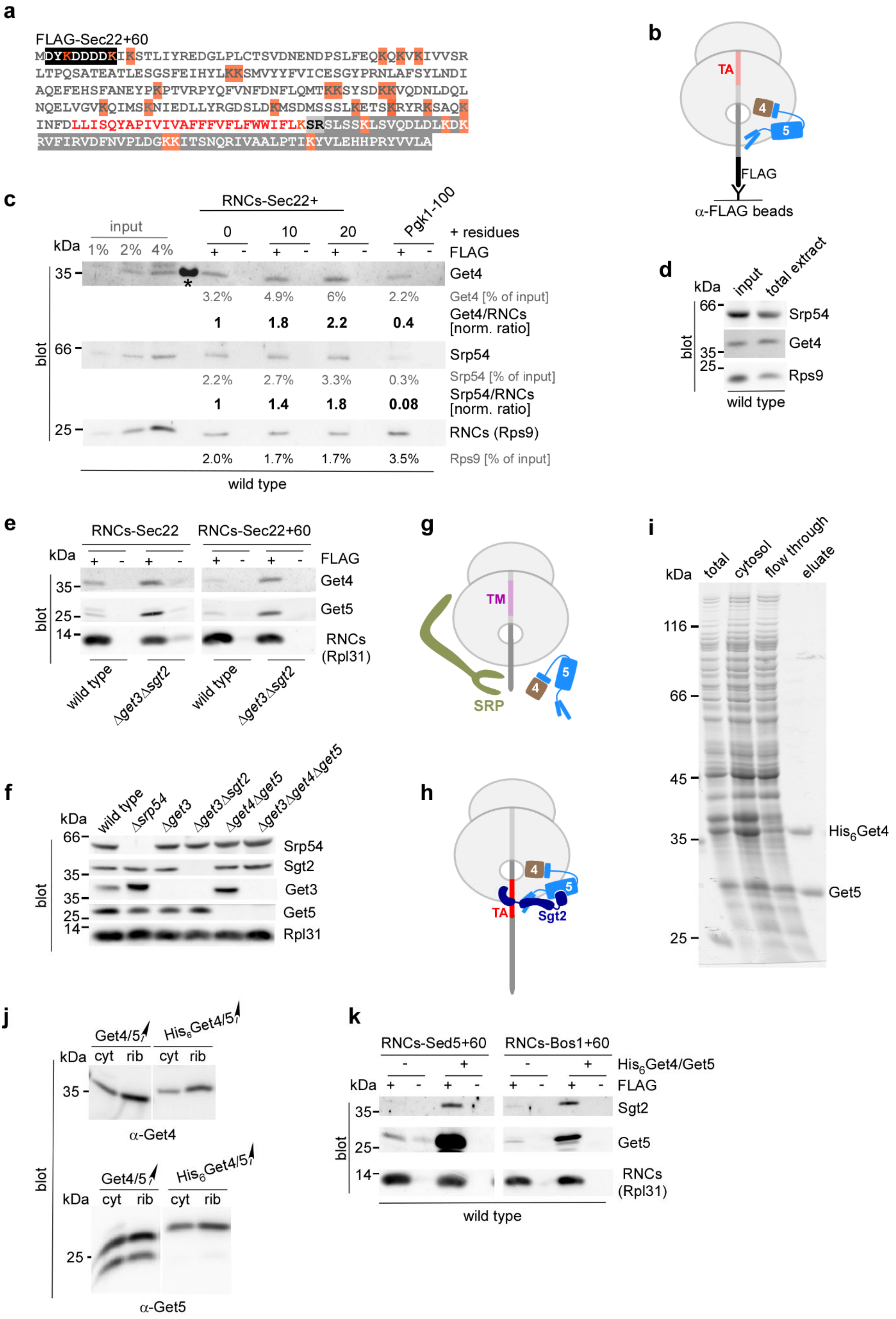
a
FLAG-Sec22+60
MDYKDDDDKIKSTLIYREDGLPLCTSVDNENDPSLFEQKQKVKIVVSR
LTPQSATEATLESGSFEIHYLKKSMVYYFVICESGYPRNLAFSYLNDI
AQEFEHSFANEYPKPTVRPYQFVNFDNFLQMTKKSYSDKKVQDNLDQL
NQELVGVKQIMSKNIEDLLYRGDSLDKMSDMSSSLKETSKRYRKSAQK
INFDLLISQYAPIVIVAFFFVFLFWWIFLKSRSLSSKLSVQDLDLKDK
RVFIRVDFNVPLDGKKITSNQRIVAALPTIKYVLEHHPRYVVLA
b
TA
4
5
FLAG
α-FLAG beads
c
RNCs-Sec22+
input
0
10
20
Pgk1-100
+ residues
kDa
1%
2%
4%
FLAG
35
*
Get4
3.2%
4.9%
6%
2.2%
Get4 [% of input]
1
1.8
2.2
0.4
Get4/RNCs [norm. ratio]
blot
66
Srp54
2.2%
2.7%
3.3%
0.3%
Srp54 [% of input]
1
1.4
1.8
0.08
Srp54/RNCs [norm. ratio]
25
RNCs (Rps9)
2.0%
1.7%
1.7%
3.5%
Rps9 [% of input]
wild type
d
kDa
input
total extract
66
Srp54
Get4
35
25
Rps9
blot
wild type
e
RNCs-Sec22
RNCs-Sec22+60
kDa
FLAG
35
Get4
25
Get5
14
RNCs (Rpl31)
blot
wild type
Δget3Δsgt2
wild type
Δget3Δsgt2
f
kDa
wild type
Δsrp54
Δget3
Δget3Δsgt2
Δget4Δget5
Δget3Δget4Δget5
66
Srp54
35
Sgt2
35
Get3
Get5
25
14
Rpl31
blot
g
TM
5
4
SRP
h
4
5
TA
Sgt2
i
kDa
total
cytosol
flow through
eluate
116
66
45
35
His6Get4
Get5
25
j
Get4/5
His6Get4/5
kDa
cyt
rib
cyt
rib
35
α-Get4
blot
Get4/5
His6Get4/5
kDa
cyt
rib
cyt
rib
25
α-Get5
k
RNCs-Sed5+60
RNCs-Bos1+60
His6Get4/Get5
kDa
FLAG
35
Sgt2
25
Get5
14
RNCs (Rpl31)
blot
wild type

Supplementary Figure 4. Binding of Get4/5 to RNCs. (a) Amino acid sequence of FLAG-tagged Sec22+60. The amino acid sequence of Sec22 is shown in dark gray with the tail anchor sequence highlighted in red. FLAG-tag (black background), C-terminal extension consisting of a 2-residue linker sequence (light gray background) and the 60 N-terminal residues of the cytosolic enzyme Pgk1 (gray background). FLAG-Sec22+60 contains 27 lysines, spread over the whole sequence (orange), which are available for crosslinking with BS^3. Untagged Sec22 constructs lack the FLAG-tag, constructs with 10-40 residues extensions (see Fig. 4a) contain correspondingly less Pgk1 residues. **(b)** Experimental set-up for FLAG-tag pull down reactions. RNCs were generated by *in vitro* translation of stop codon-less transcripts (see Methods). FLAG-tag pull down reactions were performed with RNCs exposing a nascent chain with an N-terminal FLAG-tag. As the experiments were performed under native conditions, factors bound to RNCs, such as Get4/5, SRP or Sgt2, were copurified. The scheme depicts RNCs carrying nascent N-terminally FLAG-tagged Sec22. FLAG-tag (black), tail anchor of Sec22 (TA, red), Get4 (brown) Get5 (light blue). For details see Methods and Supplementary Note 2. **(c)** Get4/5 binds to translating ribosomes in general. Pull down reactions were performed with RNCs carrying nascent Sec22, Sec22+10, Sec22+20 (Fig. 4a), or 100 N-terminal residues of the cytosolic enzyme Pgk1 (Pgk1-100). Nascent chains without a FLAG-tag were employed as controls. Aliquots of the translation reactions (1% - 4% input) were loaded onto the same gel. The amount of RNCs (Rps9), Get4/5 (Get4), and SRP (Srp54) in the input and FLAG-tag pull down reactions were assessed via immunoblotting followed by densitometric analysis. The intensities of the bands in the input were employed as a standard to calculate the fraction of Get4/5 and SRP, which was affinity purified via FLAG-tagged RNCs (gray numbers, % of input). Furthermore, intensities of the bands in the pull-down reactions were employed to determine the relative ratios Get4/Rps9 and Srp54/Rps9. The ratio obtained for RNCs-Sec22 was set to 1 (Get4/RNCs and Srp54/RNCs, normalized ratios). The normalized ratios allow to determine effects exerted by different nascent chains, which alter the recruitment of individual ribosome-bound factors. For example, the data show that the occupancy of RNCs-Pgk1 with Get4/5 was about 2-fold lower when compared to the occupancy of RNCs-Sec22 with Get4/5. This observation is consistent with a previous study (Fig. 4B in Zhang et al. 2016 [11]). A black asterisk labels a molecular weight marker band recognized by α-Get4. **(d)** The relative ratio of Get4/5 and SRP in total cell extract and *in vitro* translation reactions is similar. Translation reactions (see Methods), which were employed as input for pull down reactions were analyzed side by side with total yeast extract [14] via immunoblotting using antibodies directed against Get4, Srp54, and the ribosomal protein Rps9. **(e)** Get4/5 binding to RNCs is independent of Sgt2 and Get3. FLAG-tag pull down reactions were performed with RNCs carrying nascent Sec22 or Sec22+60 (Fig. 4a) in a wild type or Δ*get3*Δ*sgt2* translation extract (see panel f). Immunoblots were decorated with α-Get4, α-Get5, or α-Rpl31. **(f)** Yeast translation extracts employed in this study. Yeast translation extracts derived from wild type, Δ*srp54*, Δ*get3*, Δ*get3*Δ*sgt2*, Δ*get4*Δ*get5*, Δ*get3*Δ*get4*Δ*get5* were analyzed via immunoblotting with α-Srp54, α-Sgt2, α-Get5, α-Get3, and α-Rpl31. **(g)** Model of RNCs carrying nascent Dap2-60. The type 2 TM domain of Dap2-60 is within the ribosomal tunnel. Get4/5 and SRP are indicated. Ribosome (light gray), TM (magenta), Get4 (brown) Get5 (light blue), SRP (green). **(h)** Model depicting Sgt2-recruitment to RNCs via Get4/5. Sec22+60 exposes the TA outside of the ribosomal tunnel. Labeling as in panel b; TA (red), Sgt2 (dark blue). **(i)** Purified His_6Get4/5. His_6Get4 and Get5 were coexpressed in *E. coli* and the His_6Get4/5 complex was subsequently purified via Ni-NTA as described in the Methods section. **(j)** His_6Get4/5 binds efficiently to ribosomes. Ribosome binding of Get4/5 or His_6Get4/5 was analyzed as described in Fig. 1a using total cell extract derived from strains overexpressing either Get4/5 or His_6Get4/5. Immunoblots were decorated with α-Get4 or α-Get5 as indicated. Cytosolic fraction (cyt), ribosomal pellet (rib). **(k)** RNCs bound to nascent Sed5 or Bos1 recruit Sgt2 in a Get4/5-dependent manner. FLAG-tag pull down reactions were performed with RNCs-Sed5+60 and RNCs-Bos1+60 generated in a wild type translation extract. Purified His_6Get4/Get5 was added to a final concentration of 2 µM prior to the translation reaction as indicated (+). The relative amount of RNCs (Rpl31), ribosome-bound Get4/5 (Get5), and ribosome-bound Sgt2 was assessed by immunoblotting.

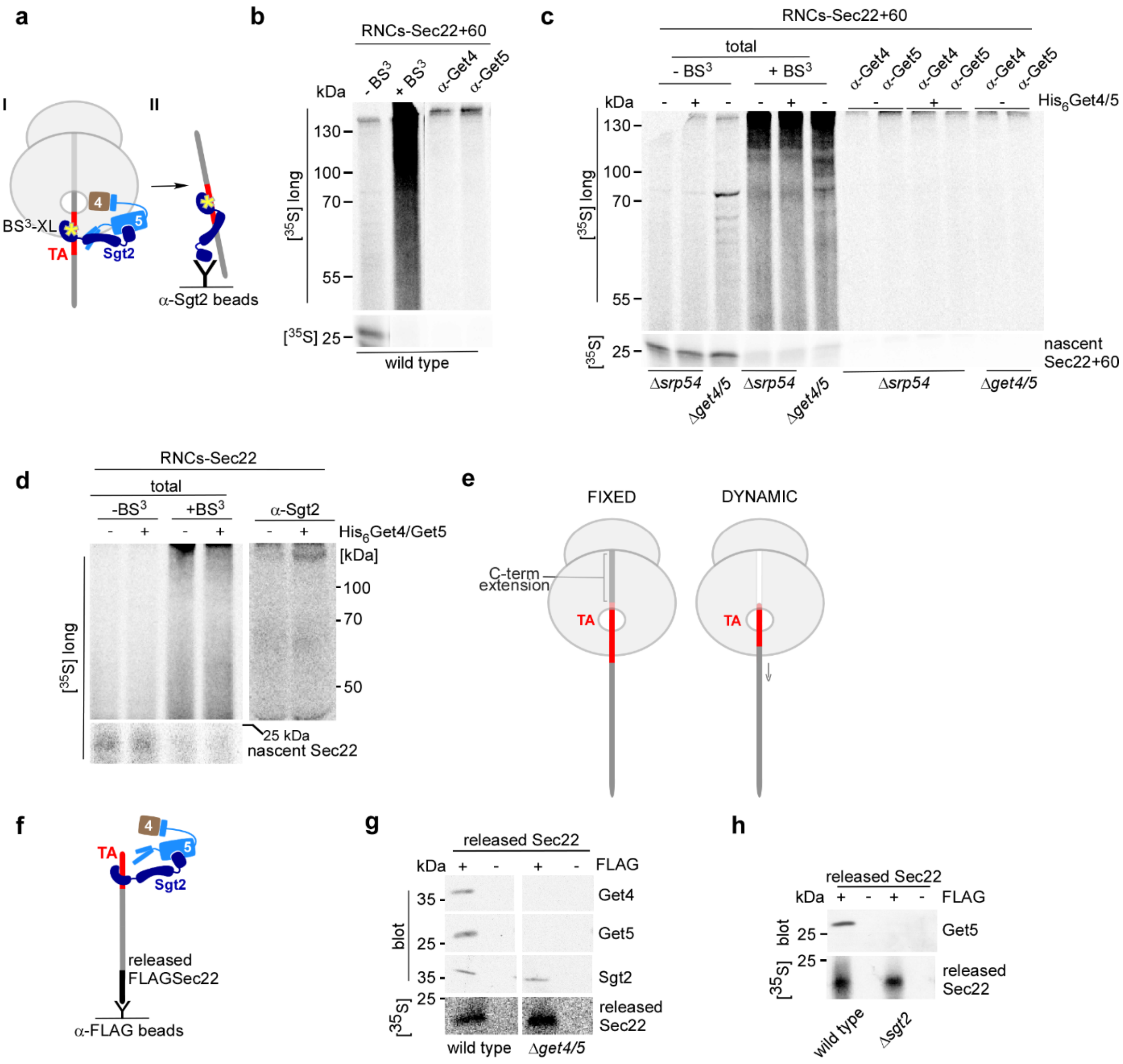

Supplementary Figure 5. Get4/5 is not in direct contact with a nascent TA sequence. (a) Experimental set up for the analysis of nascent chain contacts. (I) Isolated RNCs carrying [^{35}S]-labeled nascent chains were crosslinked to adjacent proteins using the homobifunctional amino-reactive crosslinker BS^3, spacer length 11.4 Å. (II) The formation of crosslink products between SRP, Sgt2, or Get4/5 and radiolabeled nascent chains was subsequently analyzed by affinity purification under denaturing conditions. For details see Methods. Shown is an RNC carrying nascent Sec22+60 with the (TA, red), Get4 (brown) Get5 (light blue), Sgt2 (dark blue), BS^3 crosslink product (BS^3-XL, yellow asterisk). **(b)** Get4/5 does not contact nascent Sec22+60 in wild type translation extract. Contacts of Get4 and Get5 to [^{35}S]-labeled Sec22+60 were probed via the crosslinking approach described in panel a. The autoradiograph shows total translation reactions before (- BS^3) or after (+ BS^3) crosslinking and crosslinked, affinity purified Get4 (α-Get4) or Get5 (α-Get5). The total represents 5% of the material applied to the affinity purification reactions. **(c)** Get4/5 does not contact nascent Sec22+60 in the absence of SRP or when the occupation of RNCs with Get4/5 is high. RNCs-Sec22+60 were generated in a Δ*srp54* or Δ*get4*Δ*get5* (control) translation extract and subsequently contacts of [^{35}S]-labelled Sec22+60 to Get4/5 were analyzed as described in panel b. Purified His$_6$Get4/Get5 was added to the translation reactions as indicated (+). **(d)** Sgt2 does not contact nascent Sec22. RNCs carrying radiolabeled Sec22 were generated in a wild type translation extract to which purified His$_6$Get4/Get5 was added to a final concentration of 2 μM as indicated. Contacts between nascent Sec22 and Sgt2 were analyzed via crosslinking as detailed in panel b using α-Sgt2 for the affinity purification of crosslink products. **(e)** Properties of ribosome-bound and released Sec22. FIXED: Ribosome-bound nascent chain complex generated by translation of a stop codon-less transcript coding for a C-terminally

extended version of Sec22. The C-terminal extension permanently fixes the TA sequence in a position accessible from outside the ribosomal tunnel. DYNAMIC: Sec22 generated by translation of a stop codon-containing transcript. Shown is the time point during ribosome-release at which the position of the TA sequence corresponds to the position of the fixed TA sequence. **(f)** Experimental set up for the detection of factors bound to ribosome-released Sec22. Radiolabeled released Sec22 was generated *in vitro* by translation of a transcript including a stop codon. Subsequently, ribosomes and Sec22 still associated with ribosomes were removed via ultracentrifugation, and FLAG-tag pull down reactions were performed with the ribosome-free supernatant. Binding of Get4 (brown) Get5 (light blue) and Sgt2 (dark blue) to affinity purified FLAG-Sec22 was analyzed via immunoblotting. The FLAG-tag is shown in black. For details see Methods. **(g)** Sgt2 is in a complex with released Sec22 in the absence of Get4/5. Released [^{35}S]-labeled FLAG-Sec22 was generated in a wild type or Δ*get4*Δ*get5* translation extract as described in panel f. FLAG-Sec22 was then isolated via FLAG-tag pull down and binding of Sgt2 (Sgt2) and Get4/5 (Get4 and Get5) was analyzed via immunoblotting. **(h)** Get4/5 is in a complex with released Sec22 only when Sgt2 is present. The experiment was performed as described in panel f and g after translation of FLAG-Sec22 in wild type or Δ*sgt2* translation extract. Binding of Get4/5 was analyzed via immunoblotting with α-Get5.

Supplementary Methods

Preparation of total protein extracts. Total protein samples were prepared as previously described [15]. In brief, 1 ml yeast cell cultures grown to an OD_{600} = 0.8 were collected and were resuspended in 1 ml 0.1 M NaOH. Samples were incubated on ice for 10 min, pellets were collected by centrifugation, and were subsequently resuspended and incubated at 95° C in SDS sample buffer.

Supplementary Tables

Supplementary Table 1. Diffusion times of Get4/5 alone and in complex with 80S ribosomes. [a] The remaining fraction of molecules with a shorter diffusion types is attributed to the free dye (typically 0.13-0.2). [b] The value was determined in the experiment with the Get4/5-Atto655 alone and fixed in fitting of other experiments.

complex	τ_{d1}, ms	fraction[a]	τ_{d2}, ms	fraction
Get4/5-Atto655	0.39 ± 0.01	0.83 ± 0.05	-	-
Get4/5-Atto655 + 80S	0.39[b]	0.19 ± 0.05	1.58 ± 0.05	0.67 ± 0.04

Supplementary Table 2. Normalized numbers of sequence reads derived from different types of RNA in wild type and Get4-HTP samples. PCR-amplified cDNA libraries (wild type 35 cycles; Get4-HTP 24 cycles) are shown in Supplementary Fig. 3f, pie charts in Supplementary Fig. 3h. The original CRAC data are available at the GEO database (GSE151664 [https://www.ncbi.nlm.nih.gov/geo/query/acc.cgi?acc=GSE151664]). Messenger RNA (mRNA), ribosomal RNA (rRNA), cryptic unstable transcript/stable uncharacterized transcripts/Xrn1-sensitive unstable transcripts (CUTs/SUTs/XUTs), small nucleolar RNA (snoRNA), non-coding RNAs (ncRNA), small nuclear RNA (snRNA), transfer RNA (tRNA).

	wild type	Get4-HTP
mRNA	415043	104657
rRNA	268046	106852
CUTs/SUTs/XUTs	10262	1662
snoRNA	13940	3786
ncRNA	778	1057
snRNA	1009	252
pseudogenes	2649	391
intergenic regions	135093	54167
tRNA	276485	799651

Supplementary Table 3. Strains and plasmids.

strains	genotype	reference
wild type BY4741a	*his3 leu2 met15 ura3*	[16]
wild type<br>MH272-3fα and MH272-3fa	*ura3 leu2 his3 trp1 ade2*	[17]
Get4-HTP$_{genom.}$	BY4741 *GET4*-HTP *HIS3*	this study
Get3-HTP$_{genom.}$	BY4741 *GET3*-HTP *HIS3*	this study
Sgt2-HTP$_{genom.}$	BY4741 *SGT2*-HTP *HIS3*	this study
Get5-HTP$_{genom.}$	BY4741 *GET5*-HTP *HIS3*	this study
wild type$_{+e.p.}$	BY4741 + pRS415	this study
HTP-Get5$_{plasm.}$	BY4741 *GET5::natNT2* + pRS415-HTP-GET5	this study
Get5-HTP$_{plasm.}$	BY4741 *GET5::natNT2* + pRS415-GET5-HTP	this study
Get4/5↑	MH272-3fα<br>YEplac555-Get4<br>YEplac181-Get5	this study
Get4/His$_6$Get5↑	MH272-3fα *get4::KANMX4 get5::KANMX4*<br>YEplac555-Get4<br>YEplac181-His$_6$Get5	this study
His$_6$Get4/Get5↑	MH272-3fα *get4::KANMX4 get5::KANMX4*<br>YEplac112-His$_6$Get4<br>YEplac181-Get5	this study
FLAGRpl35<br>Get4/His$_6$Get5	MH272-3fα *rpl35a::TRP1 rpl35b::LEU2*<br>YEplac444-Rpl35a-FLAG<br>pRS423-Get4<br>YEplac195-His$_6$Get5	this study
Δ*get4* + Get5↑	MH272-3fα *get4::KANMX4*<br>YEplac181-Get5	this study
get4Δget5	MH272-3fα *get4::KANMX4 get5::KANMX4*	[11]
Δ*get3*	MH272-3fα *get3::HIS3*	[11]
Δ*get3*Δ*get4*Δ*get5*	MH272-3fα *get3::HIS3 get4::KANMX4 get5::KANMX4*	this study
Δ*get3*Δ*sgt2*	MH272-3fα *sgt2::URA3 get3::HIS3*	this study

**plasmids**	**origin of replication and plasmid marker**	
pYEplac555-Get4	2μ, *ADE2*	this study
pYEplac181-Get5	2μ, *LEU2*	this study
pYEplac181-His$_6$-Get5	2μ, *LEU2*	this study
pYEplac112-His$_6$-Get4	2μ, *TRP1*	this study
pYEp444-Rpl35a-FLAG	2μ, *ADE2*	[18]
pRS423-Get4	2μ, *HIS3*	this study
pRS415-Get5-HTP	CEN, *LEU2*	this study
pRS415-HTP-Get5	CEN, *LEU2*	this study
pYEplac195-His$_6$Get5	2μ, *URA3*	this study
pETcoco2-Get5	Amp^R	this study
pET28a(+)-His$_6$Get4	Kan^R	this study
pETDuet-His$_6$Get5-Get4	Amp^R	this study
pSP65-Sec22	Amp^R	[11]

Supplementary Table 3. Strains and plasmids (continued).

pSP65-Sec22+60	Amp^R	this study
pSP65-FLAG-Sec22+60	Amp^R	this study
pSP65-FLAG-Sed5+60	Amp^R	this study
pSP65-Sed5+60	Amp^R	this study
pSP65-FLAG-Bos1+60	Amp^R	this study
pSP65-Bos1+60	Amp^R	this study
pSPUTK-Dap2α	Amp^R	[10]
pSPUTK-FLAG-Dap2α	Amp^R	[10]
pSPUTK-Dap2	Amp^R	[10]
pSPUTK-FLAG-Dap2	Amp^R	[10]

Supplementary Table 4. Primers and synthetic DNA.

Construct/Strain	sense 5‘ - 3‘	antisense 5‘ - 3‘
Sgt2-HTP	Sgt2-cHTP_fw<br>CTTTTTGGTGGAGCAGGTGCCCAATCTACAGATGAAACACCAGACAATGAGAACAAGCAATCCATGGAGCACCATC	Sgt2-cHTP_rev<br>GATAGAGCTATATTTGTATCTACATAACATGTATTGCATTAAAGGCTTATTTCAGTCCATACGACTCACTATAGGGCGA
Get4-HTP	Get4-cHTP_fw<br>CCTAAACAAACGAATTTCCTACAAGACATGATGTCGGGATTCCTGGGCGGATCGAAGTCCATGGAGCACCATC	Get4-cHTP_rev<br>CGTCATGGTCGTCAAGCCACCGCAAACATATTTATCTATTCCTTCGCAAATATGCTCTTTTACGACTCACTATAGGGCGA
Get5-HTP	Get5-cHTP_fw<br>GGCTGCGGTTAGGCAAGTCATGGAGCGTCTACAAAAAGGCTGGTCTCTGGCCAAATCCATGGAGCACCATC	Get5-cHTP_rev<br>CCGTGATAAAAAGCGCAACTGTGTAAAATAACAAGTATGTACGTACTAACTATACTAATCTACGACTCACTATAGGGCGA
Get3-HTP	Get3-cHTP_fw<br>CAAAGAATATAACCCTATTACTGATGGCAAAGTCATTTATGAGTTAGAAGATAAGGAATCCATGGAGCACCATC	Get3-cHTP_rev<br>CTATTTTTTCTATGGTTATATGTCGTATGTATCTATTTATGGTATTCAGGGGCTTTACGACTCACTATAGGGCGA
pYEplac555-Get4	Get4-Sac1-Fo<br>GCGTATGCAATGAGCTCATTGTAATGCACTTTGATGAAATTCCT	Get4-Pst1-Re<br>GCGTATGCTGCAGAAAAGATTCAGAAGTAGC
pYEplac181-Get5	Get5-Sph1-Fo<br>GGCCATTAAGGCATGCCTTTTGATGGCTATCGCACATC	Get5-Xba1-Re<br>GCAATTCGTATCTAGAAAGAACAAGATTAAGACGCA
pRS415-Get5-HTP	cHTP-get5 F<br>CCATACTCTAGAATGAGCACATCCGCCAGCGG	cHTP-get5 R<br>CTCCATGGATCCCGAGCCGCCGCCGCCTTTGGCCAGAGACCAGCCTT
pRS415-HTP-Get5	nHTP-get5 F<br>ACCCGGGGATCCATGAGCACATCCGCCAGCGG	nHTP-get5 R<br>ATCGATAAGCTTTTATTTGGCCAGAGACCAGCC
pETDuet-His$_6$Get5-Get4	pETDuet-His6Get5-Get4-fw<br>TAAGAAGGAGATATACATATGATGGTTCCTGCTGAA	pETDuet-His6Get5-Get4-rev<br>GGCCGATATCCAATTGAGATCTTTATCACTTCGATCC
	pETDuet-His6Get5-fw-Get4<br>CGCGTCGACGAAAATTTATATTTCCAATCTACTAGTGGATCCACC	pETDuet-His6Get5-rev-Get4<br>TATGCGGCCGCTTACATGACTCGAGTTA

Supplementary Table 4. Primers and synthetic DNA (continued).

pYEplac181-His$_6$-Get5<br>(parental plasmid: pYEplac181-Get5)	Get5-HindIII-F<br>ATGATTACGCCAAGCTTGCATG	Get5-StuI-R<br>GCTTATGTGGGAGGCCTTTTC
	synthetic template DNA encoding for His$_6$-Get5 (Bio Cat)<br>ATGATTACGCCAAGCTTGCATGcctttgatggctatcgcacatcTAGTAAGGGTTA<br>GATTGAGTATTGTCCTCTAATGTAACTTTTTCCTCTTATTCCTCTCCTCCC<br>CTTAGCTATTATATATCTTTTTTACCGAATCTTGAATTAGGAATAGTTTTA<br>AAATCAGGCCGGAGGCCGAGCTTGCGACAACTACGACCAAAAAAAACA<br>AAGGAAAAACGAAAAGAAGAAAACATGCACAGGAGAACATAGTTGGAGA<br>AAAGGGTTAGAACAGACCGATACAGAGATAAACTAGCGAAGAATAATAA<br>CTTTATACAAAATTAATCatgcatcatcatcatcatcatctgcaggtcgacggcATGAGCA<br>CATCCGCCAGCGGTCCAGAACACGAGTTCGTTAGTAAATTCTTGACCTT<br>AGCCACTTTAACTGAACCGAAGCTACCAAAAAGCTATACTAAACCTCTG<br>AAGGATGTTACCAATTTGGGTGTCCCGTTGCCTACTTTGAAGTATAAATA<br>TAAGCAGAATCGTGCCAAGAAGTTAAAACTGCACCAAGATCAGCAAGGT<br>CAAGACAACGCCGCCGTCCACTTAACCTTAAAGAAAATTCAAGCTCCCA<br>AGTTCTCCATCGAGCATGATTTCAGCCCATCAGATACTATCTTACAAATA<br>AAACAGCACTTGATCAGCGAGGAAAAGGCCTCCCACATAAGC	
pYEplac112-His$_6$-Get4	Get4-Xba1-F<br>GGACGACCAATCTAGAATTGTAAT<br>GCACTTTGATGAAATTC	Get4-Sac1-R<br>GGACGACCAAGAGCTCAGCTTGT<br>TATTATTATTTATGTC
	His8-Get4-Sac1-F<br>GGACGACCAAGAGCTCATGGGCA<br>GCCATCATCATCATCATCATCATC<br>ATGGCAGCGTTCCTGCTGAATCTA<br>ATGCTGTACAAGC	Get4-EcoRI-R<br>GGACGACCAAGAATTCAAAAGAT<br>TCAGAAGTAGCAAAGC
pRS423-Get4	Get4-Sal1-F<br>GCGTATGCTGTCGACATTGTAATG<br>CACTTTGATGAAATTC	Get4-EcoR1-R<br>GCGTATGCGAATTCAAAAGATTC<br>AGAAGTAGCAAAG
pETcoco2-Get5	GET5-Nsi1-fo<br>GGCTGCTGCTATGCATATGAGCAC<br>ATC CGCCAGCGGTC	GET5-Pac1-rev<br>CGCTGCTGCTTTAATTAATTATTT<br>GGC CAGAGACCAGCC
pET28a(+)-His$_6$Get4	Get4-Nde1-Fo<br>GCAGGTAATCATATGATGGTTCCT<br>GCTGAATCTAATGCT	Get4-EcoR1-Re<br>GCAGGATTAAGAATTCTCACTTC<br>GATCCGCCCAGGAATCC
pSP65-Sec22+60	Sec22-F<br>GGACGACCAAGAGCTCATGATAAA<br>GTCAACACTAATCTACAG	Sec22-R<br>GGACGACCAATCTAGATTTGAGG<br>AAGATCCACCAGAAGAGAAA
	PGK-Xba1-F<br>GGACGACCAATCTAGATCTTTATC<br>TTCAAAGTTGTCTG	PGK-80-Hind3-R<br>GGACGACCAAAAGCTTCTAAGCA<br>ACTGGAGCCAAAGAG
pSP65-FLAG-Sec22+60	FlagSec22-F<br>GGACGACCAAGAGCTCATGGACT<br>ACAAAGACGATGACGACAAAATAA<br>AGTCAACACTAATCTACAG	Sec22-R<br>GGACGACCAATCTAGATTTGAGG<br>AAGATCCACCAGAAGAGAAA-3`
	PGK-Xba1-F<br>GGACGACCAATCTAGATCTTTATC<br>TTCAAAGTTGTCTG	PGK-80-Hind3-R<br>GGACGACCAAAAGCTTCTAAGCA<br>ACTGGAGCCAAAGAG
pSP65-FLAG-Sed5+60	FLAG-Sed5-F<br>GAATACACGGAATTCGAGCTCATG<br>GACTACAAAGACGATGACGACAAA<br>AACATAAAGGATAGAACTTCAGAA<br>TTTCAACAAAGTGTG	Sed5-nonstop-R<br>TTGACTTTATTCTAGAATTGACTA<br>AAACCCAAATAACGAAAAATAC
	This PCR product was used to replace the FLAG-Sec22 fragment in pSP65-FLAG-Sec22+60	

Supplementary Table 4. Primers and synthetic DNA (continued).

pSP65-Sed5+60	Sed5-F<br>GTGCGACAGAGAATTCATCGATG	Sed5-nonstop-R<br>TTGACTTTATTCTAGAATTGACTA AAACCCAAATAACGAAAAATAC
pSP65-FLAG-Bos1+60	FLAG-Bos1-F<br>TACATAACTAGAGCTCATGGACTA CAAAG	Bos1+60-R<br>TGATTACGCCAAGCTTCTAAGCA AC
	synthetic template DNA encoding for His_6-Get5 (Eurofins Genomics)<br>TACATAACTAGAGCTCATGGACTACAAAGACGATGACGACAAAAACGCT CTTTACAACCATGCTGTGAAGCAAAAAAATCAACTACAACAAGAGTTGG CCAGGTTTGAAAAGAATTCTGTGACCGCCCCTATTTCTTTACAAGGGTC CATCTCTGCAACTCTGGTCTCACTGGAGAAAACAGTTAAGCAATATGCA GAACATTTAAACAGATATAAAGAAGATACTAATGCAGAGGAAATTGATCC TAAGTTCGCTAATCGACTAGCAACTTTAACACAGGATCTGCACGACTTTA CTGCCAAGTTTAAGGATTTAAAACAATCCTACAACGAAAATAATTCCAGA ACTCAGTTGTTTGGCTCAGGAGCATCGCATGTTATGGACTCCGATAACC CCTTTAGTACATCAGAGACCATCATGAATAAAAGGAACGTTGGTGGTGC GAGTGCAAATGGTAAAGAGGGCTCTAGCAACGGTGGGGGACTACCGTT GTACCAAGGGCTACAAAAGGAACAGTCTGTTTTCGAAAGGGGTAACGCT CAATTAGATTACATTCTAGAAATGGGCCAACAATCATTCGAAAATATAGT GGAACAAAACAAAATTTTATCCAAGGTACAAGATAGAATGTCAAATGGC CTAAGAACATTGGGTGTTTCGGAACAAACTATCACCTCTATCAATAAACG GGTGTTCAAAGATAAACTAGTCTTTTGGATCGCGTTAATTCTCTTGATCA TAGGTATTTATTATGTGTTGAAATGGTTAAGACTGCAGTCTTTATCTTCAA AGTTGTCTGTCCAAGATTTGGACTTGAAGGACAAGCGTGTCTTCATCAG AGTTGACTTCAACGTCCCATTGGACGGTAAGAAGATCACTTCTAACCAA AGAATTGTTGCTGCTTTGCCAACCATCAAGTACGTTTTGGAACACCACC CAAGATACGTTGTCTTGGCTTCTCACTTGGGTAGACCAAACGGTGAAAG AAACGAAAAATACTCTTTGGCTCCAGTTGCTTAGAAGCTTGGCGTAATC A	
pSP65-Bos1+60	Bos1-F<br>CACGGAATTCGAGCTCATGAACG CTCTTTACAACCATGCTG	Bos1+60-R<br>TGATTACGCCAAGCTTCTAAGCA AC
	synthetic template DNA as for pSP65-FLAG-Sed5+60	
Δ*get3*Δ*sgt2*	Sgt2-Nco1-F<br>GGCTTTAGTTCCATGGGAAATTAC AAAGGTCTTTTATGCTA	Sgt2-Cla1-R<br>GGCTTTAGTTATCGATACTGTCTT ACGCGAGAAGTTCTATA
**CRAC analysis**	**sense 5‘ - 3‘**	**application**
	Modified RA5 5' Adaptor<br>GTTCAGAGTTCTACAGTCCGACGA TCNNNNNAGC	Sequencing adaptor ligated during CRAC
	RA3 3' Adaptor<br>TGGAATTCTCGGGTGCCAAGG	Sequencing adaptor ligated during CRAC - note this oligo is 5' pre-adenylated (rApp) and has a blocked 3' dideoxycytosine (ddC) end
	RT-Primer<br>GCCTTGGCACCCGAGAATTCCA	Reverse transcription during CRAC
	RP1<br>AATGATACGGCGACCACCGAGAT CTACACGTTCAGAGTTCTACAGTC CGA	PCR amplification of cDNA libraries during CRAC - common forward primer
	Index primer_RPI2<br>CAAGCAGAAGACGGCATACGAGA TACATCGGTGACTGGAGTTCCTTG GCACCCGAGAATTCCA	PCR amplification of cDNA libraries during CRAC - reverse primer (Get4-HTP_1 sample)

Supplementary Table 4. Primers and synthetic DNA (continued).

	Index primer_RPI5 CAAGCAGAAGACGGCATACGAGA TCACTGTGTGACTGGAGTTCCTTG GCACCCGAGAATTCCA	PCR amplification of cDNA libraries during CRAC - reverse primer (WT sample)
	Index primer_RPI6 CAAGCAGAAGACGGCATACGAGA TATTGGCGTGACTGGAGTTCCTTG GCACCCGAGAATTCCA	PCR amplification of cDNA libraries during CRAC - reverse primer (Get4-HTP_2 sample)

Supplementary References

1. Sherman, F. Getting started with yeast. *Methods Enzymol.* **194,** 3-21 (1991).
2. Cherry, J. M. *et al.* Saccharomyces Genome Database: the genomics resource of budding yeast. *Nucleic Acids Res.* **40,** D700-705 (2012).
3. Raue, U., Oellerer, S. & Rospert, S. Association of protein biogenesis factors at the yeast ribosomal tunnel exit is affected by the translational status and nascent polypeptide sequence. *J. Biol. Chem.* **282,** 7809-7816 (2007).
4. Rome, M. E., Chio, U. S., Rao, M., Gristick, H. & Shan, S. O. Differential gradients of interaction affinities drive efficient targeting and recycling in the GET pathway. *Proc. Natl. Acad. Sci. U S A* **111,** E4929-4935 (2014).
5. Chartron, J. W., VanderVelde, D. G. & Clemons, W. M., Jr. Structures of the Sgt2/SGTA dimerization domain with the Get5/UBL4A UBL domain reveal an interaction that forms a conserved dynamic interface. *Cell Rep.* **2,** 1620-1632 (2012).
6. Jonikas, M. C. *et al.* Comprehensive characterization of genes required for protein folding in the endoplasmic reticulum. *Science* **323,** 1693-1697 (2009).
7. Wang, F., Brown, E. C., Mak, G., Zhuang, J. & Denic, V. A chaperone cascade sorts proteins for posttranslational membrane insertion into the endoplasmic reticulum. *Mol. Cell* **40,** 159-171 (2010).
8. Chio, U. S., Cho, H. & Shan, S. O. Mechanisms of Tail-Anchored Membrane Protein Targeting and Insertion. *Annu. Rev. Cell Dev. Biol.* **33,** 417-438 (2017).
9. Borgese, N., Coy-Vergara, J., Colombo, S. F. & Schwappach, B. The Ways of Tails: the GET Pathway and more. *Protein J.* **38,** 289-305 (2019).
10. Berndt, U., Oellerer, S., Zhang, Y., Johnson, A. E. & Rospert, S. A signal-anchor sequence stimulates signal recognition particle binding to ribosomes from inside the exit tunnel. *Proc. Natl. Acad. Sci. U S A* **106,** 1398-1403 (2009).
11. Zhang, Y. *et al.* Cotranslational intersection between the SRP and GET targeting pathways to the ER of Saccharomyces cerevisiae. *Mol. Cell. Biol.* **36,** 2374-2383 (2016).
12. Gasteiger, E. *et al.* in *The Proteomics Protocols Handbook* (ed Walker J.M.) (Humana Press, 2005).
13. Ben-Shem, A. *et al.* The structure of the eukaryotic ribosome at 3.0 A resolution. *Science* **334,** 1524-1529 (2011).
14. Yaffe, M. P. & Schatz, G. Two nuclear mutations that block mitochondrial protein import in yeast. *Proc. Natl. Acad. Sci. U S A* **81,** 4819-4823 (1984).
15. Kushnirov, V. V. Rapid and reliable protein extraction from yeast. *Yeast* **16,** 857-860 (2000).
16. Brachmann, C. B. *et al.* Designer deletion strains derived from Saccharomyces cerevisiae S288C: a useful set of strains and plasmids for PCR-mediated gene disruption and other applications. *Yeast* **14,** 115-132 (1998).
17. Heitman, J., Movva, N. R., Hiestand, P. C. & Hall, M. N. FK 506-binding protein proline rotamase is a target for the immunosuppressive agent FK 506 in Saccharomyces cerevisiae. *Proc. Natl. Acad. Sci. U S A* **88,** 1948-1952 (1991).
18. Gumiero, A. *et al.* Interaction of the cotranslational Hsp70 Ssb with ribosomal proteins and rRNA depends on its lid domain. *Nat. Commun.* **7,** 1-12 (2016).
