# Supplementary material for: Ribosome-bound Get4/5 facilitates the capture of tail-anchored proteins by Sgt2 in yeast
Source: Nat Commun. 2021 Feb 4;12:782. doi: 10.1038/s41467-021-20981-3 (PMC7862611; doi:10.1038/s41467-021-20981-3)
Supplement: Supplementary file 4 — Reporting Summary [file 41467_2021_20981_MOESM4_ESM.pdf]

nature research

Corresponding author(s): S. Rospert and B. Schwappach

Last updated by author(s): 21.12.2020

Reporting Summary

Nature Research wishes to improve the reproducibility of the work that we publish. This form provides structure for consistency and transparency in reporting. For further information on Nature Research policies, see our Editorial Policies and the Editorial Policy Checklist.

Statistics

For all statistical analyses, confirm that the following items are present in the figure legend, table legend, main text, or Methods section.

n/a	Confirmed	
☐	☒	The exact sample size (*n*) for each experimental group/condition, given as a discrete number and unit of measurement
☐	☒	A statement on whether measurements were taken from distinct samples or whether the same sample was measured repeatedly
☐	☒	The statistical test(s) used AND whether they are one- or two-sided<br>*Only common tests should be described solely by name; describe more complex techniques in the Methods section.*
☒	☐	A description of all covariates tested
☐	☒	A description of any assumptions or corrections, such as tests of normality and adjustment for multiple comparisons
☐	☒	A full description of the statistical parameters including central tendency (e.g. means) or other basic estimates (e.g. regression coefficient) AND variation (e.g. standard deviation) or associated estimates of uncertainty (e.g. confidence intervals)
☐	☒	For null hypothesis testing, the test statistic (e.g. *F*, *t*, *r*) with confidence intervals, effect sizes, degrees of freedom and *P* value noted<br>*Give P values as exact values whenever suitable.*
☒	☐	For Bayesian analysis, information on the choice of priors and Markov chain Monte Carlo settings
☒	☐	For hierarchical and complex designs, identification of the appropriate level for tests and full reporting of outcomes
☒	☐	Estimates of effect sizes (e.g. Cohen's *d*, Pearson's *r*), indicating how they were calculated

Our web collection on statistics for biologists contains articles on many of the points above.

Software and code

Policy information about availability of computer code

Data collection	No novel software was used
Data analysis	ImageJ 1.52a; PyMOL 2.2.0; Scaffold 4.9.0.; GraphPad Prism 6.07; Python 3.5.2<br>For analysis of CRAC data, Illumina sequence reads were trimmed and quality controlled using Flexbar (Dodt et al., 2012) and were mapped to the S. cerevisiae genome using Bowtie2 (Langmead and Salzberg, 2012). Heat maps were generated with Python (version 3.5.2).

For manuscripts utilizing custom algorithms or software that are central to the research but not yet described in published literature, software must be made available to editors and reviewers. We strongly encourage code deposition in a community repository (e.g. GitHub). See the Nature Research guidelines for submitting code & software for further information.

Data

Policy information about availability of data

All manuscripts must include a data availability statement. This statement should provide the following information, where applicable:
- Accession codes, unique identifiers, or web links for publicly available datasets
- A list of figures that have associated raw data
- A description of any restrictions on data availability

The CRAC datasets of Get4-HTP and the wild type yeast control are deposited in the Gene Expression Omnibus (GEO) database [http://www.bcbi.nlm.nih.gov/geo/] under the accession code GSE151664.The Uniprot database [https://www.uniprot.org] was used for screening peptides detected by mass spectrometry. The Saccharomyces Genome Database [https://www.yeastgenome.org] was used to derive gene sequences and as a source of yeast protein copy numbers. Source data and raw data are provided with this paper as a Source data file. Other data that support the findings of this study and biological materials are available from the corresponding authors on reasonable request.

Field-specific reporting

Please select the one below that is the best fit for your research. If you are not sure, read the appropriate sections before making your selection.

☒ Life sciences ☐ Behavioural & social sciences ☐ Ecological, evolutionary & environmental sciences

For a reference copy of the document with all sections, see nature.com/documents/nr-reporting-summary-flat.pdf

Life sciences study design

All studies must disclose on these points even when the disclosure is negative.

Sample size	Sample-size determination by statistical methods was not performed. Sample-size was adjusted for each set of experiments to reveal reproducibility and/or statistical significance of the data.
Data exclusions	No data were excluded from the analysis.
Replication	Reproducibility was verified by replications and statistical analysis of the data as indicated in the Figure Legends.
Randomization	Experiments were performed with randomly selected mutant yeast strains. The techniques employed average the behavior of the cells in culture and at the level of cell extracts. Samples in experiments were not randomized by a formal procedure. Instead, they were organized into test groups and appropriate controls, which were analyzed side-by-side and were subjected to the same treatments and procedures. No animal or human experimental subjects were involved.
Blinding	Experiments were performed with samples labelled according to experimental groups. However, these categories were assigned before any observation was made and all experimental procedures were applied equally to all samples and controls.

Reporting for specific materials, systems and methods

We require information from authors about some types of materials, experimental systems and methods used in many studies. Here, indicate whether each material, system or method listed is relevant to your study. If you are not sure if a list item applies to your research, read the appropriate section before selecting a response.

Materials & experimental systems

n/a	Involved in the study	
☐	☒	Antibodies
☒	☐	Eukaryotic cell lines
☒	☐	Palaeontology and archaeology
☒	☐	Animals and other organisms
☒	☐	Human research participants
☒	☐	Clinical data
☒	☐	Dual use research of concern

Methods

n/a	Involved in the study	
☒	☐	ChIP-seq
☒	☐	Flow cytometry
☒	☐	MRI-based neuroimaging

Antibodies

Antibodies used	Polyclonal antibodies directed against Srp54, Sgt2, Get4, Get5, Get3, Rpl4, Rpl24, Rpl35, Rpl31, Rpl26, Rps9, Pgk1, Kar2, and Sse1 (Eurogentec, Rospert lab antibody collection) and PAP (Sigma ID P1291) were raised in rabbit . PAP (Sigma ID P1291), α-FLAG (Sigma F3165), anti-His (Qiagen 34660), horseradish peroxidase-conjugated protein A (ThermoFischer Scientific 101023).
Validation	Antibodies directed against yeast proteins were validated via Western blotting. To that end, wild type and either deletions strains or (in case of essential genes) strains expressing a tagged-version of the respective protein were analyzed side-by-side. References for the Rospert lab antibodies are indicated in the Methods section. Anti-FLAG, anti-PAP and anti-His were validated by comparing Western blots of wild type yeast and yeast strains expressing FLAG- HTP- or His-tagged proteins.

April 2020
