# Supplementary material for: Ribosome-bound Get4/5 facilitates the capture of tail-anchored proteins by Sgt2 in yeast
Source: Nat Commun. 2021 Feb 4;12:782. doi: 10.1038/s41467-021-20981-3 (PMC7862611; doi:10.1038/s41467-021-20981-3)
Supplement: Supplementary file 5 — Description of Additional Supplementary Files [file 41467_2021_20981_MOESM5_ESM.docx]

Description of Additional Supplementary information

Title: Supplementary Dataset 1

Description: Ribosomal Proteins identified by mass spectrometry from crosslinked Ni-NTA purified His_6_Get5.
