# Supplementary material for: Ribosome-bound Get4/5 facilitates the capture of tail-anchored proteins by Sgt2 in yeast
Source: Nat Commun. 2021 Feb 4;12:782. doi: 10.1038/s41467-021-20981-3 (PMC7862611; doi:10.1038/s41467-021-20981-3)
Supplement: Supplementary file 2 — Peer Review File [file 41467_2021_20981_MOESM2_ESM.pdf]

REVIEWER COMMENTS

Reviewer #1 (Remarks to the Author):

The manuscript by Zhang et al illuminates the mechanism by which the “upstream” components of the GET pathway link ribosomal translation to proper ER targeting. Although the GET pathway has been known for over a decade now and despite multiple structural insights into its function this specific step in the chain has not been elucidated and as such, is an important missing link, now nicely added. The paper is deep and mechanistic, with enormous amounts of well-executed experiments, and well written. The discussion is also extensive and interesting. Most importantly it reconciles confusing and often contradicting data on the exact order by which events happen by demonstrating that Sgt2 is recruited to ribosomes translating TA proteins by direct binding of GET4/5 to the ribosome. As such, it provides a clear consolidated stream of events. This paper is an important addition to the literature on this pathway and I strongly support its publication. I have to say its one of the papers I have most enjoyed reading lately and I have very few suggestions or comments. Below are just a few small ones to make the paper more easily readable to the general cell biology community:
1. Figure 1a – it was not super clear to me that indeed there is a reduction in the presence of Get4 in the ribosome fraction when Get5 was absent. I think this panel would really benefit from quantitation (of a triplicate experiment?)
2. General comment – the text in the figures is really tiny and hard to read. Might be good to increase text size when possible
3. Figure 4a – you may want to add which protein it is and an explanation to the numbers above the schematic
4. Figure 6a – again a quantitation would be good to ascertain that the lack of Sgt2 in the ribosomal fraction of WT cells is not just a loading issue since it also seems like there is slightly more Sgt2 in the OE Get4/5 cells.
5. Figure 6 b/c – do you also have analysis of Sgt2 binding in this method in a “WT” control that can be added?
6. Figure 7 title – maybe add that it’s a model?

Reviewer #2 (Remarks to the Author):

In this very interesting manuscript, the authors characterize the involvement of the cytosolic duplex Get4/5 from the GET pathway in yeast, in the posttranslational delivery of tail anchored proteins. Using a variety of method including mass spectrometry and CRAC, they show that the duplex interacts closely with 80S ribosomes and binds directly via Get5. They also demonstrate that the Get4/Get5 duplex is required for Sgt2 recruitment.

However a few points would need to be addressed before publication :

The authors used a ribosome binding assay to show that Get5 is binding Rpl35 and Rpl26. It is stated that other antibodies and it would be useful to see the results in the figure to compare proteins stated as non-interactors with Rpl35 and Rpl26.

Then, RNA interactome of Get4 has been assessed by CRAC. Authors stated (l216, p5) that CRAC was not feasible on Get5. It would be better to write that Get5 did not express well with c-terminal HTP tag. Has other tag been tried? Did the author attempt to tag Get5 in N-terminal with an HTP tag since it apparently worked with 6 his tag for the MS experiment? Is 6His-Get5 sick?

A few more details would need to be stated in the manuscript : What is the percentage of reads binding to rRNA? A pie-chart (or similar) would be good to see clearly the distribution of reads among RNA classes for Get4 and Get5 to have a better idea of the proportion of total reads binding h46/h47.

Could Get3 (and Sgt2 if enough reads were sequenced) been shown on the rRNA alignment to show specificity of the peak.
A wider image of the radioactive blot would be better, along with the gel showing the cDNA library generated by PCR if possible. How many PCR cycles have been used?

Reviewer #3 (Remarks to the Author):

The earliest steps in the GET pathway – from the final stages of substrate synthesis to the moment of TMD capture by Sgt2 – remain poorly understood. The authors provide evidence for the hypothesis that Get5 docks with the ribosome exit tunnel to enable a novel pathway mechanism for facilitating TMD "transfer" from the ribosome to Sgt2. This hypothesis is appealing as a concept for how TMD capture by Sgt2 could be prioritized over TMD-mediated protein aggregation or promiscuous association with factors for TA protein mislocalization or degradation. As it stands, however, this work is preliminary and does not convincingly exclude a series of potential in vitro artifacts nor does it formally demonstrate a new role for Get5 in the pathway (besides its established one in the process of TA protein transfer from Sgt2 to Get3).

Major critiques:
1. The physical interaction studies between Get5 and the ribosome (as well as between Sgt2 and the ribosome, see minor critiques) are generally poorly controlled leaving the possibility that they are non-specific.
2. Even if a specific docking site for Get5 were to be present near the ribosomal exit tunnel, the physiological importance of such an interaction would also need to be established.

Suggestion:
The authors should take advantage published structural information about the Get4/5 complex and their ribosome binding assays in Fig. 1 (specifically the anisotropy/FCS) to screen for surgical Get5 mutants defective for ribosome binding (or Get4 mutants that influence Get4/5 complex docking to the ribosome). Mutant hits could then be "counter-selected" for those that have a wt-like ability to facilitate transfer from a pre-made Sgt2-substrate complex to Get3. Lastly, those mutants that have passed the double filter above, would be screened for defective Sgt2 substrate capture following protein synthesis termination (along the lines of Figure 5e, ie. with extracts derived from mutants endogenously expressed, as well as by doping in excess purified mutant complexes). Identification of mutants defined by these three criteria would convince me that this work has uncovered a novel step in the GET pathway, which would, of course, be of great interest to the field.

Minor critiques:

Figure 2:
It would be useful to see the total mass spec data presented in an unbiased way (e.g., as a GO term analysis of significant hits). For the all the other ribosome subunits identified that were not highlighted in Fig. 3e, I would still like to see them in the supplement collectively mapped onto the ribosome structure.

The size shift due to the presence of an additional FLAG epitope tag on Rpl35 in part d is not impressive and potentially troubling in light of the much more dramatic size shift of two analogous crosslinks to Ssb (a significantly bigger protein than Get5) shown in the work referenced for the reagent (Gumiero et al).

Figure 3:
Again, the total RNA profiling data should be presented in an unbiased way (e.g. Are ribosomal gene transcripts enriched?)

It is not clear why the h46/47 peak is specifically interesting when there are so many gross positional differences in the "background" with the wt shown for comparison. This background issue could be potentially remedied by repeating the analysis using any new Get4 mutants (fused to HTP) described above.

Figure 4:
This approach is in principle great but it needs more controls to be convincing. First, some RNCs might have undergone large/small subunit dissociation (catalyzed by extract factors) and led to the unwanted exposure of Sec22 TMD for Sgt2/Get3 recognition (which might also be intrinsically sensitive to the substrate C-terminal extensions used). Thus, it is important to repeat the experiment in sgt2/get3 double null extracts to avoid the potential for indirect interactions. An even better way of approaching this experimental challenge would be to pull-down on the ribosome (ideally with an epitope tagged, functional version of an RPS component) and then compare the extent of Get4/Srp54/Sgt2 co-association.

Part b/e: show blots for Get5 as this is the proximal ribosome docking component that is being argued for.

Part d/e: control for the specificity of the shown associations with RNCs by using previously defined Sgt2 mutants that don't interact with either the substrate or Get4/5.

Figure 5:
a-c) Studies of Sgt2-Sec22+60 are at best of dubious biological relevance. The authors should restrict their focus to any Sgt2 contacts with RNCs programmed with Sec22, as well as Sec22 released from ribosomes following synthesis.
e, f) The decrease of Sec22-Sgt interaction in the deltaGet4/5 background is one of the more promising results of the study. However, if Get4/5 were to enhance Sgt2's intrinsic TMD interaction ability (ie. even for substrate coming from a previous state that is not immediately ribosomal), the same effect would be seen in these assays. Thus, they should be repeated with any Get4/5 mutants identified that selectively disrupt ribosome exit tunnel docking.

Figure 6:
The analysis of the effects of overexpressed Get4/5 on Sgt2 migration in a polysome gradient does speak to the physiological relevance of their earlier findings. I understand the authors' belief that the ribosome-Sgt2 interaction is difficult to capture in WT cells without this trick but I would deemphasize it by putting it in the supplement and with additional controls to exclude Sgt2 binding to aggregates in the gradient e.g. How does Sgt2 lacking the C-terminal domain behave in these assays?) or What happens to Sgt2 migration when the gradients have been first treated to collapse polysomes (ie. with micrococcal nuclease) or dissociate ribosomes (ie. with EDTA)?

Reviewer #4 (Remarks to the Author):

The manuscript by Zhang et al. titled "Ribosome-bound Get4/5 facilitates the capture of tail-anchored proteins by Sgt2 in yeast" addresses the early steps of recognizing a tail-anchored membrane protein (TA protein) by a dedicated chaperone-like cascade, the GET system. The GET system, short for guided entry of TA proteins, is one of the eukaryotic protein targeting machineries delivering precursor proteins to the ER membrane and comprises at minimum six components (Get1-5 and Sgt2) in yeast.

However, as deemed necessary for TA proteins, the GET pathway mainly acts in a post-translational manner after completion of ribosomal translational and release of the synthesized cargo from the ribosomal peptidyl transferase center.

The authors show in a series of complex and versatile experiments that
- the duo of Get4 and Get5 mediates binding of the heterotetrameric Get4/5 complex to the ribosome,
- the Get4/5 complex binding occurs with nanomolar affinity near the ribosomal exit tunnel in non-translating ribosomes,
- the binding site near the ribosomal exit tunnel is shared (at least) between the Get4/5 complex and the classic signal recognition particle (SRP),
- binding of the Get4/5 complex is a prerequisite for Sgt2 recruitment to the nascent/released TA protein at the ribosomal exit tunnel.

While the manuscript appears professionally written and is bearing a logic continuation from one line of evidence to the next some minor and major aspects should be addressed before the manuscripts warrants publication. In particular, figures 4 and 6 raised some major questions.

Major points:
(1) Line 121ff: Was the experiment shown in Fig. 1a repeated? A quantification could help to estimate the pool of ribosome-associated and free Get4/5. Given the submicromolar affinity of Get4/5 for the 80S ribosome (line 166; Fig. 1b) and the 60x excess of ribosomes over Get4/5 complexes (300000 vs. 5000; line 384f.) the higher proportion of free Get4/5 should be discussed.
(2) Line 135f: Can the authors speculate why Get5 in absence of Get4 shows a much stronger ribosome association under low salt conditions (Fig. 1a)? Maybe this speculation can also be done with regard to the later finding that Get4 and Get5 bind via different interactions at the ribosomal surface (Figs. 2 and 3).
(3) Line 260ff:
- Was the experiment shown in Fig. 4b repeated?
- In contrast to the wild type (Fig. 4b, upper panel), why is so much more Get4 immunoprecipitated in the negative control without FLAG in absence of Srp54 (Fig. 4b, lower panel)? What happens to the protein levels of Get4/5/Sgt2 in the Δsrp54 strain?
- What happens to SRP recruitment in a ΔGet4 strain? More efficient or earlier SRP recruitment in absence of GET would further substantiate the competition between the SRP and GET pathway for substrates with a TM helix near the tunnel exit.
- Additionally, a bona fide bitopic (type II) transmembrane protein could be used as control to demonstrate SRP over Get4/5 preference.
(4) Line 286ff:
- In Fig. 4b lane 11 (RNCs-Sec22+60 construct) Get4 is not recruited to ribosome in the wild type strain and shows enhanced recruitment in the Δsrp54 strain. However, in the Get4 blot panel of Fig. 4e endogenous Get4 is found RNC associated (lane 1) and no substantial increase in recruitment of endogenous Get4 is seen in the Δsrp54 strain (lane 5).
- This discrepancy between Fig. 4b and 4e becomes even more prominent when adjusting for the Rps9 loading controls.
(5) Line 300ff:
- Why is the Sec22+60xSgt2 crosslink a double band? Is Sgt2 interacting in different conformations/positions with Sec22? Could Sgt2 act as the yeast equivalent of mammalian BAG6 and recruit E3 ligases for ubiquitination of captured substrates (cf. line 435 and the data by Hegde and colleagues)?
- Why is the Sec22+60xSgt2 crosslink appearing at different molecular weights in Fig. 5b (~60-80 kDa) and Fig. 5c (~90-110 kDa)?
(6) Line 306ff: Considering the circumstance that efficient Sgt2 interaction was demonstrated only using Sec22+60 (which is not an ideal representative of TA protein anymore) in conjunction with either SRP knockout or Get4/5 overexpression, the true biological relevance of the TA capture at the

ribosomal exit tunnel in a wild type setting is somewhat obscure.
(7) Line 336ff: Similar as in comment (6) the true nature and relevance of Sgt2 being recruited to translating ribosomes via Get4/5 in vivo (Fig. 6) is hard to grasp.
- The authors write (and cite their own published data) "previous analysis did not provide evidence for a ribosome-bound pool of Sgt2, it is possible that the amount of ribosome-bound Sgt2 was too low to be detected via immunoblotting".
- The sucrose gradient fractionations show only the two opposing scenarios Get4/5 overexpression versus Get4/5 knockout, but no wild type.
It appears that Sgt2 is recruited to translating ribosomes only by the non-physiological Get4/5 overexpression or deletion of SRP.
(8) Line 358f: "In this work, we show how newly synthesized tail anchored proteins are captured upon their emergence from the ribosomal tunnel." All the work was done using Sec22 (or C-terminal Sec22 extensions). Hence, the authors should restrict the statement to singular "We show how a newly synthesized tail anchored protein, Sec22, is captured upon its emergence from the ribosomal tunnel.""
(9) Line 979f: Given that Get4 seems to contact h46 and h47 inside the ribosomal tunnel the authors should also discuss the overlap with the NAC binding site in addition to the overlap with SRP (cf. Gamerdinger et al. 2019, doi:10.1016/j.molcel.2019.06.030). Based on this, how would the Sgt2 recruitment be altered in a ΔNAC strain?
(10) Line 1022: Why is the cross-reaction for endogenous Get4 mentioned for Fig. 4e not appearing in any other lane of the blot (e.g. lane 4 (wild type) or lane 5, 6, 8 (Δsrp54))?
(11) Line 1115: With the model for the capture of TM domains upon exit from the ribosomal tunnel (Fig. 7) the reader wonders how unique or universal is the C-terminal Sec22 TM domain compared to other tail-anchor sequences (cf. comment (8) and (6)).
(12) Line 1: Taking into consideration the data presented in Figs. 5 and 6 the title should more accurately reflect, that "The TA protein Sec22 can be captured by Sgt2 at the ribosomal tunnel exit upon overexpression of the GET mediator complex Get4/5".

Minor points:
(13) Line 268: Consider deleting "(see also Discussion)"
(14) Line 273: Consider deleting "(see Introduction)"
(15) Line 274: that position Sgt2 -> that positions Sgt2
(16) Line 275: "Pull down experiments" -> If not mistaken, Fig. 4b, d, e is based on α-FLAG M2 beads using an immobilized α-FLAG antibody and hence the authors should use the term immunoprecipitation instead of pull down.
(17) Line 328: The knockout of the Δget3 strain/translation extract should be shown via immunoblot (in comparison to a control) in the supplement or main manuscript.
(18) Line 332: The bars in Fig. 5f could be arranged in same order as the lanes in Fig. 5e.
(19) Line 426ff: Recent efforts from public data repositories provided a re-nomenclature of the mammalian TRC/GET components. Maybe the authors should adhere to this updated nomenclature throughout the manuscript.
(20) Line 917: While mathematically possible, it seems more sound to plot the two data points instead the s.e.m. (Fig. 1b).
(21) Line 940: α-Get4 (His6Get5-XL in b), α-Rpl26
(22) Line 941: (Rpl26-XL, Rpl35-XL in c)
(23) Line 945: Period at end of sentence is in bold.
(24) Line 1031: Delete sentence "Sgt2 binding to wild …"
(25) Line 1035: While mathematically possible, it isn't ideal to compare significance between groups with two data points. Given that more than two groups are compared and blotted an ANOVA analysis would be the better statistical test (Fig. 4f).
(26) Line 1080: Given that more than two groups are compared and blotted an ANOVA analysis would be the better statistical test (Fig. 5f).
(27) Line 1090: Rpl 31 (ribosomal marker) and α-Sse1 -> Rpl 31 (ribosomal marker), and α-Sse1

(28) Line 1098: were pooled and aliquots -> were pooled and equivalent (??) aliquots

REPLY TO REVIEWER COMMENTS

Reviewer #1 (Remarks to the Author):

The manuscript by Zhang et al illuminates the mechanism by which the “upstream” components of the GET pathway link ribosomal translation to proper ER targeting. Although the GET pathway has been known for over a decade now and despite multiple structural insights into its function this specific step in the chain has not been elucidated and as such, is an important missing link, now nicely added. The paper is deep and mechanistic, with enormous amounts of well-executed experiments, and well written. The discussion is also extensive and interesting. Most importantly it reconciles confusing and often contradicting data on the exact order by which events happen by demonstrating that Sgt2 is recruited to ribosomes translating TA proteins by direct binding of GET4/5 to the ribosome. As such, it provides a clear consolidated stream of events. This paper is an important addition to the literature on this pathway and I strongly support its publication. I have to say its one of the papers I have most enjoyed reading lately and I have very few suggestions or comments. Below are just a few small ones to make the paper more easily readable to the general cell biology community:

We are delighted that Reviewer #1 enjoyed the manuscript and thank him/her for the support and positive evaluation. We found his/her comments very helpful, and find that the changes and additions made based on these comments improve the manuscript significantly.

1. Figure 1a – it was not super clear to me that indeed there is a reduction in the presence of Get4 in the ribosome fraction when Get5 was absent. I think this panel would really benefit from quantitation (of a triplicate experiment?)

The experiment shown in Fig. 1a was performed in triplicate. The quantification of the results is now shown as **new Fig. 1b**. Please also refer to our reply to Reviewer #4, point 1.

2. General comment – the text in the figures is really tiny and hard to read. Might be good to increase text size when possible

We have increased the text size to at least ARIAL 8 in all Figures.

3. Figure 4a – you may want to add which protein it is and an explanation to the numbers above the schematic

We have improved the labelling of the schematic shown in Fig. 4a.

4. Figure 6a – again a quantitation would be good to ascertain that the lack of Sgt2 in the ribosomal fraction of WT cells is not just a loading issue since it also seems like there is slightly more Sgt2 in the OE Get4/5 cells.

We performed the experiment shown in Fig. 6a in triplicate and also added immunoblot data for Get5. We do not find a significant effect on the Sgt2 level when Get4/5 is overexpressed. We have further optimized the quality of the Sgt2 immunoblots (see also reply to Reviewer #4,

point 7). With this improved procedure, we are now able to detect Sgt2 also in the ribosomal fractions of the wild type strain under low salt conditions (Fig. 6a). This observation fits very well with our model that Sgt2 can be recruited to ribosomes *in vivo*. As before, ribosome-binding of Sgt2 is strongly enhanced when Get4/5 is overexpressed. The quantification of the data is now shown as **new Fig. 6b**.

5. Figure 6 b/c – do you also have analysis of Sgt2 binding in this method in a "WT" control that can be added?

Yes, we also performed ribosome profile analysis in the wild type strain. The data are now shown as **new Fig.6d**. Consistent with the analysis via the sucrose cushions (Fig. 6a), ribosome profile analysis reveals a small, but significant, fraction of Sgt2 in the polysome fractions.

6. Figure 7 title – maybe add that it's a model?

We added the word "model" to the title of Fig. 7

Reviewer #2 (Remarks to the Author):

In this very interesting manuscript, the authors characterize the involvement of the cytosolic duplex Get4/5 from the GET pathway in yeast, in the posttranslational delivery of tail anchored proteins. Using a variety of method including mass spectrometry and CRAC, they show that the duplex interacts closely with 80S ribosomes and binds directly via Get5. They also demonstrate that the Get4/Get5 duplex is required for Sgt2 recruitment.

However a few points would need to be addressed before publication:

The authors used a ribosome binding assay to show that Get5 is binding Rpl35 and Rpl26. It is stated that other antibodies and it would be useful to see the results in the figure to compare proteins stated as non-interactors with Rpl35 and Rpl26.

We now included the immunoblots of the crosslinking screen (RPP0/uL10, Asc1/RACK1, Rps3/uS3, Rps9/uS4, Rps20/uS10, α-Rpl4/uL4, Rpl17/uL22, Rpl31/eL31, α-Rpl25/uL23, α-Rpl16/uL13) in the **Source Data file** (tab "other antibodies for Get5XL"), which we uploaded for the information of Reviewer #2.

Then, RNA interactome of Get4 has been assessed by CRAC. Authors stated (l216, p5) that CRAC was not feasible on Get5. It would be better to write that Get5 did not express well with c-terminal HTP tag. Has other tag been tried? Did the author attempt to tag Get5 in N-terminal with an HTP tag since it apparently worked with 6 his tag for the MS experiment? Is 6His-Get5 sick?

We thank Reviewer #2 for this suggestion. Indeed, we had tried to generate a genomically tagged Get5-HTP strain more than once. In each of the attempts clones that had supposedly

integrated Get5-HTP into the genome grew poorly and expressed only little Get5-HTP (Supplementary Fig. 3a and 3b). We now generated yeast low copy plasmids (pRS415-Get5-HTP and pRS415-HTP-Get5) for the expression of N- as well as C-terminally HTP-tagged Get5. When these plasmids were introduced into a Δ*get5* strain, both Get5-HTP and HTP-Get5 showed an expression level slightly higher when compared to wild type Get5 (**new Supplementary Fig. 3c**), as expected upon expression from a low copy plasmid. Importantly, Get5-HTP and HTP-Get5 strains displayed wild type growth (**new Supplementary Fig. 3d**). These findings suggest that some unexpected genomic event had occurred upon integration of the HTP cassette at the 3'-end of the *GET5* coding sequence. We now performed CRAC analysis with Δ*get5* strains expressing either plasmid-borne HTP-Get5 or Get5-HTP. However, no crosslinking to cellular RNAs was detected (**new Supplementary Fig. 3e**) and therefore the samples were not further processed.

A few more details would need to be stated in the manuscript: What is the percentage of reads binding to rRNA? A pie-chart (or similar) would be good to see clearly the distribution of reads among RNA classes for Get4 and Get5 to have a better idea of the proportion of total reads binding h46/h47.

We now added **new Supplementary Table 3**, which lists reads of different RNA species in wild type and Get4-HTP. A pie-chart was also added as **new Supplementary Fig. 3h**.

Could Get3 (and Sgt2 if enough reads were sequenced) been shown on the rRNA alignment to show specificity of the peak.

As no specific crosslinking of Get3-HTP or Sgt2-HTP to cellular RNAs was observed (Fig. 3b), areas of the membrane were not excised for these samples and they were not further processed. The specificity of the Get4-HTP peak in 25S-h46/47 is demonstrated by comparison to the wild type control.

A wider image of the radioactive blot would be better, along with the gel showing the cDNA library generated by PCR if possible. How many PCR cycles have been used?

A wider image of the autoradiograph is now shown in Fig. 3b and the PCR amplified library is shown in **new Supplementary Fig. 3f**. Thirty-five PCR cycles were performed for the wild type, and 24 cycles for the Get4-HTP sample (**new Supplementary Fig. 3f**). Please note that the complete, uncropped images of all autoradiographs and immunoblots are shown in the **Source Data file**.

Reviewer #3 (Remarks to the Author):

The earliest steps in the GET pathway – from the final stages of substrate synthesis to the moment of TMD capture by Sgt2 – remain poorly understood. The authors provide evidence for the hypothesis that Get5 docks with the ribosome exit tunnel to enable a novel pathway mechanism for facilitating TMD "transfer" from the ribosome to Sgt2. This hypothesis is appealing as a concept for how TMD capture by Sgt2 could be prioritized over TMD-mediated protein aggregation or promiscuous association with factors for TA protein mislocalization or

degradation. As it stands, however, this work is preliminary and does not convincingly exclude a series of potential in vitro artifacts nor does it formally demonstrate a new role for Get5 in the pathway (besides its established one in the process of TA protein transfer from Sgt2 to Get3).

We agree with Reviewer #3 that it is currently only poorly understood how aggregation of C-terminal TMDs is prevented. We feel that this is a reason why the work presented in this study is of general interest as the findings provide pioneering insight into exactly this process.

We politely disagree, however, that the work is preliminary, as we provide ample evidence on the novel role of Get4/Get5 in the GET pathway. In our opinion, the major conclusions are supported by multiple, complementary, and well-controlled datasets.

Major critiques:

1. The physical interaction studies between Get5 and the ribosome (as well as between Sgt2 and the ribosome, see minor critiques) are generally poorly controlled leaving the possibility that they are non-specific.

It is unclear to us which of the multiple experiments revealing physical interaction between Get5 and the ribosome seem poorly controlled in the view of Reviewer #3.

In this work the ribosome binding of Get4/5 is shown by 7 different methods (method 8 provides indirect evidence for the ribosome binding of Get4/5), which are summarized in **Table R1**. We added example publications from our labs, as well as from other research groups, in which these, or similar methods, were applied to reveal physical interaction between proteins, or proteins and RNA, respectively.

Table R1. Presented evidence for physical interaction between Get4/5 and ribosomes.

	Method	Data	References
1	ribosome binding assay *(result: salt-sensitive binding of Get4/5, a commonly used criterion for the identification of ribosome-bound factors)*	Fig.1a and new Fig. 1b<br><br>Fig. 6a and new Fig. 6b	[1,2]
2	fluorescence anisotropy-based binding assay *(result: high affinity binding of Get4/5 to ribosomes Kd = 110 nM)*	Fig. 1c	[3-6]
3	fluorescence correlation spectroscopy (FCS) *(result: confirmation of ribosome binding, as analysis directly implies a very large complex, and high binding affinity*	Fig. 1d	[7]
4	crosslinking of Get4/5 to ribosomal proteins with a homobifunctional, amino-specific crosslinker; analysis with 2 independent methods: immunoblotting and mass spec *(result: Get5 is in direct contact with ribosomal proteins Rpl35 and Rpl26)*	Fig. 2b-d	[8,9]
5	crosslinking and analysis of cDNA (CRAC) *(result: Get4 contacts rRNA h46/47)*	Fig. 3b-d; Fig. S3	[10-12]
6	affinity purification of RNCs and associated factors via specific nascent chains (FLAG pull-down assay) (*result:*	Fig. 4 and Fig. S4	[13-15]

	Get4/5 binds to RNCs, binding is affected by the type of the nascent chain and by SRP, which possesses an overlapping ribosomal binding site)		
7	ribosome profile analysis, *"gold standard" for the identification of ribosome-bound factors* (*result: Get4/5 is bound to ribosomes and polysomes*)	Fig. 6c-f	16-19
8	nascent chain crosslinking (*result: Get4/5 does not crosslink to nascent chains, however, Sgt2 forms crosslinks with nascent chains only in the presence of Get4/5*)	Fig. 5 and Fig. S5	15,20,21

2. Even if a specific docking site for Get5 were to be present near the ribosomal exit tunnel, the physiological importance of such an interaction would also need to be established.

The crosslinking experiments (Fig. 2 and Fig. S2) reveal that Get5 binds close to the ribosomal exit tunnel. While this is an important finding by itself, the data presented in the manuscript also provide evidence of a physiological consequence of Get4/5 binding close to the tunnel exit, namely, recruitment of Sgt2 (Fig. 5 and Fig. 6). We show that Get4/5-dependent recruitment of Sgt2 to ribosomes enhances the capture of a released TA protein by Sgt2 (Fig. 5e and 5f). There may be additional roles of ribosome-bound Get4/5, however, exploring these is beyond the scope of this study. In the revised manuscript, we show that Sgt2 is recruited to polysomes in wild type yeast cells in a Get4/5-dependent manner (**new Fig. 6a, 6b, and 6d**, see also our reply to Reviewer #4, point 7). This finding further supports the model that the physiological role of ribosome-bound of Get4/5 is the recruitment of Sgt2 into the proximity of the ribosomal tunnel exit.

Suggestion:
The authors should take advantage published structural information about the Get4/5 complex and their ribosome binding assays in Fig. 1 (specifically the anisotropy/FCS) to screen for surgical Get5 mutants defective for ribosome binding (or Get4 mutants that influence Get4/5 complex docking to the ribosome). Mutant hits could then be “counter-selected” for those that have a wt-like ability to facilitate transfer from a pre-made Sgt2-substrate complex to Get3. Lastly, those mutants that have passed the double filter above, would be screened for defective Sgt2 substrate capture following protein synthesis termination (along the lines of Figure 5e, ie. with extracts derived from mutants endogenously expressed, as well as by doping in excess purified mutant complexes). Identification of mutants defined by these three criteria would convince me that this work has uncovered a novel step in the GET pathway, which would, of course, be of great interest to the field.

Importantly, we disagree that the identification and characterization of Get4/5 mutants that do not bind to the ribosome is required to establish that Get4/5 binds to ribosomes. As outlined above, we have collected a wealth of evidence using multiple well-established approaches to show that Get4/5 binds to ribosomes (**Table R1**). We would like to point out that finding “surgical mutants”, as suggested by the reviewer, may take years. For example, Ssb was first shown to bind to ribosomes in 1992. At that time, the Craig lab applied only a single method, ribosome profile analysis, which is regarded as the gold standard to establish ribosome-binding of a cytosolic protein [16]. The finding that Ssb binds to ribosomes, at the time, was entirely unexpected. Well-defined mutants of Ssb, which no longer interact with ribosomes, were identified only in 2016 [2,9]. Ribosome-binding of NAC was discovered in 1994 [22] and the first

non-binding mutant was described in 2006 [23]. Eukaryotic SRP was discovered by Walter and Blobel in 1980 [24]. Only very recently, Wild *et al.* elegantly dissected ribosome interactions of human SRP with the ribosome *in vitro* [25].

The experiments suggested by Reviewer #3 represent a wide-ranging project by itself and are beyond the scope of this study.

Specifically, Reviewer 3 suggests a 4-step approach: i) design of "surgical" Get4/5 mutants, which, based on currently available structural information, do not bind to the ribosome; ii) expression and purification of the designed Get4/5 mutant library and *in vitro* characterization of the binding properties of each mutant with anisotropy and/or FCS; iii) selection of those Get4/5 mutants, which do not bind to the ribosome, but display wild type activity with respect to the *in vitro* transfer of released TA proteins from Sgt2 to Get3 ; iv) Get4/5 mutants identified in steps i-iii should then be analyzed with in the *in vitro* translation system as shown in Fig. 5e.

We feel that this approach is currently not feasible for the following reasons.

We identify the general region where Get4/Get5 contacts the ribosome in this work. As this is a new discovery, a structure of the Get4/Get5 complex bound to the ribosome is not yet available. However, such structural insight would be required to design the mutants suggested by the referee as the first step of the project (step i). The existing structures of isolated Get4/5, Get3/4/5, and Sgt2/Get4/5 complexes/subcomplexes are not suitable to this purpose. However, based on the available structural information of the Get4/5 complex and the work presented in this study, we are optimistic that experts in ribosome cryo-EM or ribosome crystallography will initiate the elucidation of such structures, which will enable the research community in the future to generate mutants of Get4/5, which do not interact with the ribosome.

We would also like to point out that mutants within Get4/5, which no longer bind to the ribosome, but still perform like wild type Get4/5 with respect to the transfer of TA proteins from Sgt2 to Get3 may not exist. It is possible that the domain(s) of Get4/5, which interact with the ribosome also affect binding to Sgt2 and/or Get3. Moreover, capture of a TA protein by Sgt2 after ribosome release in the absence of Get4/5 is significantly reduced, however, is still 60% of the wild type (Fig. 5e and 5f). We discuss this observation, which is likely due to the previously reported chaperoning of released TA proteins by the cytosolic Hsp70 homolog Ssa [26]. In case Get4/5 mutants identified in step i-iii would display residual binding activity, below the detection limit of anisotropy/FCS, capture by Sgt2 might well be less affected when compared to the complete absence of Get4/5. We feel that based on the approach suggested by Reviewer #3 it might be hard to draw a clear conclusion with respect to the requirement of Get4/5 for the initial capture of TA proteins by Sgt2.

Minor critiques:

Figure 2:

It would be useful to see the total mass spec data presented in an unbiased way (e.g., as a GO term analysis of significant hits). For the all the other ribosome subunits identified that were not highlighted in Fig. 3e, I would still like to see them in the supplement collectively mapped onto the ribosome structure.

The total mass spec data including GO term analysis (GO biological process, cellular compartment or molecular function were analyzed against *S. cerevisiae* using either (i) PANTHER (http://www.pantherdb.org/) or (ii) GOrilla (http://cbl-gorilla.cs.technion.ac.il/) are now included in the **Source Data file**. We have defined inclusion criteria in the Results section: *(i) the analysis was confined to core ribosomal proteins (RPs); ii) only those RPs were selected for which the total spectral counts of His$_6$Get5xRP was at least 1.4-fold increased compared to the Get5xRP control; and iii) the p-value was ≤ 0.01).* All ribosomal proteins which met these criteria are mapped to the ribosome and are shown in Fig. 3e and Supplementary Fig. 3i.

The size shift due to the presence of an additional FLAG epitope tag on Rpl35 in part d is not impressive and potentially troubling in light of the much more dramatic size shift of two analogous crosslinks to Ssb (a significantly bigger protein than Get5) shown in the work referenced for the reagent (Gumiero et al).

In our experience, one cannot predict migration of crosslink products on SDS-PAGE. They often migrate different from the calculated molecular mass because they are "branched" (see also our reply to Reviewer #4, point 5). We would like to point out that the size shift between the FLAGRpl35-XL and Rpl35-XL can only be explained by the molecular mass difference between untagged Rpl35 and FLAG-tagged Rpl35 (Fig. 2d). Even more importantly, the Rpl35-His$_6$Get5 as well as the FLAGRpl35-His$_6$Get5 crosslinks are recognized by α-Rpl35, but *only* the FLAGRpl35-His$_6$Get5 crosslink is recognized by α-FLAG. This controls for the possibility that α-Rpl35 cross-reacts with a contamination copurified in the Ni-NTA purified material (Fig. 2d).

Figure 3:

Again, the total RNA profiling data should be presented in an unbiased way (e.g. Are ribosomal gene transcripts enriched?)

The original CRAC data were uploaded to the GEO database (accession number GSE151664). We now included **new Supplementary Table 3**, which lists the normalized numbers of sequence reads derived from different types of RNA in the wild type and Get4-HTP samples. Moreover, we included a pie-chart, which indicates crosslinking to different RNA species in wild type and Get4-HTP samples (**new Supplementary Fig. 3h**). The CRAC experiment and analysis of the CRAC data is now also more precisely described in the **new Results section *Get4 contacts helix 46/47 of the 25S rRNA.***

It is not clear why the h46/47 peak is specifically interesting when there are so many gross positional differences in the “background” with the wt shown for comparison. This background issue could be potentially remedied by repeating the analysis using any new Get4 mutants (fused to HTP) described above.

We apologize that this experimental detail was not clear in the original version of the manuscript. As explained below, the peak heights in the wild type control and Get4-HTP samples are not directly comparable. Specific crosslinking to cellular RNAs is detected in the Get4-HTP sample, whereas only trace amounts of non-specific background RNA are co-purified in the wild type control (Fig. 3b, autoradiography). To generate cDNA libraries of sufficient concentration for sequencing, it was therefore necessary to amplify the wild type control sample much more than the Get4-HTP sample (thirty-five PCR cycles for wild type

compared to 24 PCR cycles for Get4-HTP). This information is now included in the methods section and the PCR libraries are shown in **new Supplementary Fig. 3f**. Due to the increased numbers of PCR cycles, the peak heights in the wild type control are over-estimates, when compared to the peak heights in the Get4-HTP sample. The procedure, however, allows to determine background peak positions in the wild type sample, which serve as important controls for the specificity of crosslinking. Importantly, the peak in h46/47 is observed only in the Get4-HTP sample, but not in the over-amplified wild type sample, indicating that h46/47 is a specific Get4 crosslinking site. This point is now highlighted in the revised Results section. As the identification of Get4 mutants that do not interact with the ribosome is beyond the scope of this study (see point 2), such mutants were not investigated by CRAC analysis.

Figure 4:

This approach is in principle great but it needs more controls to be convincing. First, some RNCs might have undergone large/small subunit dissociation (catalyzed by extract factors) and led to the unwanted exposure of Sec22 TMD for Sgt2/Get3 recognition (which might also be intrinsically sensitive to the substrate C-terminal extensions used). Thus, it is important to repeat the experiment in sgt2/get3 double null extracts to avoid the potential for indirect interactions. An even better way of approaching this experimental challenge would be to pull-down on the ribosome (ideally with an epitope tagged, functional version of an RPS component) and then compare the extent of Get4/Srp54/Sgt2 co-association.

We are happy to hear that Reviewer #4 likes the approach applied in Fig. 4. The data shown in Fig. 4 represent an important piece of evidence for the conclusion that Get4/5 binds to ribosomes/RNCs, competes with SRP for binding, and recruits Sgt2.

Preliminary remark: During the translation reaction a minor pool of nascent chains may be released from ribosomes, even though the mRNA does not contain a stop codon (**Fig. R1** taken from Raue et al., Supplements, Fig. 1a[13]). To remove this (minor) fraction of released chains, we separate RNCs from released chains by ultracentrifugation prior to each FLAG pull-down experiment. We describe this procedure in the Methods Section.

Raue *et al.* 2007:

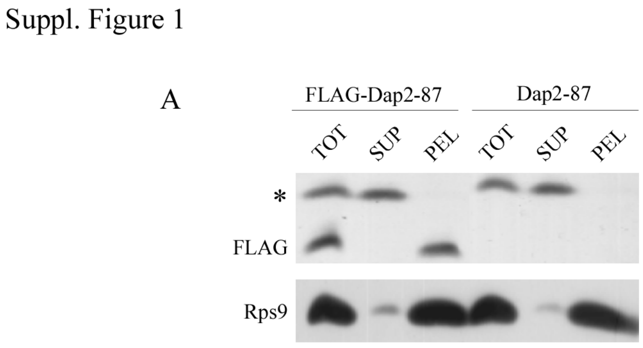

Figure R1. Properties of RNCs. FLAG-tagged nascent polypeptides are quantitatively bound to ribosomes. After a translation reaction primed with FLAG-tagged Dap2 (FLAG-Dap2-87) or untagged Dap2 (Dap2-87) RNCs were isolated via a low salt (150 mM KAcetate) sucrose cushion (20). Aliquots of the total reaction (TOT), the supernatant after centrifugation (SUP), and the ribosomal pellet (PEL) were separated on 10% TRIS-Tricine gels followed by immunoblotting. Localization of the nascent polypeptide was determined using a FLAG-tag antibody (FLAG). An unspecific cross reaction is indicated with an asterisk. Ribosomes were detected using an antibody directed against Rps9.[13]

In addition, we performed the control suggested by the Reviewer, which shows that Get4/5 binds to RNCs carrying a FLAG-tagged nascent chain rather than to released FLAG-tagged chains. We generated the Δ*get3*Δ*sgt2* strain and prepared a translation extract from this strain (**new Supplementary Fig. 4f**). Using this Get3/Sgt2-free translation extract we repeated key experiments. The data are shown in **new Supplementary Fig. 4e**. The results reveal that Get4/5 binds to RNCs even when Sgt2 and Get3 are absent from the reaction. We included **new Supplementary Result 2** in the Supplementary Results Section, which summarizes the findings of this control experiment.

Part b/e: show blots for Get5 as this is the proximal ribosome docking component that is being argued for.

We have now analyzed FLAG pull-down experiments shown in **new Fig. 4d** and **new Fig. 4e** (previously Fig. 4b), **new Fig. 4h**, and **new Fig. 6a** with Get4 as well as Get5 antibodies. The control reveals, as expected, Get4/5 is a stable complex.

<u>Side note.</u> Many of the ribosome-bound factors possess more than one subunit. If the complexes are stable, we normally use only one of the subunits for detection. This is the case for Get4/5, the heterodimers NAC and RAC, and the multi-subunit complex SRP. In this study we focus on Get4/5, Sgt2, and SRP. Please note that the amount of translation reaction applied to FLAG pull-down experiments cannot be increased, because this leads to strongly enhanced unspecific binding in the minus FLAG samples. Unfortunately, the molecular mass of the GET components does not allow for a parallel analysis of all components in one single experiment. Because Sgt2 and Get4 migrate closely together we often probe for Get5 when we want to analyze Sgt2 in parallel. Rps9 can be used as an RNC marker in combination with Get4 but migrates close to Get5. For this reason, we employ Rpl31 (13 kDa) as an RNC marker when we want to analyse Get5.

Part d/e: control for the specificity of the shown associations with RNCs by using previously defined Sgt2 mutants that don't interact with either the substrate or Get4/5.

The identification and/or characterization of Sgt2 mutants is beyond the scope of this study. The specificity of Sgt2 association with RNCs is shown by i) the minus FLAG-tag control, which shows that Sgt2 is bound to RNCs carrying FLAG-tagged nascent, but not untagged chains (Fig. 4 and Supplementary Fig. 4). This control excludes problems connected to aggregation, sticking to tube walls etc., ii) the specific effect exerted by Get4/5 on the recruitment of Sgt2 to RNCs (see Fig. 4g-i, Supplementary Fig. 4i, and Fig. 6a-f), iii) the affect that SRP exerts with respect to Get4/5, and concomitantly Sgt2, binding to RNCs (Fig. 4h and 4i).

Figure 5:

a-c) Studies of Sgt2-Sec22+60 are at best of dubious biological relevance. The authors should restrict their focus to any Sgt2 contacts with RNCs programmed with Sec22, as well as Sec22 released from ribosomes following synthesis.

In our view crosslinking experiments with RNCs-Sec22+60 turned out to be an important tool to obtain further insight into the early steps of the GET pathway. One important finding was that, if ribosome-bound and at the same time exposed outside of the ribosomal tunnel, a TA sequence preferentially binds to SRP when compared to Sgt2 (Fig.5b). We clearly state the *pros* and *cons* of this approach. "*In vivo,* TA sequences do not exit the ribosomal tunnel during ongoing translation, and emerge only after translation termination (**Supplementary Fig. 5e**)."

e, f) The decrease of Sec22-Sgt interaction in the deltaGet4/5 background is one of the more promising results of the study. However, if Get4/5 were to enhance Sgt2's intrinsic TMD interaction ability (ie. even for substrate coming from a previous state that is not immediately ribosomal), the same effect would be seen in these assays. Thus, they should be repeated with any Get4/5 mutants identified that selectively disrupt ribosome exit tunnel docking.

As the identification of Get4/5 mutants that do not interact with the ribosome is beyond the scope of this study (see above), these experiments were not performed.

Figure 6:

The analysis of the effects of overexpressed Get4/5 on Sgt2 migration in a polysome gradient does speak to the physiological relevance of their earlier findings. I understand the authors' belief that the ribosome-Sgt2 interaction is difficult to capture in WT cells without this trick but I would deemphasize it by putting it in the supplement and with additional controls to exclude Sgt2 binding to aggregates in the gradient e.g. How does Sgt2 lacking the C-terminal domain behave in these assays?)[a)] or What happens to Sgt2 migration when the gradients have been first treated to collapse polysomes (ie. with micrococcal nuclease) or dissociate ribosomes (ie. with EDTA)? [b)]

We improved detection of Sgt2 in the ribosome profile analysis (see also reply to Reviewer #4, point 7) and can now detect Sgt2 in pooled polysome fractions of a wild type strain (**new Fig. 6d**). We thus show the combined *in vivo* results in the main part of the manuscript. We feel that these data are an important piece of evidence for the conclusion that ribosome-bound Get4/5 and Sgt2 are physiologically important.

a) The generation, characterization, and analysis of Sgt2 or Get4/5 mutants is beyond the scope of this study.

b)

Preliminary remark: In all ribosome profiles we tested whether Sgt2 (and other ribosome-bound components) in the polysome fractions represent aggregates. For that purpose, we have analyzed the pellet at the bottom of the sucrose gradient (pel) for the presence of Sgt2, Get5, or Srp54 (Fig. 6c-f). None of the analyzed proteins formed aggregates during ribosome profile analysis. The method is now described in a **new paragraph of the Methods Section entitled *Ribosome profile analysis***.

We have also performed ribosome profiles of the Get4/5 overexpression strain +/- RNase treatment (**Fig. R2**). The experiments show that Sgt2 is bound to polysome fractions 9-17 in the untreated extract, but is no longer detected in fractions 9-17 after RNase treatment (**Fig. R2**). This confirms that Sgt2 is associated with polysomes rather than forming aggregates. Please note that fractions 9-17 were pooled prior to immunoblotting as detailed in the Methods section. The method ensures that all polysome-containing fractions of the untreated sample end up in a single lane of the immunoblot (lane 9-17). Even then, the Sgt2 band is weak, reflecting that a significant but small amount of Sgt2 is bound to polysomes (lane 9-17, -RNase). After RNase A treatment, ribosomes collapse into an 80S peak, however, the 80S peak is distributed over 3 major fractions (lanes 6-8, + RNase). Thus, the minor fraction of ribosome-bound Sgt2 will also be distributed over fraction 6-8, which means that it drops below the detection limit. Moreover, fractions 6-8 containing 80S ribosomes in the RNase treated sample are right adjacent to the cytosolic fractions 1-5, which makes is difficult to distinguish between the large cytosolic fraction of Sgt2 and the minor fraction of Sgt2 bound to RNase-treated 80S ribosomes. For these reasons, we prefer not including RNase-treated profiles in the manuscript, however, the data shown in **Fig. R2** validate our conclusions.

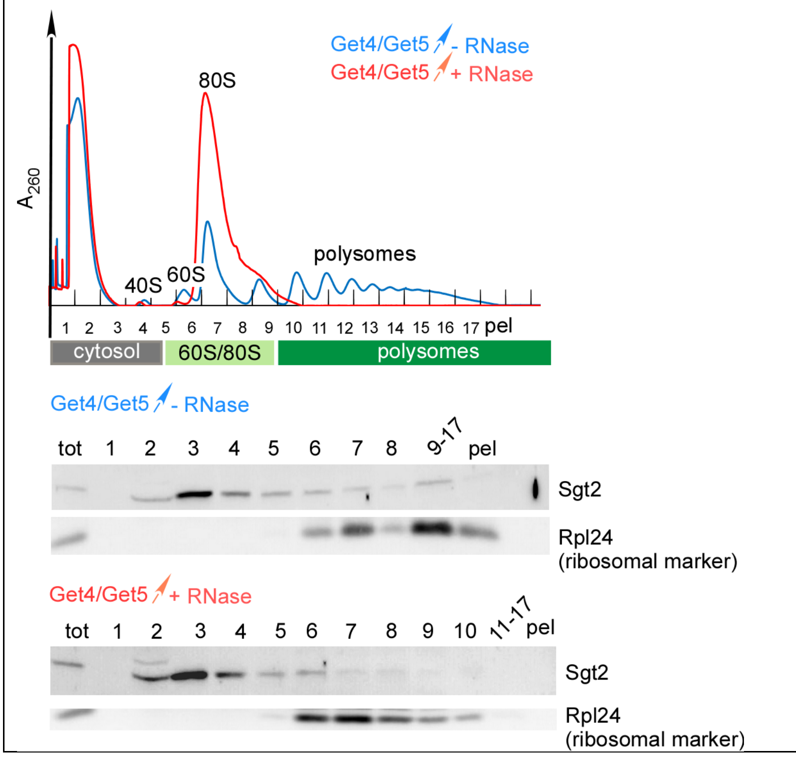

Figure R2. Sgt2 localization in the high-density fractions of a sucrose gradient depends on intact polysomes. Glass beads extract was treated with 0.25 mg/ml RNase A) for 15 min on ice as indicated. The major polysome-containing fractions (9-17, - RNase) or fractions devoid of ribosomes (11-17 + RNase) were pooled prior to analysis. The total (tot) corresponds to 5% of the cytosolic extract loaded onto the gradient. The pellet at the bottom of the gradient tube (pel) was dissolved in sample buffer and included in the analysis. For more information see Methods section.

Reviewer #4 (Remarks to the Author):

The manuscript by Zhang et al. titled “Ribosome-bound Get4/5 facilitates the capture of tail-anchored proteins by Sgt2 in yeast” addresses the early steps of recognizing a tail-anchored membrane protein (TA protein) by a dedicated chaperone-like cascade, the GET system. The GET system, short for guided entry of TA proteins, is one of the eukaryotic protein targeting machineries delivering precursor proteins to the ER membrane and comprises at minimum six components (Get1-5 and Sgt2) in yeast. However, as deemed necessary for TA proteins, the GET pathway mainly acts in a post-translational manner after completion of ribosomal translational and release of the synthesized cargo from the ribosomal peptidyl transferase center.

The authors show in a series of complex and versatile experiments that

- the duo of Get4 and Get5 mediates binding of the heterotetrametric Get4/5 complex to the ribosome,

- the Get4/5 complex binding occurs with nanomolar affinity near the ribosomal exit tunnel in non-translating ribosomes,

- the binding site near the ribosomal exit tunnel is shared (at least) between the Get4/5 complex and the classic signal recognition particle (SRP),

- binding of the Get4/5 complex is a prerequisite for Sgt2 recruitment to the nascent/released TA protein at the ribosomal exit tunnel.

While the manuscript appears professionally written and is bearing a logic continuation from one line of evidence to the next some minor and major aspects should be addressed before the manuscripts warrants publication. In particular, figures 4 and 6 raised some major questions.

We thank Reviewer #4 for his/her positive evaluation of our manuscript. We are pleased that the Reviewer found our presentation of the extensive datasets accessible and “reader-friendly”. We also thank the Reviewer for suggesting thorough control experiments, and pointing out experimental data, which warranted improvement. The new results, we feel, further improved the manuscript.

Major points:

(1) Line 121ff: Was the experiment shown in Fig. 1a repeated? A quantification could help to estimate the pool of ribosome-associated and free Get4/5.[a)] Given the submicromolar affinity of Get4/5 for the 80S ribosome (line 166; Fig. 1b) and the 60x excess of ribosomes over Get4/5 complexes (300000 vs. 5000; line 384f.) the higher proportion of free Get4/5 should be discussed.[b)]

a) The experiment shown in **new Fig. 1a** was performed in triplicate. Quantification is now shown as **new Fig. 1b**.

b) The K_d of Get4/5 for ribosomes was determined using purified Get4/5 and ribosomes. No other components were present in the system. In the experiment shown in Fig. 1a the binding of Get4/5 or Get5 was determined in a cytosolic extract. We have now estimated the apparent K_d of Get4/5 in ribosome-binding experiments and discuss these data in **Supplementary Result 1**.

Of note, an increase of the apparent K_d values in total extract when compared to K_d values determined with purified components is also observed for SRP. The cellular concentration of SRP is approximately 8000 molecules per cell [13]. K_d values using purified SRP and ribosomes/RNCs were reported: SRP binding to non-SRP substrate RNCs K_d = 8 nM, RNCs exposing an SRP recognition sequence K_d = 0.05-0.38 nM, and SRP binding to non-translating ribosomes Kd = 71 nM [27]. However, in ribosome profiles (**Fig. 6d-f**) only about 1/3 of SRP is detected in the ribosomal fractions, possibly due to dissociation of the complexes during the ultracentrifugation procedure.

(2) Line 135f: Can the authors speculate why Get5 in absence of Get4 shows a much stronger ribosome association under low salt conditions (Fig. 1a)? Maybe this speculation can also be done with regard to the later finding that Get4 and Get5 bind via different interactions at the ribosomal surface (Figs. 2 and 3).

Reviewer #4 points out correctly that the occupancy of ribosomes with Get5 in the absence of Get4 (Δ*get4* + Get5↑) is higher than in wild type cells (Fig. 1a, compare lane 3 and 8). The most likely explanation is that Get5 was overexpressed in Δ*get4* (Fig. 1a). This view is consistent with the finding that the occupancy of ribosomes with Get4/5 is also enhanced when Get4↑Get5↑ is overexpressed in a wild type strain (Fig. 6a). As outlined in Supplementary Result 1, Get5 binds to cytosolic Sgt2, and Get4/5 binds to cytosolic Sgt2 and Get3. As the cellular concentration of Get5 or Get4/5 is increased upon overexpression, while the

concentrations of Sgt2 and Get3 are not, a larger fraction of overexpressed Get5 or Get4/5 is available for ribosome binding.

(3) Line 260ff:

- Was the experiment shown in Fig. 4b repeated?

The experiment shown in Fig. 4b was indeed repeated and we now show replica data leading to the same conclusion as **new Fig. 4d and 4e**. We now analyzed Get4 as well as Get5.

- In contrast to the wild type (Fig. 4b, upper panel), why is so much more Get4 immunoprecipitated in the negative control without FLAG in absence of Srp54 (Fig. 4b, lower panel)?

We sometimes observe background in the negative control; we do not fully understand why the background is increased in some experiments, but not in others. As we are aware of this potential problem, we perform minus FLAG-tag controls in each experiment. The background was very low in the experiment shown as **new Fig. 4e** (previous Fig. 4b lower panel).

What happens to the protein levels of Get4/5/Sgt2 in the Δsrp54 strain?

The expression level of Get4/5 and Sgt2 is not affected in the Δ*srp54* strain. We have included **new Supplementary Fig. 4f**, which shows the levels of Srp54, Sgt2, Get3, Get5, and the ribosomal marker Rpl31 in the translation extracts employed in the course of this study.

- What happens to SRP recruitment in a ΔGet4 strain? More efficient or earlier SRP recruitment in absence of GET would further substantiate the competition between the SRP and GET pathway for substrates with a TM helix near the tunnel exit.

- Additionally, a bona fide bitopic (type II) transmembrane protein could be used as control to demonstrate SRP over Get4/5 preference.

We thank Reviewer #4 for this suggestion. To test for competition between SRP and Get4/5 we employed Dap2, which is a well characterized substrate of SRP. We previously showed that SRP is recruited to RNCs carrying Dap2-60, which contains the TM domain of Dap2 inside of the ribosomal exit tunnel [28] (**new Fig. 4b**). Based on the referee´s suggestion we now tested binding of SRP to Dap2-60 in the presence or absence of Get4/5 (**new Fig. 4c**). The result reveals that the binding of SRP to RNCs-Dap2-60 was significantly enhanced in the absence of Get4/5. This observation supports the model of competition between Get4/5 and SRP with respect to ribosome binding.

(4) Line 286ff:

- In Fig. 4b lane 11 (RNCs-Sec22+60 construct) Get4 is not recruited to ribosome in the wild type strain and shows enhanced recruitment in the Δsrp54 strain. However, in the Get4 blot panel of Fig. 4e endogenous Get4 is found RNC associated (lane 1) and no substantial increase in recruitment of endogenous Get4 is seen in the Δsrp54 strain (lane 5).

- This discrepancy between Fig. 4b and 4e becomes even more prominent when adjusting for the Rps9 loading controls.

We have improved the data, which are now shown in the new versions of previous Fig. 4b upper panel (**new Fig. 4d**) and previous Fig. 4e (**new Fig. 4h**). In the experiment shown in **new Fig. 4d**, Get4 is detected on RNCs-Sec22 to RNCs-Sec22+60, however, the normalized amount of Get4 bound to RNCs-Sec22+60 is reduced 5-fold and of Get5 is reduced 10-fold when compared to RNCs-Sec22 (**new Fig. 4d**). Please note, that the 10-fold reduction observed for Get5 is likely an over-estimate, as the Get5 antibody is getting close to its detection limit when little Get4/5 is bound to RNCs. In the new version of previous Fig. 4e (now **new Fig. 4h**), a low amount of Get4/5 is detected bound to RNCs-Sec22+60 in the wild type. As expected based on the data shown in Fig. 4d and 4e, the amount of Get4/5 bound to RNCs-Sec22+60 is increased in the Δ*srp54* strain (**new Fig. 4h**).

(5) Line 300ff:

- Why is the Sec22+60xSgt2 crosslink a double band? Is Sgt2 interacting in different conformations/positions with Sec22? Could Sgt2 act as the yeast equivalent of mammalian BAG6 and recruit E3 ligases for ubiquitination of captured substrates (cf. line 435 and the data by Hegde and colleagues)?

Reviewer #4 is right, crosslinking between Sgt2 and the nascent chain at least at two different positions most likely accounts for the double-crosslink which we consistently observe. Crosslink products often migrate at a position different from the calculated molecular mass in SDS PAGE, because the polypeptide is not linear, but "branched". Migration can differ dependent on how the two polypeptides are connected. Other examples are Srp54 in Plath et al. Fig.1 [29] or Ssb, Ssz1, and Zuo1 in Zhang et al. Fig. 1 [15]. Sgt2 may act as a functional equivalent of mammalian BAG6. We also think along those lines for future experiments.

- Why is the Sec22+60xSgt2 crosslink appearing at different molecular weights in Fig. 5b (~60-80 kDa) and Fig. 5c (~90-110 kDa)?

We apologize for this mislabelling. We have now corrected the labelling of the marker in Fig. 5c.

(6) Line 306ff: Considering the circumstance that efficient Sgt2 interaction was demonstrated only using Sec22+60 (which is not an ideal representative of TA protein anymore) in conjunction with either SRP knockout or Get4/5 overexpression, the true biological relevance of the TA capture at the ribosomal exit tunnel in a wild type setting is somewhat obscure.

We clearly refer to the caveat with Sec22+60 in the manuscript (see also reply to Reviewer #3). However, there is no experimental procedure that would allow initiating crosslinking just when a released TA sequence appears at the tunnel exit. For this reason, we have employed the C-terminal extensions. As Reviewer #4 correctly states, addition of Get4/5 and absence of SRP enhances the crosslink of Sgt2 to Sec22+60. The crosslinking of Sgt2 to nascent Sec22+60 is close to the detection limit of the experiment. Detection of the crosslink product depends on the specific radioactivity of labelled ^{35}*S*[Met], which is not the same in all experiments due to fast decay. Please note, the Sec22+60xSgt2 crosslink is detected in the wild type extract, in Fig. 5c, lane 5. We also analyzed the effect of Get4/5 on the capture of released Sec22 by Sgt2 (Fig. 5e and 5f). The results fully support a model, in which TA capture in a wild type setting is enhanced by ribosome-recruitment of Sgt2.

(7) Line 336ff: Similar as in comment (6) the true nature and relevance of Sgt2 being recruited to translating ribosomes via Get4/5 in vivo (Fig. 6) is hard to grasp.

It is indeed challenging to precisely investigate the recruitment of Sgt2 to translating ribosomes. To test if Get4/5 recruits Sgt2 also in the wild type, we have further improved immunodetection of Sgt2 by increasing the loading to maximum and by increased incubation times with the first and secondary antibodies. With the improved method we show that a small fraction of Sgt2 is associated with ribosomes in a wild type cell lysate (**new Fig. 6a and new Fig. 6d-f**)

- The authors write (and cite their own published data) “previous analysis did not provide evidence for a ribosome-bound pool of Sgt2, it is possible that the amount of ribosome-bound Sgt2 was too low to be detected via immunoblotting”.

The reason for this discrepancy is that in in Zhang et al. 2016 [14] we analyzed each single fraction of the ribosome profile individually via immunoblotting. However, polysomes are spread out over 11 fractions, each of which contains only relatively little ribosomes (**new Fig. 6c**). After we found that Sgt2 binds to RNCs in a Get4/5-dependent manner, we reasoned that Sgt2 might be below the detection limit of the antibody in the diluted polysome fractions. To overcome this problem, we combined polysome fractions 9-19 (**new Fig. 6c**) and precipitated the material with TCA. After resuspension in sample buffer, combined fractions 9-19 were loaded into a single lane of an SDS-PAGE gel (indicated as 9-19 in **new Fig. 6d-f**). This is evident from the strong Rps9 and Rpl24 signals, which represent the collected polysomal ribosomes. The method is now described in detail in the **new paragraph *Ribosome profile analysis*** in the Methods section.

Due to this experimental approach we are now able to detect Sgt2 in polysome fractions even in the wild type strain (**new Fig. 6d**), while polysome fractions of the Δ*get4*Δ*get5* strain do not contain Sgt2 (Fig. 6f).

- The sucrose gradient fractionations show only the two opposing scenarios Get4/5 overexpression versus Get4/5 knockout, but no wild type.

As detailed above, we now included a wild type ribosome profile as **new Fig. 6d**. The analysis shows that Sgt2 is present in the polysome fractions of a wild type total yeast extract.

It appears that Sgt2 is recruited to translating ribosomes only by the non-physiological Get4/5 overexpression or deletion of SRP.

Please see above. Due to improvements of Sgt2 detection, we now find Sgt2 is bound to polysomes also in the wild type (**new Fig. 6a and new Fig. 6d**).

(8) Line 358f: “In this work, we show how newly synthesized tail anchored proteins are captured upon their emergence from the ribosomal tunnel.” All the work was done using Sec22 (or C-terminal Sec22 extensions). Hence, the authors should restrict the statement to singular “We show how a newly synthesized tail anchored protein, Sec22, is captured upon its emergence from the ribosomal tunnel.””

Sec22 is an authentic yeast TA protein, which was previously used as a model for TA protein targeting. In addition, we have now performed key experiments (recruitment of Get4/5 and

Get4/5-dependent recruitment of Sgt2) with RNCs that carry the yeast TA proteins Sed5 and Bos1. The result of the experiments shows that the conclusions that we made using Sec22 as substrate hold true also for other TA proteins (**new Supplementary Fig. 4i**).

(9) Line 979f: Given that Get4 seems to contact h46 and h47 inside the ribosomal tunnel the authors should also discuss the overlap with the NAC binding site in addition to the overlap with SRP (cf. Gamerdinger et al. 2019, doi:10.1016/j.molcel.2019.06.030). Based on this, how would the Sgt2 recruitment be altered in a ΔNAC strain?

We thank Reviewer #4 for this suggestion. We have included a paragraph highlighting the other ribosome-bound factors that are known to contact the Rpl35/Rpl26 region (see Results). The overlap of the binding sites suggests that there might be competition between Get4/5 and other ribosome-bound factors, including NAC. However, addressing these interesting questions is beyond the scope of this study.

The crosslinking site of Get4-HTP in helixes 46 and 47 of the 25S rRNA indeed lies partially within the ribosomal exit tunnel. However, it currently remains unclear whether Get4 predominantly resides on the surface of the ribosome or whether, analogous to the NAC complex described by Gamerdinger and colleagues, Get4 might probe the exit tunnel. At present we would rather refrain from discussing this possibility due to the lack of data in either direction.

(10) Line 1022: Why is the cross-reaction for endogenous Get4 mentioned for Fig. 4e not appearing in any other lane of the blot (e.g. lane 4 (wild type) or lane 5, 6, 8 (Δsrp54))?

We have repeated the experiment shown in former Fig. 4e, which is now shown as **new Fig. 4h**. The cross-reacting band was no longer detected.

(11) Line 1115: With the model for the capture of TM domains upon exit from the ribosomal tunnel (Fig. 7) the reader wonders how unique or universal is the C-terminal Sec22 TM domain compared to other tail-anchor sequences (cf. comment (8) and (6)).

We have now included experiments with two other TA protein (Sed5 and Bos1). The data (**new Supplementary Fig. 4i**) reveal that the TA sequences of Sed5 and Bos1 also recruit Sgt2 to ribosomes in a Get4/5-dependent manner.

(12) Line 1: Taking into consideration the data presented in Figs. 5 and 6 the title should more accurately reflect, that "The TA protein Sec22 can be captured by Sgt2 at the ribosomal tunnel exit upon overexpression of the GET mediator complex Get4/5".

We now present data showing that Sgt2 is bound to ribosomes also at endogenous Get4/5 concentration (**new Fig. 6a and 6d**) and that the mechanism pertains for the TA proteins Sed5 and Bos1 as well (**new Supplementary Fig. 4i**). We feel this justifies the more general title and the respective parts in the Discussion.

Minor points:

(13) Line 268: Consider deleting "(see also Discussion)"

done.

(14) Line 273: Consider deleting “(see Introduction)”

done.

(15) Line 274: that position Sgt2 -> that positions Sgt2

done.

(16) Line 275: “Pull down experiments” -> If not mistaken, Fig. 4b, d, e is based on α-FLAG M2 beads using an immobilized α-FLAG antibody and hence the authors should use the term immunoprecipitation instead of pull down.

done.

(17) Line 328: The knockout of the Δget3 strain/translation extract should be shown via immunoblot (in comparison to a control) in the supplement or main manuscript.

done. The blot is shown in **Supplemental Fig. 4f** together with all translation extracts employed in this study.

(18) Line 332: The bars in Fig. 5f could be arranged in same order as the lanes in Fig. 5e.

done.

(19) Line 426ff: Recent efforts from public data repositories provided a re-nomenclature of the mammalian TRC/GET components. Maybe the authors should adhere to this updated nomenclature throughout the manuscript.

We thank the reviewer for this advice, which makes it easier for the reader to understand the similarities and differences between yeast and mammals. We changed the nomenclature of the mammalian GET components according to UniProt throughout the manuscript.

We now use: BAG6, SGTA, UBL4A (instead of Ubl4a), GET4 (instead of TRC35), and GET3 (instead of TRC40).

(20) Line 917: While mathematically possible, it seems more sound to plot the two data points instead the s.e.m. (Fig. 1b).

We have now performed more replicates of the experiment shown in **new Fig. 1c** (previous Fig. 1b) and show all the data points instead of the s.e.m.

(21) Line 940: α-Get4 (His6Get5-XL in b), α-Rpl26

done.

(22) Line 941: (Rpl26-XL, Rpl35-XL in c)

done.

(23) Line 945: Period at end of sentence is in bold.

done.

(24) Line 1031: Delete sentence “Sgt2 binding to wild …”

done.

(25) Line 1035: While mathematically possible, it isn’t ideal to compare significance between groups with two data points. Given that more than two groups are compared and blotted an ANOVA analysis would be the better statistical test (Fig. 4f).

In the former Fig. 4f (now Fig. 4i) we compare groups of two, e.g., wild type with wild type + Get4/5↑ and Δ*srp54* with Δ*srp54* + Get4/5↑. We feel the statistical analysis is adequate.

(26) Line 1080: Given that more than two groups are compared and blotted an ANOVA analysis would be the better statistical test (Fig. 5f).

Reviewer #4 is correct. We have now performed ANOVA analysis for Fig. 5f, in which 3 groups are compared to each other.

(27) Line 1090: Rpl 31 (ribosomal marker) and α-Sse1 -> Rpl 31 (ribosomal marker), and α-Sse1

done.

(28) Line 1098: were pooled and aliquots -> were pooled and equivalent (??) aliquots

done.

References

1. Gautschi, M. *et al.* RAC, a stable ribosome-associated complex in yeast formed by the DnaK-DnaJ homologs Ssz1p and zuotin. *Proc. Natl. Acad. Sci. U S A* **98,** 3762-3767 (2001).
2. Hanebuth, M. A. *et al.* Multivalent contacts of the Hsp70 Ssb contribute to its architecture on ribosomes and nascent chain interaction. *Nat. Commun.* **7,** 13695 (2016).
3. Veisova, D. *et al.* The C-terminal segment of yeast BMH proteins exhibits different structure compared to other 14-3-3 protein isoforms. *Biochemistry* **49,** 3853-3861 (2010).
4. Hochscherf, J. *et al.* Development of a high-throughput screening-compatible assay to identify inhibitors of the CK2alpha/CK2beta interaction. *Anal Biochem* **468,** 4-14 (2015).
5. Kilkenny, M. L., De Piccoli, G., Perera, R. L., Labib, K. & Pellegrini, L. A conserved motif in the C-terminal tail of DNA polymerase alpha tethers primase to the eukaryotic replisome. *J Biol Chem* **287,** 23740-23747 (2012).
6. Huber, D. *et al.* SecA interacts with ribosomes in order to facilitate posttranslational translocation in bacteria. *Mol Cell* **41,** 343-353 (2011).
7. Kitamura, A. *et al.* Detection of substrate binding of a collagen-specific molecular chaperone HSP47 in solution using fluorescence correlation spectroscopy. *Biochem Biophys Res Commun* **497,** 279-284 (2018).
8. Zhang, Y. *et al.* NAC functions as a modulator of SRP during the early steps of protein targeting to the ER. *Mol. Biol. Cell* **23,** 3027-3040 (2012).
9. Gumiero, A. *et al.* Interaction of the cotranslational Hsp70 Ssb with ribosomal proteins and rRNA depends on its lid domain. *Nat. Commun.* **7,** 1-12 (2016).
10. Granneman, S., Petfalski, E., Swiatkowska, A. & Tollervey, D. Cracking pre-40S ribosomal subunit structure by systematic analyses of RNA-protein cross-linking. *EMBO J* **29,** 2026-2036 (2010).

11. Brüning, L. *et al.* RNA helicases mediate structural transitions and compositional changes in pre-ribosomal complexes. *Nat Commun* **9,** 5383 (2018).
12. Thoms, M. *et al.* The Exosome Is Recruited to RNA Substrates through Specific Adaptor Proteins. *Cell* **162,** 1029-1038 (2015).
13. Raue, U., Oellerer, S. & Rospert, S. Association of protein biogenesis factors at the yeast ribosomal tunnel exit is affected by the translational status and nascent polypeptide sequence. *J. Biol. Chem.* **282,** 7809-7816 (2007).
14. Zhang, Y. *et al.* Cotranslational intersection between the SRP and GET targeting pathways to the ER of Saccharomyces cerevisiae. *Mol Cell Biol* **36,** 2374-2383 (2016).
15. Zhang, Y. *et al.* The ribosome-associated complex RAC serves in a relay that directs nascent chains to Ssb. *Nat Commun* **11,** 1504 (2020).
16. Nelson, R. J., Ziegelhoffer, T., Nicolet, C., Werner-Washburne, M. & Craig, E. A. The translation machinery and 70 kd heat shock protein cooperate in protein synthesis. *Cell* **71,** 97-105 (1992).
17. Atkin, A. L. *et al.* Relationship between yeast polyribosomes and Upf proteins required for nonsense mRNA decay. *J. Biol. Chem.* **272,** 22163-22172 (1997).
18. Meyer, A. E., Hung, N. J., Yang, P., Johnson, A. W. & Craig, E. A. The specialized cytosolic J-protein, Jjj1, functions in 60S ribosomal subunit biogenesis. *Proc. Natl. Acad. Sci. U S A* **104,** 1558-1563 (2007).
19. Chiabudini, M. *et al.* Release factor eRF3 mediates premature translation termination on polylysine-stalled ribosomes in Saccharomyces cerevisiae. *Mol. Cell. Biol.* **34,** 4062-4076 (2014).
20. Pfund, C. *et al.* The molecular chaperone Ssb from Saccharomyces cerevisiae is a component of the ribosome-nascent chain complex. *EMBO J.* **17,** 3981-3989 (1998).
21. Gautschi, M. *et al.* The yeast N^{a}-acetyltransferase NatA is quantitatively anchored to the ribosome and interacts with nascent polypeptides. *Mol. Cell. Biol.* **23,** 7403-7414 (2003).
22. Wiedmann, B., Sakai, H., Davis, T. A. & Wiedmann, M. A protein complex required for signal-sequence-specific sorting and translocation. *Nature* **370,** 434-440 (1994).
23. Wegrzyn, R. D. *et al.* A conserved motif is prerequisite for the interaction of NAC with ribosomal protein L23 and nascent chains. *J. Biol. Chem.* **281,** 2847-2857 (2006).
24. Walter, P. & Blobel, G. Purification of a membrane-associated protein complex required for protein translocation across the endoplasmic reticulum. *Proc. Natl. Acad. Sci. U S A* **77,** 7112-7116 (1980).
25. Wild, K. *et al.* Reconstitution of the human SRP system and quantitative and systematic analysis of its ribosome interactions. *Nucleic Acids Res* **47,** 3184-3196 (2019).
26. Cho, H. & Shan, S. O. Substrate relay in an Hsp70-cochaperone cascade safeguards tail-anchored membrane protein targeting. *EMBO J* **37** (2018).
27. Flanagan, J. J. *et al.* Signal recognition particle binds to ribosome-bound signal sequences with fluorescence-detected subnanomolar affinity that does not diminish as the nascent chain lengthens. *J. Biol. Chem.* **278,** 18628-18637 (2003).
28. Berndt, U., Oellerer, S., Zhang, Y., Johnson, A. E. & Rospert, S. A signal-anchor sequence stimulates signal recognition particle binding to ribosomes from inside the exit tunnel. *Proc. Natl. Acad. Sci. U S A* **106,** 1398-1403 (2009).
29. Plath, K. & Rapoport, T. A. Spontaneous release of cytosolic proteins from posttranslational substrates before their transport into the endoplasmic reticulum. *J. Cell Biol.* **151,** 167-178 (2000).

REVIEWERS' COMMENTS

Reviewer #1 (Remarks to the Author):

My suggestions and concerns have all been adequately addressed and I now find this paper highly suitable for publication in Nature Communications.

Reviewer #2 (Remarks to the Author):

I think the authors definitely clarified and improved their manuscript. Most of my issues have been answered. However I feel that one would need a bit more information still to be convinced by the quality of the CRAC results, especially due to the fact sup fig3f shows similar profiles for wt and Get-HTP. The pie chart and the table gives more insight but is still presented in a biased way. Here are some important details to add in the text/legend:
-How many CRAC repeats have been made for wt and Get4-HTP (could you confirm there are at least 2 or 3)? Are the results shown an average, median or "most representative" replicate? could it be stated in the legend?
-Could all the classes of RNAs be added to the pie chart (especially the tRNAs, even if you decide to combine some of the RNA classes them for clarification).
-What is the percentage of unmapped/mapped reads and could this be added to sup figure 3?
-Have the reads been filtered for PCR duplicate removal and could this be stated in the method section? 24 cycles of PCR is not low for CRAC and seeing the profile of reads across RDN37 and the distribution among classes for filtered reads is important (even as a sup).
-Could you add the criteria used for Bowtie alignment?
Once these points are addressed, I will support the publication of the manuscript.

Reviewer #3 (Remarks to the Author):

Not sure what to say given that my major concern about their overarching conclusion has not been addressed by attempting new experiments along the lines I suggested previously. Instead, the authors resorted to polemic that at times suggest they themselves don't believe their model is actually falsifiable. It is certainly true that many great historical arcs in the land of ribosome-associated factors, like the SRP, have taken a lot of time and effort to reach their zenith but I guess I choose to draw more lessons from the nadirs of others, like the p180 ribosome receptor (yeah, p what?). There is already a lot of data in this manuscript and it would be no doubt a real effort to further define bona fide, ribosome non-binding Get4/5 mutants. However, I still think more biology-informed in vitro controls based on published reagents - e.g. in 6F, an Sgt2 C-terminal truncation in a get3 delete would control for the two known ways – direct TMD binding or indirect via Get3/4/5 - in which Sgt2 can associate with aggregated species; or in 5E, trying to cut Sgt2's ribosome association by providing Get5 in excess in vitro and seeing if this suppresses the enhanced TA pull-down of Sgt2 caused by the addition of Get4/5) would have helped me swallow their model, which remains more often than not supported by purely technical controls (e.g. epitope present/absent, high/low salt) and without complementary in vivo evidence.

Reviewer #4 (Remarks to the Author):

The revised manuscript by Zhang et al. titled "Ribosome-bound Get4/5 facilitates the capture of tail-anchored proteins by Sgt2 in yeast" addresses the early steps of recognizing tail-anchored proteins by a dedicated chaperone-like cascade, the GET system.

Compared to the original manuscript the authors provide further supporting data and addressed most,

if not all, of the comments and concerns that I (reviewer #4) initially raised. In particular, figures 4 and 6 that should demonstrate a physiological relevance of the early Get4/5 and Sgt2 recruitment to the ribosomal tunnel exit site received useful additions.

I very much welcome the detailed explanations and experimental evidence given for my initial comments 1-3. The authors provide more detail which includes the addition of Get5 blot panels to Fig. 4d and 4e, the switch from the Rps9 to Rpl31 as "loading reference" in those panels, supplementary Fig. 4f as well as incorporating Dap2-60 and its variant Dap2α-60 as further controls (new Fig. 4c). Thank you for that.

Similarly, adding the wildtype for comparison to Fig. 6 and the new layout of the figure allowing comparison of the three experimental conditions, the ribosome profiles and the analysis thereof are great. The evidence is much more compelling and fully addresses the previous comment 7. Thank you.

Providing additional data for the other tail-anchored proteins Sed5 and Bos1 (supplementary Fig. 4i) is excellent and negates comment 12. Well done.

Previous comment 4 and 10 referring to the discrepancy in old Fig. 4b and 4e (enhanced Get4 recruitment in in Δsrp54 in 4b, but not 4e) is no longer valid and the issue is fixed by the new blots provided in new Fig. 4d and 4h. However, I take the authors statements of "we have improved the data" and "the cross-reacting band was no longer detected" with a small grain of salt.

Despite the initial criticism on my end, the authors strengthened the manuscript, and I would like to see their results being published and endorse publication in Nature Communications.

Minor suggestions that do not require another round of review are:
- Fig. 4i has a faint grey box around the graph.
- Bar graphs (Figs. 1b, 4i, 5f, 6b) have the average as numbers next to the bar. Maybe it`s better to delete that (sorry, it did not occur to me during initial review).
- In Fig. 6d the labeling (Get4/5 cyt 76%) for the dark grey bar in the wild type condition is barely readable.
- The previous comment 16 suggested to change "pull-down" to "immunoprecipitation". The figure legend for new Fig 4c reads pull-down as well as the methods section "FLAG-tag pull down reactions". Feel free to use the terminology you prefer and change line 312 back to pull-down.

REPLY TO REVIEWERS' COMMENTS

Reviewer #1 (Remarks to the Author):

My suggestions and concerns have all been adequately addressed and I now find this paper highly suitable for publication in Nature Communications.

We are very happy to learn that Reviewer I now endorses publication of our manuscript.

Reviewer #2 (Remarks to the Author):

I think the authors definitely clarified and improved their manuscript. Most of my issues have been answered. However I feel that one would need a bit more information still to be convinced by the quality of the CRAC results, especially due to the fact sup fig3f shows similar profiles for wt and Get-HTP. The pie chart and the table gives more insight but is still presented in a biased way.

Here are some important details to add in the text/legend:
-How many CRAC repeats have been made for wt and Get4-HTP (could you confirm there are at least 2 or 3)? Are the results shown an average, median or "most representative" replicate? could it be stated in the legend?

Two biological replicates for the Get4-HTP CRAC experiment were performed, which are now both accessible in the GEO deposit (GSE151664). In the second dataset, the only prominent peak on the rRNAs is also in 25S-h46/47, which precisely overlaps with the peak shown in Fig. 3c and 3d. In the manuscript, a single representative dataset is shown and we now included this information in the Figure Legend.

-Could all the classes of RNAs be added to the pie chart (especially the tRNAs, even if you decide to combine some of the RNA classes them for clarification).

We included this information, which is now displayed as Supplementary Fig. 3h.

-What is the percentage of unmapped/mapped reads and could this be added to sup figure 3?

The percentages of mapped/unmapped reads is: Get4-HTP 39.5% mapped / 60.5% unmapped, WT 50.2% mapped / 49.8% unmapped. We have included this information now as Supplementary Fig. 3g. CRAC data were normalized (per million mapped reads) before further analysis.

-Have the reads been filtered for PCR duplicate removal and could this be stated in the method section? 24 cycles of PCR is not low for CRAC and seeing the profile of reads across RDN37 and the distribution among classes for filtered reads is important (even as a sup).

We thank the referee for pointing this out. We now describe how duplicate reads were removed from the sequencing data before further analysis, including the profile across RDN37 (Fig. 3c). The new text passage from the Methods section is also shown below (after the next point).

-Could you add the criteria used for Bowtie alignment?

We have now better described the criteria. The Bowtie alignment settings allowed all alignments containing no or a single mismatch. The results were then filtered to allow only reads containing a single T to C mutation. We now included this information into the Methods section, which is shown below.

Protein-RNA Cross-linking and analysis of cDNA (CRAC). *Yeast strains expressing HTP-tagged Get3, Get4, or Sgt2 from their genomic loci were analyzed by PAR-CRAC [33,69]. Cells were grown exponentially in low uracil media (10 mg/l uracil) supplemented with 100 μM 4-thiouracil before growth for an additional 4 h in the presence of 1 mM 4-thiouracil. 4-thiouridine (4sU)-containing RNAs were crosslinked to associated proteins using 600 mJ/cm^2 irradiation at 365 nm. Protein-RNA complexes were isolated under native conditions on IgG sepharose and then under denaturing conditions on Ni-NTA. A partial RNase digest was performed using RNace-IT, and co-purified RNAs were 5' labelled with [^{32}P] and were ligated to 3´ and 5´ sequencing adaptors. The 5´adaptor contained an NNNNNAGC unique molecular identifier sequence (UMI) to allow consolidation of multiple sequencing reads derived from the same RNA template. Complexes were separated by NuPAGE and transferred to a nitrocellulose membrane. RNAs were released from the membrane by Proteinase K digestion, isolated and reverse transcribed. The cDNA libraries were amplified by PCR (wild type: 35 cycles, Get4-HTP: 24 cycles) and subjected to Illumina deep sequencing. Sequencing reads were trimmed and quality controlled using Flexbar [70], reads shorter than 18 nucleotides were discarded. Identical sequencing reads containing the same UMI were collapsed to a single read. The remaining sequences were mapped to the S. cerevisiae genome using Bowtie 2 [71]. Alignments containing no, or a single, mismatch were allowed and reads were then filtered to retain only those reads containing a single T-C mutation induced by the presence of 4sU. For generating heat maps showing the number of reads mapping to different nucleotides on the secondary structure of the 25S rRNA [72] and the tertiary structure of the ribosome (PDB 4V88), programming scripts in the programming language Python (version 3.5.2) were used.*

Once these points are addressed, I will support the publication of the manuscript.

We thank Reviewer #2 for his/her helpful comments and for supporting the publication of our manuscript.

Reviewer #3 (Remarks to the Author):

Not sure what to say given that my major concern about their overarching conclusion has not been addressed by attempting new experiments along the lines I suggested previously. Instead, the authors resorted to polemic that at times suggest they themselves don't believe their model is actually falsifiable. It is certainly true that many great historical arcs in the land of ribosome-associated factors, like the SRP, have taken a lot of time and effort to reach their zenith but I guess I choose to draw more lessons from the nadirs of others, like the p180 ribosome receptor (yeah, p what?). There is already a lot of data in this manuscript and it would be no doubt a real effort to further define bona fide, ribosome non-binding Get4/5 mutants. However, I still think more biology-informed in vitro controls based on published reagents - e.g. in 6F, an Sgt2 C-terminal truncation in a get3 delete would control for the two known ways – direct TMD binding or indirect via Get3/4/5 - in which Sgt2 can associate with aggregated species; or in 5E, trying to cut Sgt2's ribosome association by providing Get5 in excess in vitro and seeing if this suppresses the enhanced TA pull-down of Sgt2 caused by the addition of Get4/5) would have helped me swallow their model, which remains more often than not supported by purely

technical controls (e.g. epitope present/absent, high/low salt) and without complementary in vivo evidence.

We thank Reviewer #3 for his/her suggestions for additional experiments. After carefully considering these suggestions we still think that these experiments are largely beyond the scope of the present paper. We would like to reiterate that our model is based on the results obtained by eight different in vivo and in vitro approaches, and at the same time matches with previously published data and accepted models of the later steps of the GET pathway. We think that at the current stage the experimental evidence is strong enough to present our model to the readership, and note that other referees seem to agree with us. There is no doubt that GET pathway models will be further tested and can be falsified in future experiments, as every scientific model.

Reviewer #4 (Remarks to the Author):

The revised manuscript by Zhang et al. titled “Ribosome-bound Get4/5 facilitates the capture of tail-anchored proteins by Sgt2 in yeast” addresses the early steps of recognizing tail-anchored proteins by a dedicated chaperone-like cascade, the GET system.

Compared to the original manuscript the authors provide further supporting data and addressed most, if not all, of the comments and concerns that I (reviewer #4) initially raised. In particular, figures 4 and 6 that should demonstrate a physiological relevance of the early Get4/5 and Sgt2 recruitment to the ribosomal tunnel exit site received useful additions.

I very much welcome the detailed explanations and experimental evidence given for my initial comments 1-3. The authors provide more detail which includes the addition of Get5 blot panels to Fig. 4d and 4e, the switch from the Rps9 to Rpl31 as “loading reference” in those panels, supplementary Fig. 4f as well as incorporating Dap2-60 and its variant Dap2α-60 as further controls (new Fig. 4c). Thank you for that.

Similarly, adding the wildtype for comparison to Fig. 6 and the new layout of the figure allowing comparison of the three experimental conditions, the ribosome profiles and the analysis thereof are great. The evidence is much more compelling and fully addresses the previous comment 7. Thank you.

Providing additional data for the other tail-anchored proteins Sed5 and Bos1 (supplementary Fig. 4i) is excellent and negates comment 12. Well done.

Previous comment 4 and 10 referring to the discrepancy in old Fig. 4b and 4e (enhanced Get4 recruitment in in Δsrp54 in 4b, but not 4e) is no longer valid and the issue is fixed by the new blots provided in new Fig. 4d and 4h. However, I take the authors statements of “we have improved the data” and “the cross-reacting band was no longer detected” with a small grain of salt.

Despite the initial criticism on my end, the authors strengthened the manuscript, and I would like to see their results being published and endorse publication in Nature Communications.

We are particularly pleased that Reviewer #4 finds our manuscript improved and recommends publication in Nature Communications. Thank you for your positive comments regarding the additional experiments and extensive work on Figures and text that we implemented to improve the manuscript.

Minor suggestions that do not require another round of review are:
- Fig. 4i has a faint grey box around the graph.

We have removed the faint grey box.

- Bar graphs (Figs. 1b, 4i, 5f, 6b) have the average as numbers next to the bar. Maybe it`s better to delete that (sorry, it did not occur to me during initial review).

We have deleted the average numbers next to the bars in Figs. 1b, 4i, 5f, and 6b.

- In Fig. 6d the labeling (Get4/5 cyt 76%) for the dark grey bar in the wild type condition is barely readable.

Indeed. Thank you for pointing that out. We improved the labeling of Fig. 6d.

- The previous comment 16 suggested to change “pull-down” to “immunoprecipitation”. The figure legend for new Fig 4c reads pull-down as well as the methods section “FLAG-tag pull down reactions”. Feel free to use the terminology you prefer and change line 312 back to pull-down.

Thank you. We changed line 312 back to FLAG-tag pull down reactions.
